# Electrical Conductivity and Permittivity of Porous Media: Origin, Measurements, and Implications

**DOI:** 10.1021/acsmeasuresciau.5c00070

**Published:** 2025-09-01

**Authors:** Farizal Hakiki, Chih-Ping Lin

**Affiliations:** a Disaster Prevention & Water Environment Research Center (DPWE), National Yang Ming Chiao Tung University (NYCU), Hsinchu 300, Taiwan; b Civil Engineering Department, National Yang Ming Chiao Tung University (NYCU), Hsinchu 300, Taiwan

**Keywords:** permittivity, complex permittivity, electrical conductivity, electrical properties, porous media, hydraulic properties, rock permittivity, soil permittivity, impedance spectroscopy, spectral induced polarization

## Abstract

Electrical sensing technologies have advanced our ability to infer and evaluate the hydraulic characteristics of porous media that are otherwise inaccessible to direct measurement. Such challenges are particularly prevalent in geo-porous materials such as rocks and soils found in remote regions, harsh environments, or beneath the Earth’s surface. Noninvasive sensing and characterization of these materials are indispensable preliminary steps for water–energy nexus activities, including extraction processes (e.g., desalination, groundwater utilization, fossil fuel and geothermal exploration and production) and mitigation efforts (e.g., sediment transport monitoring, contaminant management, and carbon or hydrogen capture, utilization, and storage). These electrical properties are measurable only if the material under investigation possesses an electrical charge and is polarizable. Electrical polarization refers to the relative displacement between positive and negative charges. This raises several critical questions: (i) In what ways can porous media acquire electrical charge and exhibit polarization? (ii) How can their electrical properties be measured both in laboratory and field environments? (iii) What frameworks can be used to interpret the observed electrical properties? (iv) How can we assess the reliability and validity of these interpretations in relation to the hydraulic and physical state of the porous media? This study aims to systematically investigate these questions through a comprehensive synthesis of existing literature and the integration of newly obtained experimental data.

## Graphical abstract

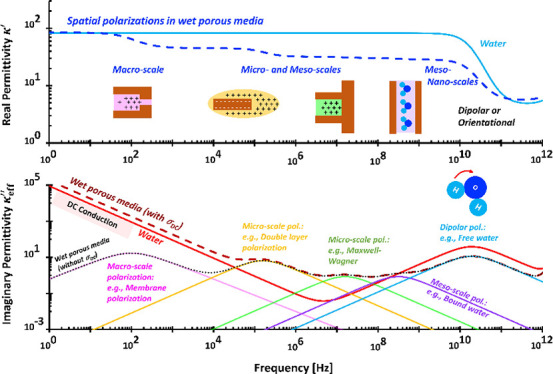

## 1 Introduction

Nondestructive sensing and characterization of porous media are essential prerequisites for activities related to the water–energy nexus, including both resource withdrawal (e.g., desalination, groundwater extraction, fossil fuel recovery, and geothermal exploration and production) and environmental mitigation (e.g., sediment transport assessment, remediation, contaminant control, and the capture, utilization, and storage of CO_2_ and hydrogen). Given the inaccessibility of many porous media; often due to extreme environmental conditions, remote locations, or subsurface burial, a direct characterization is frequently impractical. As a result, their electrical properties are commonly used as proxies for indirect sensing and assessment.

Electrical properties in wet porous media provide critical information about hydraulic behavior, pore fluid composition, and fluid–solid interfacial interactions. Consequently, the measurement of electrical conductivity and permittivity is widely employed across the chemistry and geoscience disciplines to support diverse applications in environmental monitoring and engineering design. These include: groundwater contamination observations,^1^ water-in-oil emulsions investigation,^2^ membrane fouling inspection,^3^ polymer coagulation monitoring,^4^ water/wastewater characterizations,^5^ metal content searches,^6^ fluid content predictions,^7^ subsurface mapping,^8^ leakage detections,^9^ and construction evaluation.^10^

Interfacial phenomena and properties arising from the interactions between porous media solids and pore fluids are effectively characterized through their interfacial electrical signatures., e.g., electrical double layer,^11^ zeta potential,^12,13^ streaming potential/current,^14^ surface charge,^15,16^ and surface conduction.^17^

Electrical properties become measurable in porous media due to the presence of free or bound charges and the occurrence of relative charge displacements, commonly referred to as electrical polarization (Figure 1). Prior to the full establishment of a polarized state, the movement of charges can contribute to electrical conductivity through mechanisms involving either the physical displacement of charge carriers, such as ion migration, or localized hopping processes that do not entail net mass transport.^18–20^ Consequently, permittivity which characterizes a material’s capacity to polarize in response to an applied electric field is intrinsically linked to electrical conductivity. Electrical conductivity, in turn, quantifies the material’s ability to support the flow of electric current.

**Figure 1:**
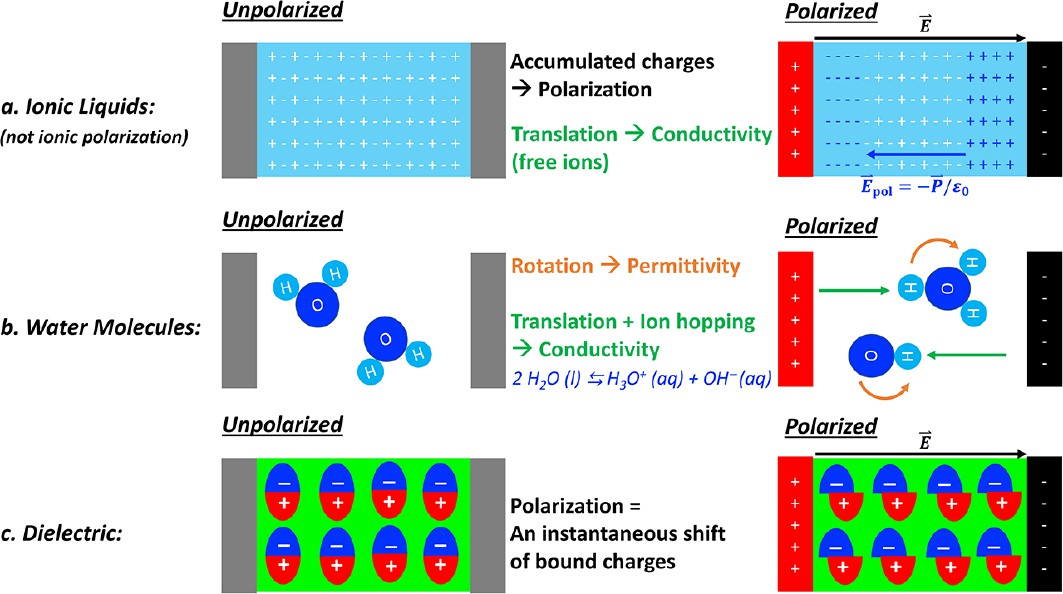
Illustration of charge movements under polarization conditions in various materials: (a) Ionic liquids (polarized charges q). A relative separation of ions remains evident in the vicinity of the electrodes yet it is electrically neutral in the bulk region. A label of "not ionic polarization" refers to neither classical nor vibrational ionic polarizations.(b) Water molecules. (c) Dielectric (Insulator). Blue arrow in (a) indicates a polarized field E_pol_= −P/ε_0_ that against the externally applied electrical field E (black arrow).

Porous media span scales from nanometers to kilometers, posing challenges for measuring electrical conduction and polarizability across such ranges. Advances in sensing and electronics now allow high-resolution, multiscale, and multifrequency measurements. By selecting frequencies based on targeted spatial scales or skin depths, researchers can optimize resolution and depth. Higher frequencies yield finer resolution but shallower penetration. Figure 2 illustrates common lab and field methods across frequencies. AI integration further improves data interpretation^21^ and accelerates computation.^22,23^

**Figure 2:**
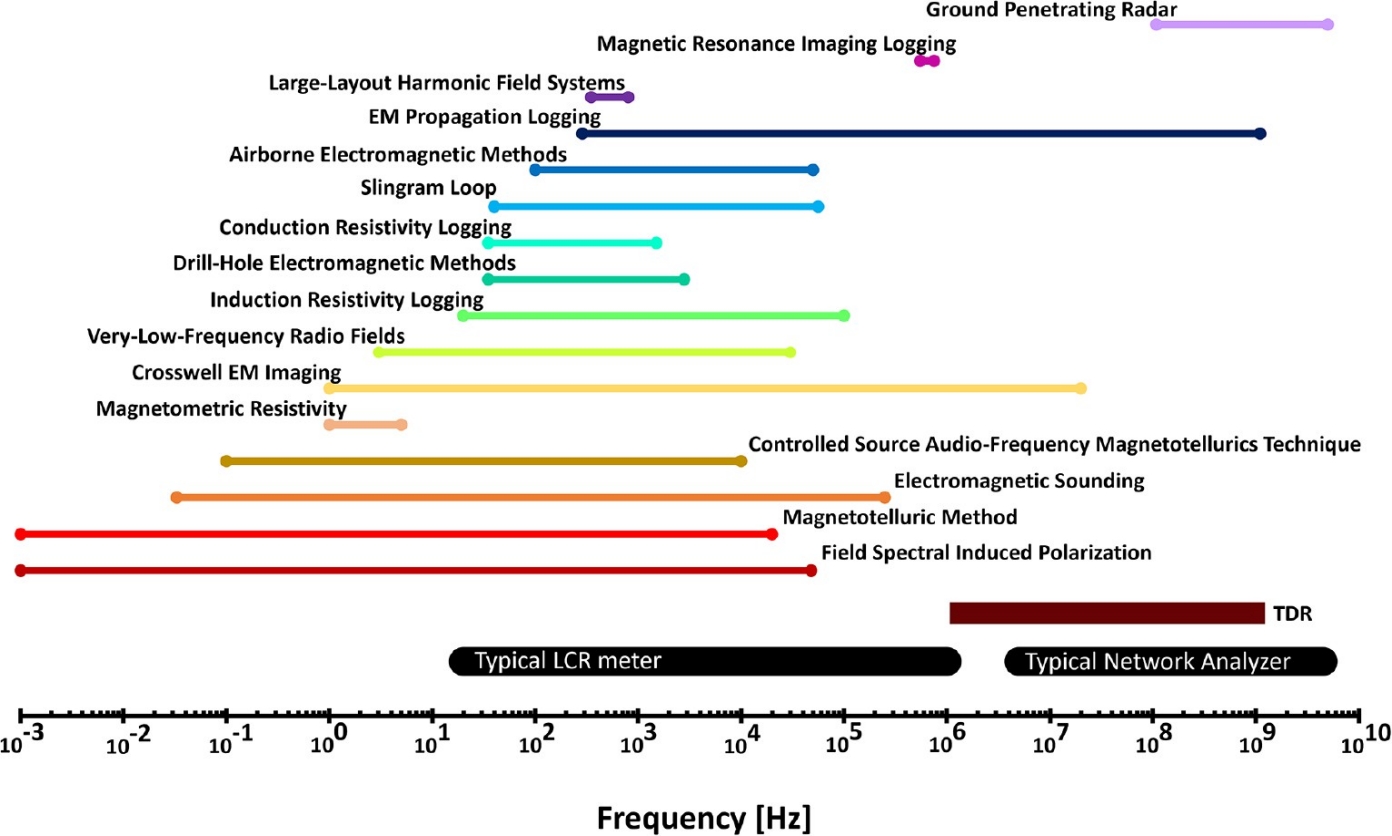
Examples of measurement techniques to measure electrical properties and their typical frequency ranges.

Despite decades of research, key challenges remain in measuring subsurface electrical properties. These include the overuse of Archie’s equation, the trade-off between resolution and depth, and misinterpretations when linking electrical and hydraulic properties.^24–26^ While many correlations exist, they do not imply causation.^26,27^ Electrical properties in wet porous media are highly sensitive to pore-fluid composition. This study explores GHz-range frequencies and beyond, where fluid-specific features such as orientational relaxation frequency *f*_*c*_ ^28^ and chemical bonding signatures enable more precise characterization.^29,30^

This review focuses on the electrical properties of porous media and pore fluids, specifically those related to electrical conduction and polarization, as characterized by conductivity, resistivity, and permittivity under isothermal conditions. These parameters are intrinsically interrelated, as they describe the same underlying physical phenomena. However, the choice of terminology, whether conductivity, resistivity, or permittivity, is often guided by dominant frequency-dependent behaviors or established conventions within specific scientific communities.

The detailed objectives of this article are as follows:

- to clearly define and identify the origins of charges, electrical conduction, and polarizations that occur in pore fluids and porous media
- to prudently examine the possible measurement artifacts that obscure the true material properties and hinder certain phenomena; consequently, we call attention to the methods for measuring electrical properties that are either electrode-based or electrodeless (wave propagation), both in the laboratory and in the field
- to critically evaluate which hydraulic properties have a direct influence and causality on the electrical properties and which do not. To accomplish this goal, we revisit the formulations that construct the hydraulic and electrical properties and compile hundreds to thousands of previously published data points and our own measurements data to verify the relationships
- to highlight the challenges and potential technological contributions that can be made to reduce the uncertainties in measuring and interpreting the electrical properties of porous media.

## 2 Conductivity vs Permittivity Equations

The complex electrical conductivity σ_eff_^*^ governs the current density ***J*** (current *I* per unit cross-sectional area ***A***), following Ohm’s law: ***J*** = σ_eff_^*^ ***E***. Here, the subscript “eff” denotes the material’s effective conductivity. In circuit form by Kirchhoff's reformulation, this becomes *I* = σ_eff_^*^ *A* Δ*V*/*L*, where Δ*V*/*L* is the electrical potential gradient (Figure 3a). This expression is analogous to Fourier’s law and Darcy’s law (Figure 3b). The complex impedance Z*, describing frequency-dependent resistance, is given by $Z^{*}=\frac{\Delta V}{I}=\frac{1}{\left(A/L\right)\sigma_{\mathrm{eff}}^{*}}=\frac{1}{\beta \sigma_{\mathrm{eff}}^{*}}$ , where geometric factor β is the ratio between cross-sectional area *A* and sample length *L*.

**Figure 3:**
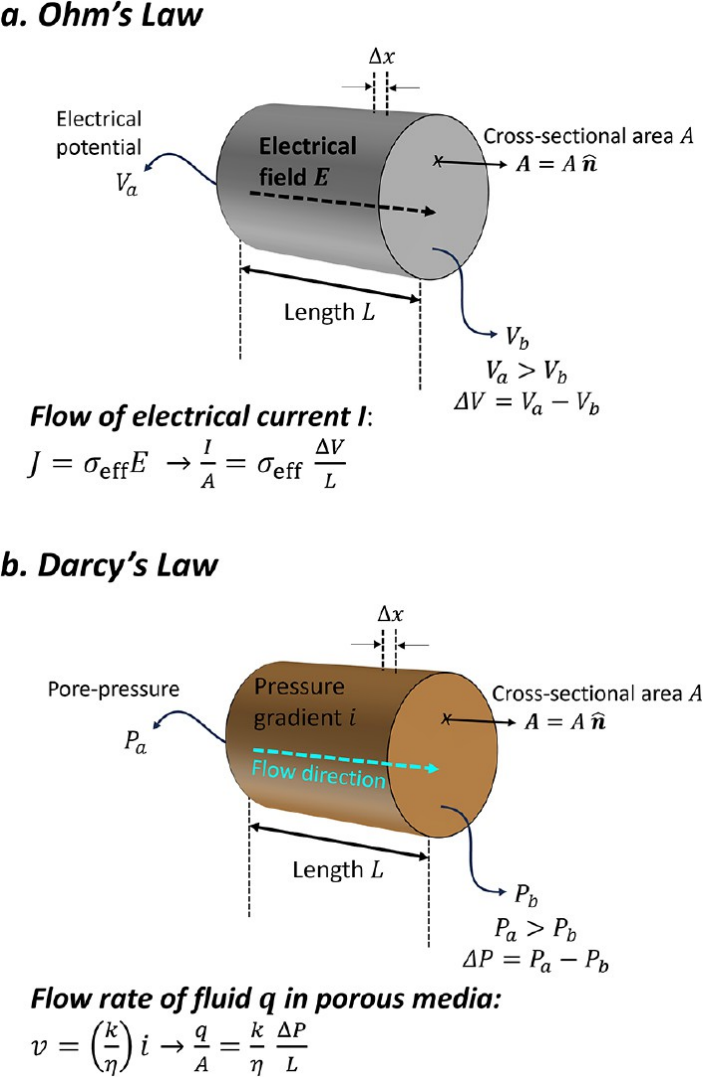
Ohm’s and Darcy’s law comparison. Flow equations are presented in scalar notation.

In controlled settings with sustained fields (e.g., electrokinetic remediation or microfluidic analogues), electrophoretic and dielectrophoretic transport may be significant. In typical soil physics and geophysical surveys, where fields are transient or broadband, a uniform field ***E*** and Ohm’s law are sufficient.

According to Maxwell’s equations, the total current density ***J*** includes conduction (***J*_*C*_** =σ_*DC*_ ***E***) and displacement (*J*_*D*_ **=**∂ ***D***/∂*t*) components: ***J*** =σ_*DC*_ ***E +*** ∂ ***D***/∂*t* where σ_*DC*_ is the direct current conductivity and ***D*** = ε* ***E*** is the charge displacement field. The complex permittivity (ε* = ε^′^ – *j*ε^″^) captures the material’s polarizability, including both energy storage and loss.^31^

Assuming a time-harmonic field ***E*** = ***E*_0_** exp (*j*ω*t*), the displacement current becomes ***J*_*D*_** = *j*ωε* ***E***. Thus, the total current density is ***J =*** σ_eff_^*^ ***E*** = (σ_*DC*_ + *j*ωε*) ***E*** with σ_eff_^*^ = σ_*DC*_ + *j*ωε* = *j*ωε_eff_^*^ where $\varepsilon_{\mathrm{eff}}^{*}=\varepsilon^{\prime }-j\varepsilon_{\mathrm{eff}}^{\prime \prime }=\varepsilon^{\prime }-j\left(\varepsilon^{\prime \prime }+\frac{\sigma_{DC}}{\omega }\right)$ .^31^ Because complex permittivity ε_eff_^*^ and conductivity σ_eff_^*^ are related through σ_eff_^*^ = *j*ωε_eff_^*^, both can be used to characterize material polarizability (Figure 4 and Figure 5). At high frequencies, σ_*DC*_ is negligible so that σ* = *j*ωε*. The relationships among key frequency-dependent electrical properties are summarized as (no σ_*DC*_ inclusion):

$$\{\begin{array}{c} Z^{*}=\frac{1}{\sigma^{*}\beta }=\frac{\rho^{*}}{\beta }=Z^{\prime }+jZ^{\prime \prime }\\ \rho^{*}=\rho^{\prime }+j\rho^{\prime \prime }\\ \sigma^{*}=\frac{1}{\rho^{*}}=\sigma^{\prime }+j\sigma^{\prime \prime }\\ \sigma^{*}=j\omega \varepsilon^{*}\\ \varepsilon^{*}=\kappa^{*}\varepsilon_{0}\\ \kappa^{*}=\kappa^{\prime }-j\kappa^{\prime \prime }\\ M^{*}=\frac{1}{\kappa^{*}}=M^{\prime }+jM^{\prime \prime }\\ \chi_{e}^{*}=\kappa^{*}-1 \end{array}$$ 1

where Z* is the complex impedances, ρ* is the resistivity, σ* is the conductivity, ε* is the permittivity, κ* is the relative permittivity, M* is the electrical moduli, χ_e_^*^ is the electrical susceptibility, β is the geometric factor and ε_0_ is the vacuum permittivity (8.85 × 10^–12^ F/m). The in-phase conductivity σ^′^ = ωε_0_κ^″^, the effective form σ_eff_^′^ = ωε_0_κ_eff_^″^ = σ^′^ + σ_*DC*_, and the quadrature term σ^*"*^_eff_ = σ^″^ = ωε_0_κ^′^. Over wide frequency ranges, κ_eff_^*^ includes both polarization κ^″^ and conduction effects $\frac{\sigma_{DC}}{\omega \varepsilon_{0}}$ .^26^

**Figure 4:**
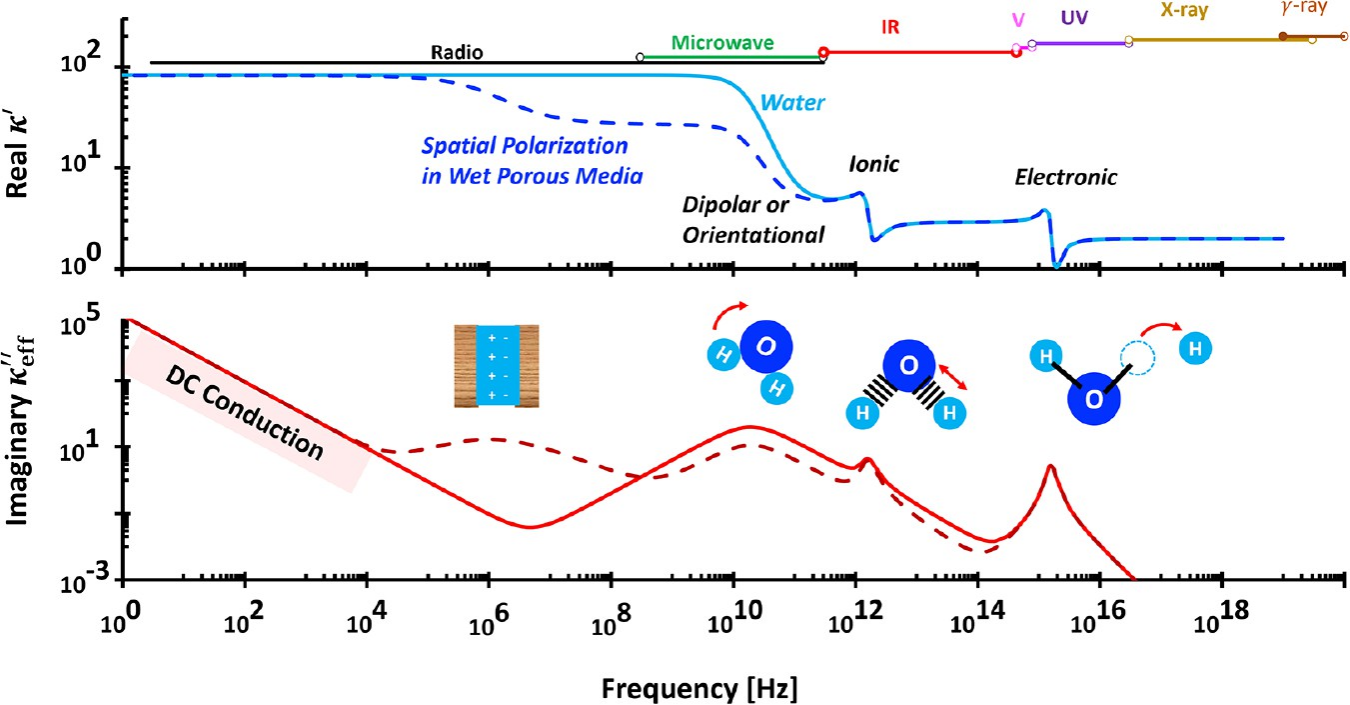
Typical wideband complex permittivity spectra. The fitting parameters for the liquid water (light blue and red lines): conduction: σ_*DC*_ = 5 × 10^–6^ S/m; orientational polarization: κ_*U*_ = 80, κ_*L*_ = 2, τ = 8.2 × 10^–12^ s, δ = 1; vibrational ionic polarization: κ_*U*_ = 1.7, κ_*L*_ = 0, τ = 1 × 10^–15^ s, δ = 1.7, and electronic polarization: κ_*U*_ = 0.9, κ_*L*_ = 0, τ = 1 × 10^–17^ s, δ = 1.8. The fitting parameters for the wet porous media (dashed darker blue and red lines): conduction: σ_*DC*_ = 5 × 10^–6^ S/m; spatial or interfacial polarization: κ_*U*_ = 55, κ_*L*_ = 0, τ = 1.59 × 10^–7^ s, δ = 0.7; orientational polarization: κ_*U*_ = 24, κ_*L*_ = 2, τ = 8.2 × 10^–12^ s, δ = 1; vibrational ionic polarization: κ_*U*_ = 1.7, κ_*L*_ = 0, τ = 1 × 10^–15^ s, δ = 1.7, and electronic polarization: κ_*U*_ = 0.9, κ_*L*_ = 0, τ = 1 × 10^–17^ s, δ = 1.8. Assumed porosity: 0.3. This implies to the orientational polarization: κ_*U*_ = 0.3 × 79 ∼ 24.

**Figure 5:**
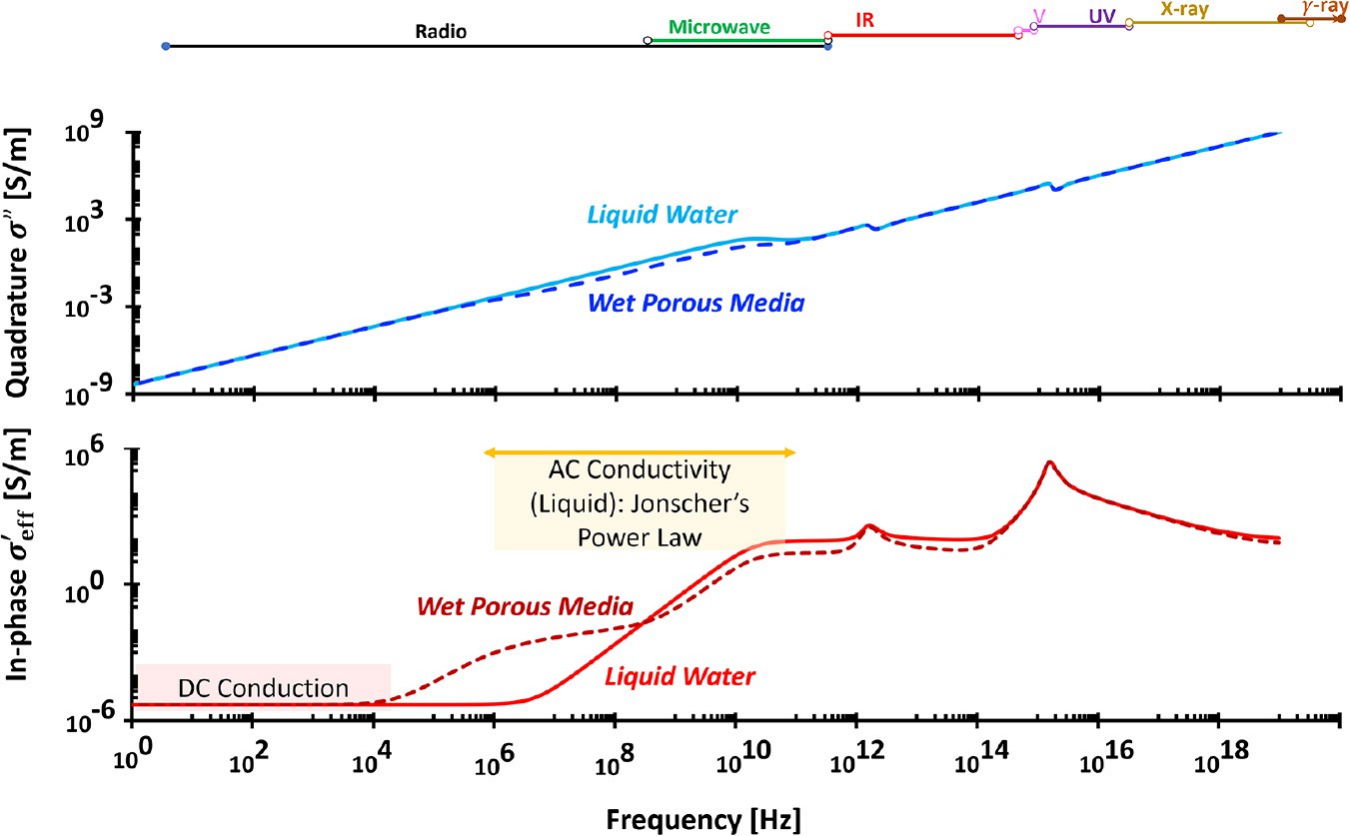
Typical wideband complex conductivity spectra. Fitting parameters are identical to those used in Figure 4.

A widely used equation to fit the complex permittivity spectra is Havriliak–Negami model:^32^

$$\kappa_{\mathrm{eff}}^{*}=-j\frac{\sigma_{DC}}{\omega \varepsilon_{0}}+\overset{N}{\underset{i=1}{\sum }}\left[\kappa_{L_{i}}+\frac{\kappa_{U_{i}}-\kappa_{L_{i}}}{{\left[1+{\left(j\omega \tau_{i}\right)}^{\delta_{i}}\right]}^{\beta_{i}}}\right]$$ 2

where τ_*i*_ = 1/(2π*f*_*c*, *i*_) is the relaxation time, the β **controls the asymmetry** of the dielectric loss peak κ^″^ at the polarization frequency *f*_*c*_, while δ **governs the width** (broadening) for each polarization *i*. If δ = 1 and β ≠ 1, the equation is called the Cole-Davidson model.^33^ If δ ≠ 1 and β = 1, the equation reduces to the Cole–Cole model.^34^ If both δ = 1 and β = 1, the equation reduces to the Debye model,^35^ suitable for efficient wideband computation.^36^

We realize that complex permittivity and conductivity are convertible through σ_eff_^*^ = *j*ωε_0_κ_eff_^*^ regardless knowing the representative circuit model. However, we choose Cole–Cole model to describe selected polarization dynamics across frequencies (Figure 4) because Cole–Cole model exhibits an analogous form with the most general circuit model, i.e., a parallel configuration between resistor and constant phase element (CPE).^37^

In-phase effective conductivity values approximate the DC conductivity (σ^′^_eff_ ≈ σ_*DC*_) at low frequencies (<1 kHz) and is rising with frequency following Jonscher’s power law:^38^ σ^′^_eff_ ∝ ω^*p*^ (Figure 5). The Kramers–Kronig relation $\kappa^{\prime \prime }\propto \frac{\partial \kappa^{\prime }}{\partial \ln\omega }$ further links real and imaginary permittivity at negligible DC conduction.^39–41^ The choice to show conductivity, resistivity, or permittivity is often considered based on frequency response characteristics and conventions within a specific research field.

## 3 Origin of Charges

### 3.1 Charge Development

Charges in porous media arise from electron clouds in solids, free ions in pore fluids, and counterions at solid–fluid interfaces (Figure 6a). Dry porous materials like geomaterials, bone, or cement are poorly conductive and nearly nonpolarizable due to absence of significant charge mobility. However, exposure to fluids induces surface charging via:^42^ (1) chemical reactions (e.g., redox, dissolution, ion adsorptions, protonation), (2) isomorphous substitution, and (3) surfactant adsorption (Table 1). These mechanisms underscore the importance of fluid composition and interfacial interactions in determining conductivity and polarizability.

**Figure 6:**
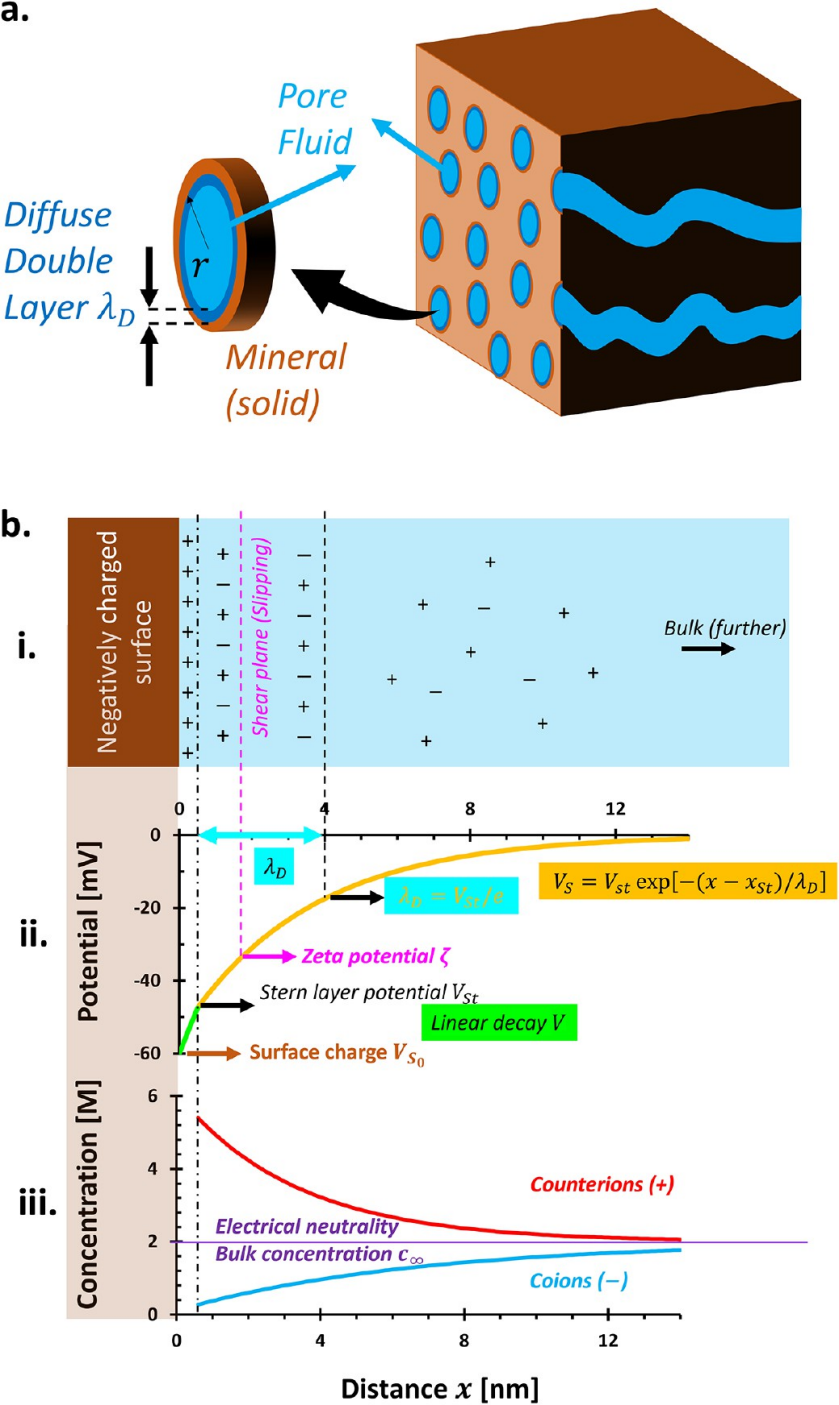
Origin of charges in porous media. (a) Charge-bearing components: solid, pore fluid, and their interactions through the electrical double layer. (b) Charge distributions (i) at the mineral–fluid interface and their (ii) surface potential *V_S_* and (iii) ionic concentrations. Stern layer distance *x_St_* depicted is 0.5 nm. The diffuse double layer *λ_D_* is 3.5 nm.

**Table 1:**
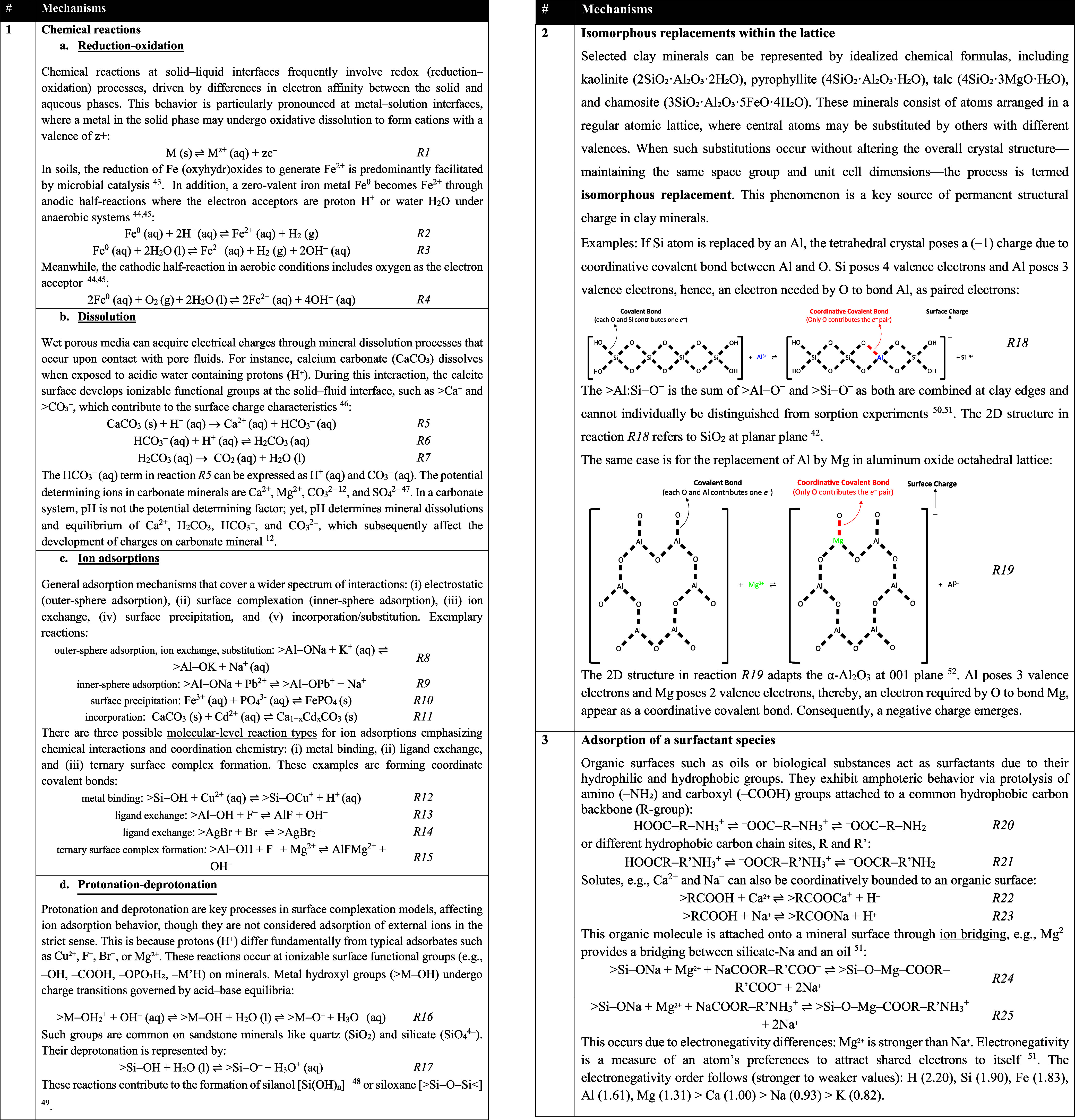
Origin of Charges in Solid Surfaces Once Exposed to a Pore Fluid^43,44,45,46,47,48,49,50,51,52^

Charging on wet soils mainly occurs via surface complexation of hydroxylated Si, Al, and Fe oxides, whose > M–OH groups gain or lose protons, creating pH-dependent charges^42^ (Table 1). In contrast, dry mineral ores and tailings charge through electronic conduction in conductive minerals like sulfides and graphite, enabling electron movement without moisture.^53–56^

### 3.2 Surface Charges and Electrical Double Layer

Solid–fluid interactions generate surface charges on minerals, attracting counterions from the pore fluid and forming an electrical double layer (EDL). These counterions cluster near the surface in an immobile Stern layer, followed by a diffuse region where their concentration decays with distance (Figure 6b-i).

The **Stern potential** *V*_*St*_ is not directly measurable but is often approximated by the **zeta potential** ζ, located at the shear plane where ions begin to move with the fluid. This plane is not sharply defined and does not reflect the full thickness of the diffuse double layer λ_*D*_.^42^

The Debye–Hückel length λ_*D*_ quantifies the thickness of the diffuse layer where the potential decays to 1/*e* (∼0.368) of *V*_*St*_ (Figure 6b-ii). While often used interchangeably, the EDL includes both the Stern and diffuse layers, whereas λ_*D*_ only defines the latter:^51,57^

$$\lambda_{D}=\sqrt{\frac{\kappa^{\prime }\varepsilon_{0}k_{B}T}{2N_{A}e_{0}^{2}I}}$$ 3

where *k*_*B*_ is the Boltzmann constant (1.38 × 10^–23^ m^2^.kg/s^2^/K), *T* is the temperature in Kelvin, *N*_*A*_ is the Avogadro’s number (6.02 × 10^23^), *e*_0_ is the elemental electron charge (1.602 × 10^–19^ C), and *I* is the ionic strength and is expressed by $I=\frac{1}{2}\overset{n_{I}}{\underset{j}{\sum }}c_{i,j}z_{j}^{2}$ , summation of *n*_*I*_ number of ionic species’ concentrations *c*_*i*_ [mol/L] and charge valences *z*. Typical Stern layer thickness *x*_*St*_ is 0.2–1 nm and the diffuse double layer λ_*D*_ is about 2–183 nm in clay minerals.^57–59^

The **surface potential** *V*_*S*_0__, though not directly measurable, can be estimated from surface charge data.^42^ In dry conditions, mineral surfaces typically carry negative charges. Upon wetting, these surfaces attract counterions (e.g., Na^+^, K^+^) and repel co-ions (e.g., Cl^–^), forming a diffuse layer that restores charge neutrality (Figure 6b-iii).

In fine sediments, the **zeta potential** ζ is measured via light scattering instruments (e.g., zetasizers).^51^ In intact porous media, it is indirectly calculated using the **Helmholtz–Smoluchowski equation**:^17,60–62^

$$\{\begin{array}{c} \zeta =\frac{\eta \sigma_{f}}{\kappa_{s}\varepsilon_{0}}C_{sp}\\ C_{sp}=\frac{\Delta V_{sp}}{\Delta p} \end{array}$$ 4

This requires data on fluid viscosity η, fluid conductivity σ_*f*_, fluid permittivity κ_*s*_, and streaming potential coupling coefficient *C*_*sp*_ calculated from the recorded streaming potential Δ*V*_*sp*_ generated by counterion movement under a pressure gradient Δ*p* (Figure 7a and b).

**Figure 7:**
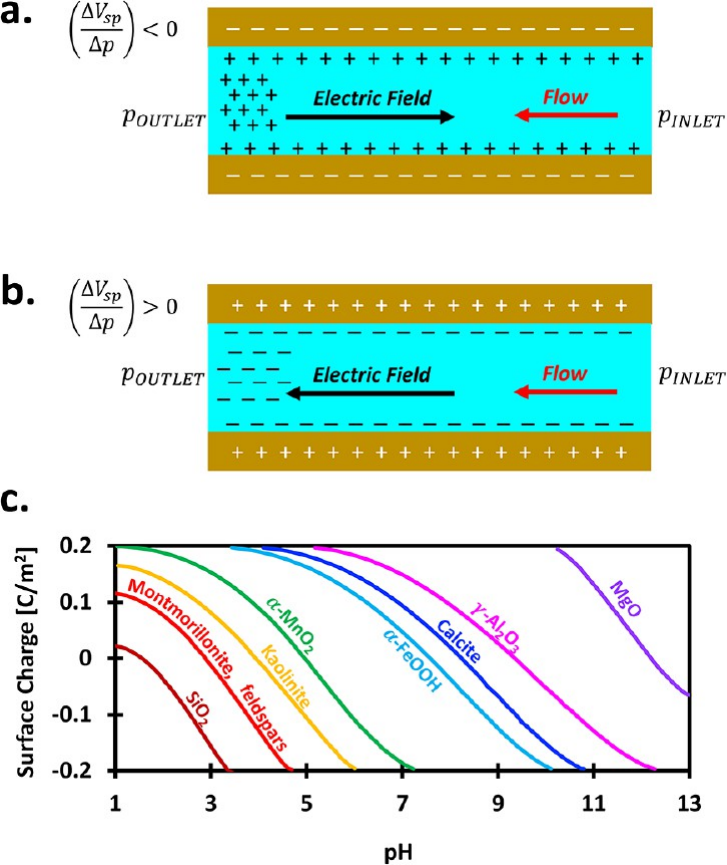
Zeta potential measurements and typical surface charges. Measurement set up is *p*_INLET_ > *p*_OUTLET_. (a) Measurement case when the surface charge is negative, typically occurs in high pH (pH > PZC); (b) measurement case when the surface charge is positive, typically occurs in low pH (pH < PZC); and (c) typical surface charges of selected minerals in wide pH range (data source: ref 42). Note: PZC is a point of zero charge, i.e., pH when the surface charge is zero.

Zeta potential and mineral surface charge are pH-dependent (Figure 7c). While |ζ| decreases with increasing ionic concentration *c*_*i*_,^63^ this reflects the stronger influence of the coupling coefficient *C*_*sp*_, which empirically follows |*C*_*sp*_| ∝ *c*_*i*_^*n*_*c*_^, where *n*_*c*_ < 0.^64^

The Helmholtz–Smoluchowski model assumes negligible surface conduction and is valid mainly at high ionic strength (*I* ≥ 0.1 M)^65^ or large pores (pore radius-to-diffuse layer ratio *r*/λ_*D*_ ≥ 200).^66,67^ At low ionic strength or small pores, surface conduction becomes significant, and corrections are needed to account for electrokinetic effects near charged surfaces^65,68^

## 4 Origin of Electrical Polarizations

### 4.1 Definition of Polarization

An applied electric field ***E*** exerts a force ***F*** = *q* ***E*** on a charge *q*, causing charged particles within a molecule to displace slightly from their equilibrium positions. This displacement leads to the induction or reorientation of a dipole moment **μ**_*E*_, resulting in polarization of the material. If the molecule already possesses a permanent dipole moment (as in polar molecules; Figure 1a and b), the field tends to align it. If the molecule is nonpolar, the field can induce a dipole by distorting the electron cloud (Figure 1c). The dipole moment is defined as **μ**_*E*_ *=* ∑ *q*_*i*_ ***d* ^***_i_*, where each charge *q*_*i*_ is weighted by its relative distance ***d* ^***_i_* from a chosen reference point (typically the center of mass or charge). Alternatively, **μ**_*E*_ = α_*e*_ ***E*_*loc*_**, where α_*e*_ is electrical polarizability and ***E*_*loc*_** is the local field induced by nearby charges, even without an external field ***E***.

Polarizability α_*e*_ and the local electric field ***E*_*loc*_** serve to connect molecular-scale interactions with macroscopic electric fields. In the following discussion, we characterize materials primarily by their relative permittivity κ_*s*_, which is more accessible experimentally. Given κ_*s*_, the α_*e*_ can be estimated using models such as the Clausius–Mossotti relation.^69–71^

Polarization density ***P*** (or maximum ***P*_0_**) is linearly proportional to the applied field ***E*** when bound charges in atoms or molecules shift slightly.^71^ Thus, ***P*** = χ_e_^*^ε_0_ ***E***, where χ_e_^*^ is an electrical susceptibility, related to the relative permittivity $\kappa^{*}=\frac{\varepsilon^{*}}{\varepsilon_{0}}=1+\chi_{e}^{*}$ . For example, water (χ_*w*_∼77) is more polarizable than air (χ_*air*_ = 0). Zero susceptibility means no dipoles formed under ***E***. In addition, earlier literatures derive $\chi_{e}=\frac{n_{d}\alpha_{e}/\varepsilon_{0}}{1-n_{d}\alpha_{e}/3\varepsilon_{0}}$ .^69,71^ Thus, material polarizability can be characterized via permittivity κ, electrical susceptibility χ_*e*_, or electrical polarizability α_*e*_, and related to complex conductivity σ* and electrical moduli M*.

Figure 1a illustrates a macroscopic-scale polarization field *E*_**pol**_ *= – P*/ε_0_ arising from polarized materials. The charge displacement field ***D*** combines the external field ***E*** and the polarization ***P*** to become:

$$D=\varepsilon_{0}E+P=\varepsilon_{0}E+\chi_{e}^{*}\varepsilon_{0}E=\left(1+\chi_{e}^{*}\right)\varepsilon_{0}E=\kappa^{*}\varepsilon_{0}E$$ 5

where *D* is associated with the free charges and *P* corresponds to the bound charges.^71^

A higher number of electron clouds, ions, and charged molecules within a representative elementary volume, whether free or loosely bound, may contribute to greater polarizability, and consequently, higher permittivity. However, this does not inherently imply higher electrical conductivity, which depends on the presence and mobility of free charge carriers. Yet, at low frequency, materials with higher electrical conductivity exhibit a greater effective imaginary permittivity κ_eff_^″^ (see eq 2).

Why in a representative elementary volume? Intrinsically, a maximum polarization density ***P***_0_ of a medium is described by molecular density *n*_*d*_ and dipole moment **μ**_*E*_:^70–72^

$$\{\begin{array}{c} P_{0}=n_{d}\mu_{E}\\ n_{d}=\frac{\rho N_{A}}{M_{r}} \end{array}$$ 6

The molecular density *n*_*d*_ represents the number of atoms or molecules per unit volume (e.g., #/mL, #/L, or #/m^3^). It is derived from the macroscopic density ρ [mass/volume], Avogadro’s number *N*_*A*_ [#/mol], and molecular mass *M*_*r*_ [mass/mol]. Accordingly, the polarization density ***P*** and ***P*_0_** quantifies the volumetric contribution of individual dipole moments **μ**_*E*_ from all charged species.

Polarization stores electrostatic energy, which is released during relaxation as the system returns to equilibrium. Relaxation, though seemingly postpolarization, reflects the time-dependent behavior of polarization in both electrical and mechanical spectroscopy. In dry solids, it stems from electron displacement.^73^ In wet porous media, polarization mechanisms vary with frequency, ordered from low to high as conduction, electrode, spatial, orientational, vibrational ionic, and electronic.

**Figure 8:**
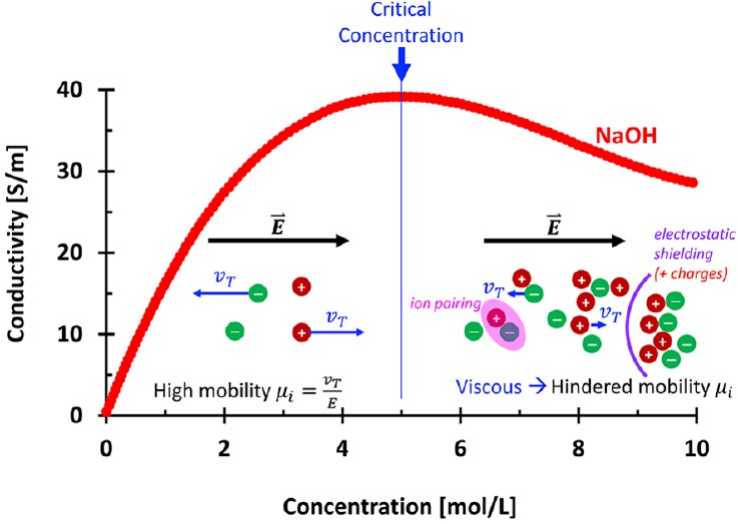
Conductivity in ionic liquids. The conductivity is determined from the summation of the ionic mobility μ_i_ for each species (cations and anions). Data source: ref 84.

### 4.2 Conduction

#### Conduction in Solids

Free electron clouds, holes (electron vacancies), and loosely bound electrons act as charge carriers contributing to conductivity and polarization in solids. In metals, the overlap of valence and conduction bands results in mobile free valence electrons, also known as free electron clouds (Figure 9). In semiconductors and insulators, electrons occupy filled bands and become mobile only when excited from the valence band to the conduction band via thermal activation or external energy^74^ (Figure 9). Semiconductors have narrower band gaps than insulators, and this gap can be further reduced by doping with impurities.

**Figure 9:**
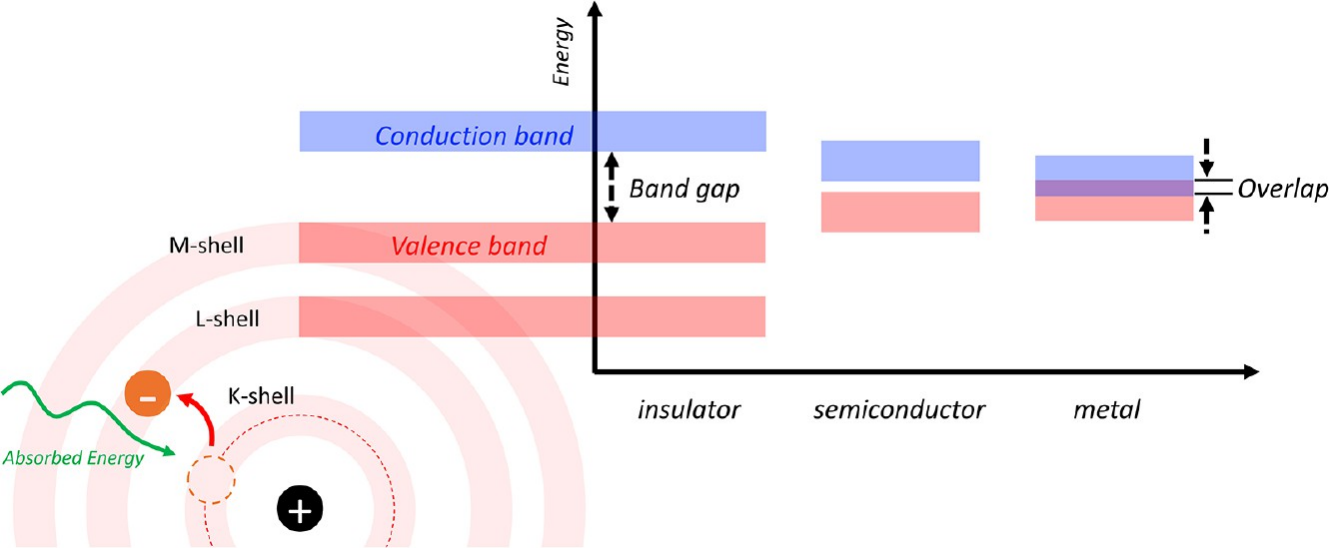
Origin of mobile electrons in solids and their energy bands: insulator (dielectric materials), semiconductor, and metal.

Pure crystalline silicon is an insulator at room temperature.^75^ Its conductivity can be significantly enhanced through the introduction of dopants, which transform it into an extrinsic semiconductor with tailored electrical properties., e.g., C-dopped Si.^76^ Conductivity of silicon-based minerals, one of the most abundant elements in geomaterials, depends on electrons and holes (electrons deprived). The Si in [Si—O—Si] is bonded by four covalent bonds and is partially broken as soon as we apply sufficient external current (analogous to reaction *R18* in Table 1). The broken bonds generate a free electron and a vacancy (a hole resulting from the removed electron), which move in opposite directions of electrical field, thereby, contribute to electrical conduction.

When dielectric materials (insulators) are polarized, electric charges do not move because there are no free electrons due to strong nuclei’s attractions. It requires a lot energy to narrow the band gap (Figure 9). However, the electron clouds shift slightly, causing low-frequency polarizations and super low conductivity (Figure 1c).

To derive the electrical conductivity of solid σ_*m*_, we expand current density ***J*** = *I*/*A* = *n*_*d*_ *e*_0_ ***v*_*e*_**. Here, *n*_*d*_ *e*_0_ together is the number of charges per unit volume [C/m^3^] and ***v*_*e*_** is the average drifting velocity of carriers [m/s]. Assuming an electron of mass *m*_*e*_ is accelerated by an electric field ***E***, Newton’s second law gives the Coulombic acceleration ***a =*** *e*_0_ ***E***/*m*_*e*_. Since collisions with atoms occur on average every $\overset{∼}{t}$ seconds (the mean free time), the average drift velocity becomes *v*_*e*_ *= a* $\overset{∼}{t}$ → *v*_*e*_ *=* *e*_0_ $\overset{∼}{t}$ ***E***/*m*_*e*_. Substituting into the expression for current density: ***J =*** *n*_*d*_ *e*_0_ ***v*_*e*_** = *n*_*d*_ *e*_0_^2^ $\overset{∼}{t}$ ***E***/*m*_*e*_. By Ohm’s law ***J =*** σ_*m*_ ***E***, we identify the electrical conductivity of solid as^69^

$$\{\begin{array}{c} \sigma_{m}=\frac{n_{d}e_{0}^{2}\overset{∼}{t}}{m_{e}}\\ \overset{∼}{t}=\frac{\overset{∼}{L}}{v_{rms}}\\ v_{rms}=\sqrt{\frac{3k_{B}T}{m_{e}}} \end{array}$$ 7

This classical model is known as the Drude model: $\sigma_{m}=\frac{n_{d}e_{0}^{2}\overset{∼}{L}}{\sqrt{3m_{e}k_{B}T}}$ where the distance $\overset{∼}{L}$ signifies the mean free-path of a particle undergoing Brownian motion with a root-mean-square (rms) velocity *v*_*rms*_. This provides a useful first approximation of conductivity in metals.^69^ Notably, the drift velocity | ***v*_*e*_** | is much smaller than *v*_*rms*_ by a factor of 10^–8^, emphasizing that electrical conduction arises from a subtle net motion superimposed on random thermal motion. Note: electron or hole masses *m*_*e*, *h*_ is 9.1093837 × 10^–31^ kg.

Drude model assumes electrons are classical particles that scatter randomly with mean free time $\overset{∼}{t}$ , moving under Newtonian mechanics without quantum effects. While it captures basic charge transport, it typically underestimates metal conductivity by about an order of magnitude due to oversimplified estimates of $\overset{∼}{t}$ using thermal velocities. In reality, electrons follow Fermi–Dirac statistics and exhibit wave behavior. Quantum models like the Sommerfeld theory replace the classical velocity *v*_*rms*_ with the faster Fermi velocity *v*_*F*_, yielding more accurate results. Further reading in ref.^69^ Practically, electrical conductivity of solid minerals σ_*m*_ is measured using $\sigma_{m}=\frac{1}{\beta Z^{*}}$ .

#### Conduction in Liquids

Electrical conduction in liquid can occur through several mechanisms:^18–20^ (i) translational ion movement resulting in net displacement across macroscopic distances between the cathode and anode, (ii) ion hopping at the molecular and atomic scales, known as Grotthuss mechanism, particularly relevant for proton transport, (iii) segmental motion, which may arise in the presence of organic molecules facilitating charge hopping within the group of molecules, and (iv) mass diffusion, or the vehicle mechanism, whereby ions migrate toward oppositely charged electrodes through bulk fluid motion.

Segmental motion refers to the movement or reorientation of parts (segments) of polymer chains or organic molecules. This motion can assist charge hopping by enabling favorable molecular conformations or bringing hopping sites closer, which enhances charge transport.^77,78^

Meanwhile, translational motion refers to the macroscale net displacement of charge carriers rather than the specific pathway of ions (Figure 1a, b). In ionic liquids, such translational displacements manifest as cations (positively charged ions) migrating toward the cathode (negatively charged electrode), while anions (negatively charged ions) migrate toward the anode (Figure 1a).

In the case of water, self-ionization results in the formation of amphoteric ions (H_3_O^+^ and OH^–^), which contribute to its electrical conductivity. Notably, proton transport in water can occur via a mechanism known as ion hopping, wherein protons (H^+^) are transferred between adjacent water molecules (Figure 1b). This process deviates from classical translational motion, as it involves localized jumps rather than continuous movement from molecule to molecule.^28^

Rather than moving as discrete ions, protons can also be transferred via successive breaking and reformation of covalent bonds within the hydrogen-bond network. This is called as a Grotthuss mechanism. This process enables rapid, directional proton mobility without the physical displacement of individual protons.^18,20,79,80^ Additionally, water dissolves CO_2_ from air at atmospheric pressure, forming H_2_CO_3_, which breaks down into H^+^, HCO_3_^–^, and CO_3_^2–^, therefore, more ions that conduct electricity.^81,82^

Ionic liquids generally show increased conductivity with rising ion concentration. However, beyond a critical concentration, further increases can lead to reduced conductivity (Figure 8). There are several mechanisms that can lower conductivity at high ionic concentrations: (i) ion pairing (less free charges^83^), (ii) increased viscosity (hindered ionic mobility^84^), and (iii) interionic interactions (electrostatic shielding^85^). We can observe this reduced conductivity in κ_eff_^″^-spectra where the spectra deflected down at low frequency (see distilled vs tap water in ref 28).

Ionic mobility μ_*i*_ is nearly constant under weak electrical fields.^86^ However, it can be field-dependent due to ion saturation effects or other complex interactions under very strong fields.^86,87^ Intrinsically, ionic mobility μ_*i*_ is governed by these physical factors:

- Size and mass: the smaller and lighter ions, easier to move and become drifted.^88,89^

- Charges and solvation: monovalent ions, e.g., H^+^, OH^–^, K^+^ have higher mobility than divalent ions, e.g., Zn^2+^, CO_3_^2–^.^90,91^ Smaller and highly charged ions like Li^+^ or Mg^2+^ tend to interact more strongly with the surrounding medium (solvent or lattice) leading to have larger hydration shells (solvation layers), subsequently reduce their mobility compared to those of larger and lower-charged ions like K^+^ or Cl^–^.^91–93^

- Viscosity: the leaner fluids allow ions to move more freely and their mobility enhanced.^92^
- Temperature: ions and molecules are always under thermal agitations. Indeed, higher temperature makes the Brownian’s motions prevails and creates thermal expansion.^70^ This causes elevated ionic mobility (thermal expansion provides less collisions) proven by increased electrolyte conductivity but not the electrical polarizability as indicated by lowered permittivity.^94,95^

At low ionic concentrations, the electrical conductivity σ_*f*_ [S/m] of ionic liquids increases approximately linearly with the number of charges and their individual ionic mobilities μ_*i*_ [m^2^·s^–1^·V^–1^]:^74,84^

$$\sigma_{f}=c_{V}F\overset{n_{I}}{\underset{j}{\sum }}z_{j}c_{i,j}\mu_{i,j}\to \sigma_{f}=c_{V}F\left(z_{a}c_{i,a}\mu_{i}^{-}+z_{c}c_{i,c}\mu_{i}^{+}\right)$$ 8

as a total contribution from the summation of *n*_*I*_ number of ionic species’ concentrations *c*_*i*_ [mol/L] amplified by each charge valences *z* [ ], where *F* is the Faraday constant 96,475 C/mol, also equivalent with the charges of each mole, *F* = *e*_0_ *N*_*A*_ (elemental charge per particle *e*_0_ = 1.602 × 10^–19^ C multiplied by the Avogadro’s number *N*_*A*_ = 6.02 × 10^23^ particles per mole). Notes: subscripts {*a*, *c*} or superscripts { – , + } for anion and cation, 1 S/m = 1 C/(m·s·V), and a volumetric unit converter *c*_*V*_ = 1000 L/m^3^.

#### Conduction in Porous Media

Mechanism of charge transport inside pores: (i) ion hopping on hydrated mineral surfaces, (ii) **drift**, driven by an external electric field and described by Ohm’s law; or (iii) **diffusion**, governed by concentration gradients per Fick’s law and independent of the field. Drift dominates in the bulk electrolyte, while diffusion is significant near interfaces, especially within counterion clouds. Both drift and diffusion often **coexist**.^96,97^

At the molecular level, the Grotthuss mechanism-alike is found in hydrated minerals. This is a movement of counterions along the mineral surface through successive adsorption–desorption of ions. This **ion hopping occurs between fixed sites** (e.g., in a lattice or along a surface) and contributes to surface conductivity in porous media.^58,98–100^

The total (bulk) electrical conductivity σ_*T*_ of porous media results from three contributions: fluid conductivity σ_*f*_, solid mineral conductivity σ_*m*_, and intrinsic surface conductivity σ*^*_*S*_ due to solid–liquid interactions^27^ (Figure 6a):

$$\sigma_{T}=\sigma_{f}S_{f}\phi \frac{1}{\tau_{f}^{2}}+\sigma_{m}\left(1-\phi \right)\frac{1}{\tau_{m}^{2}}+{\mathrm{\sigma ̂}}_{S}\lambda_{D}S_{S}\rho_{m}\left(1-\phi \right)\frac{1}{\tau_{S}^{2}}$$ 9

Assuming equal tortuosities for the fluid and solid phases (τ_*m*_ = τ_*f*_ = 1), and defining a surface conduction factor Γ = σ*^*_*S*_λ_*D*_ρ_*m*_/τ_*S*_^2^, eq 9 simplifies to

$$\sigma_{T}=\sigma_{f}S_{f}\phi +\left(\sigma_{m}+\Gamma S_{S}\right)\left(1-\phi \right)$$ 10

The volumetric fluid content is given by θ_*f*_ = *S*_*f*_ϕ. Surface conduction is mainly governed by the product Γ*S*_*S*_, which combines intrinsic surface conductivity σ*^*_*S*_, the diffuse double layer λ_*D*_, and the specific surface area *S*_*S*_ (Figure 6b-i). Units of surface conductivity Γ*S*_*S*_ are [S/m], given that Γ is in [S·m^–3^·g] and *S*_*S*_ in [m^2^/g].

Mineral conductivity σ_*m*_, typically between 10^–8^ and 10^–4^ S/m, is negligible compared to saline fluid conductivities (σ_*f*_≈ 10^–3^ to 2 S/m). Thus, the simplified model becomes

$$\sigma_{T}=\sigma_{f}S_{f}\phi +\Gamma S_{S}\left(1-\phi \right)$$ 11

In clean, clay-free media, the first term dominates, aligning with Archie’s equation:^101^

$$\sigma_{T}=\sigma_{f}S_{f}\frac{\phi }{\tau_{f}^{2}}$$ 12

To fit experimental data, the equation is generalized using positive constants *m* and *n*:

$$\sigma_{T}=\sigma_{f}S_{f}^{n}\frac{\phi^{m}}{a}$$ 13

Here, *a* = τ_*f*_^2^ represents the tortuosity factor. For fully saturated conditions (*S*_*f*_ = 1), the **formation factor** *F* (also known as the resistivity factor) is defined as

$$F=\frac{\sigma_{f}}{\sigma_{T,1}}=\frac{\rho_{T,1}}{\rho_{f}}\to \frac{1}{F}=\frac{\phi^{m}}{a}$$ 14

This formation factor *F* is widely used in petroleum industry to quantify the conductivity σ_*T*,1_ or resistivity ρ_*T*,1_ of saturated rocks. The resistivity index *I* = σ_*T*,1_/σ_*T*_ = ρ_*T*_/ρ_*T*,1_ compares the saturated properties to that at arbitrary saturation.

### 4.3 Electrode Polarization

#### Definition

Electrode polarization occurs within the conduction regime at low frequencies (*f* < 100 kHz) and arises from the accumulation of charges at electrode–electrolyte interfaces due to differing charge carriers (Figure 10). Electron clouds in solid electrodes cannot transfer into the electrolyte, nor can free ions migrate into the solid, leading to interfacial charge buildup known as the electrical double layer.

**Figure 10:**
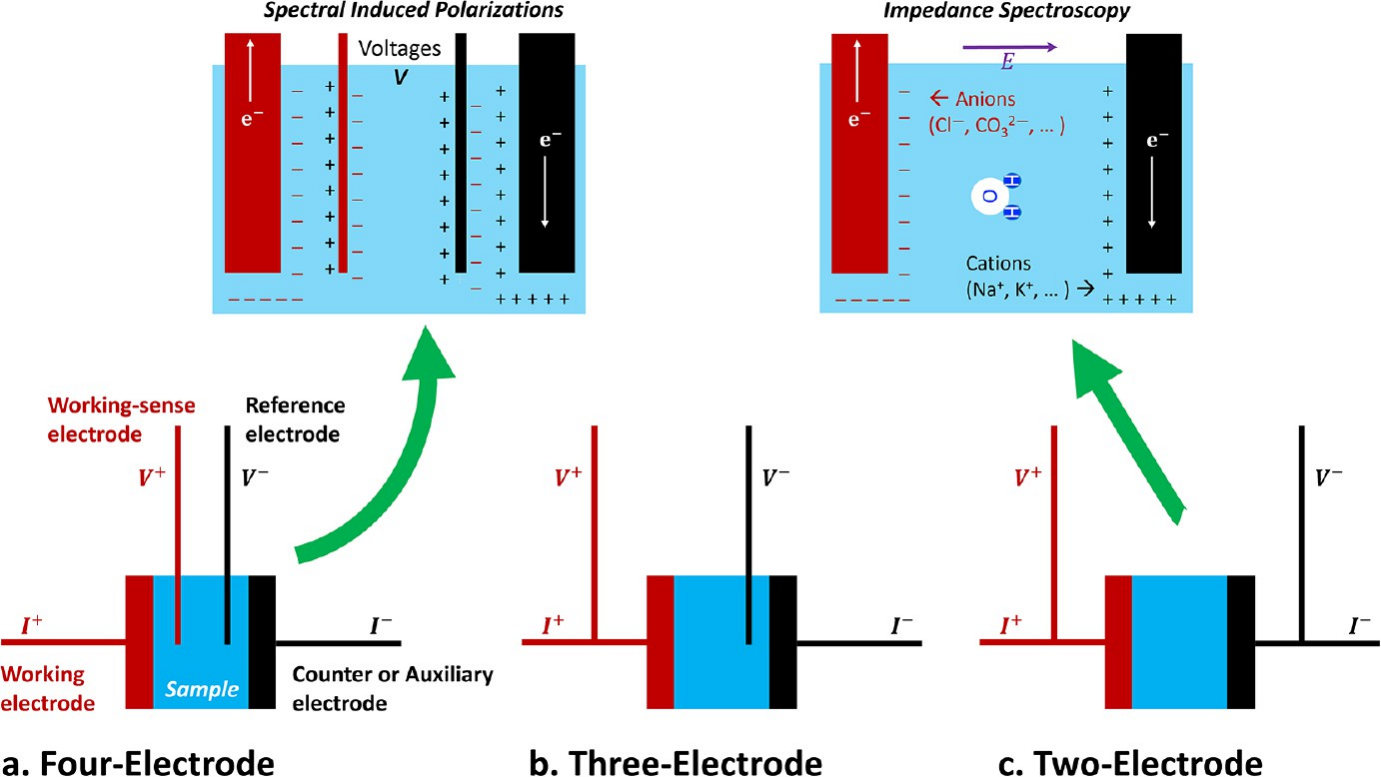
Electrode-based conductivity measurement systems: (a) four, (b) three, and (c) two terminals. The term *terminal* is equivalent to the *electrode*. A relative separation of ions remains evident in the vicinity of the electrodes yet it is electrically neutral in the bulk region. Geoscience terminology: the four-electrode method is known spectral induced polarization (SIP), the two-electrode method refers to impedance spectroscopy. While three-electrode method is not popular in geoscience, yet, in chemistry, catalyst, and battery communities, the three-electrode configuration is named for electrochemical impedance spectroscopy (EIS).

These nano- to micron-scale layers can be modeled as capacitors or constant phase element^102^ or a parallel circuit between resistor and constant phase element.^37^ Electrode polarization is observable in the quadrature conductivity σ″^103^ and more prominently in the extremely high permittivity κ^′^.^104,105^

Electrochemical systems conserve both charge and mass. In the absence of Faradaic reactions, this conservation gives rise to non-Faradaic processes, such as capacitive charge accumulation at the electrode–electrolyte interface without any net charge transfer. Free ions and counterions within the pore fluid and wet porous media serve as mobile charge carriers, and their presence does not depend on the specific electrode configuration. Consequently, even well-designed electrode setups cannot fully prevent charge buildup at the electrode–sample interfaces.

#### What Influences Electrode Polarization?

Electrode polarization effects are more pronounced in conductive materials due to increased ionic accumulation at the interface.^28^ Increased temperature enhances ionic mobility, subsequently conductivity, hereby, intensifying electrode polarization (higher permittivity κ^′^ at low frequency) due to faster charge accumulation at interfaces. A faster characteristic time (shorter time) corresponds to a higher electrode polarization limiting frequency *f*_*EP*_.^106^

Electrode–sample contact quality such as poor contact (e.g., air gaps, rough surfaces) can lead to nonuniform field distribution, enhancing polarization artifacts.^107^ Blocking electrodes (nonreactive, no charge transfer) cause stronger polarization than nonblocking (electrochemically active) electrodes.^107^ Thicker samples increase the distance over which ions migrate, amplifying polarization effects.^108^ Probe geometry also affects **field uniformity**, influencing how polarization builds at boundaries.^109,110^

Electrode polarization is also significantly influenced by the choice of electrode material. For instance, in KCl electrolytes, the extent of electrode polarization increases in the order: aluminum, nickel, gold, and platinum.^111^ Additional experiments using various metal–electrolyte combinations show differing polarization behaviors, such as lower polarization for platinized platinum with AgCl, and higher polarization in systems involving stainless steel in NaCl solutions.^112^ Furthermore, corrosion of anodic materials can compromise measurement stability. In this context, carbon-based materials, particularly graphite, are favorable due to their chemical inertness and more positive corrosion potential compared to reactive metals like aluminum.^28^

#### How to Mitigate Electrode Polarization?

The four-electrode configuration, commonly known as spectral induced polarization (SIP), effectively mitigates electrode polarization effects that are prevalent in two-electrode systems (impedance spectroscopy). Four-electrode configuration does not alter the ionic distribution at the current electrodes, it minimizes the effect of electrode polarization on the measured signal by decoupling current injection from potential measurement. This results in reduced real permittivity κ^′^ and a lower limiting frequency of electrode polarization *f*_*EP*_, allowing for more reliable measurements at low frequencies

The characteristic frequency of electrode polarization *f*_*EP*_ is lower in four-electrode configurations, expanding the usable frequency range for SIP measurements.^113–115^ Some researchers argue that extremely high permittivity κ^′^ values result from electrical double layer and Stern layer polarizations^116–118^ in high-specific surface materials or measurement limitations due to phase resolution thresholds, typically around 0.1 mrad.^115^

However, at higher frequencies, the four-electrode method may introduce additional challenges such as inductive electromagnetic coupling^119^ and parasitic capacitive coupling (PCC).^115^ These problems are not observable in the two-electrode method.

PCC refers to unintended capacitance within amplifier lines, acting parallel to the voltmeter, and results in current leakage. Unlike electrode polarization capacitors, which appear in series with the voltmeter, PCC components arise from system architecture.^115,120,121^ Even after correcting for PCC, signatures of electrode polarization may persist, such as the increase of real permittivity κ^′^ with decreasing frequency (Figure 11).

**Figure 11:**
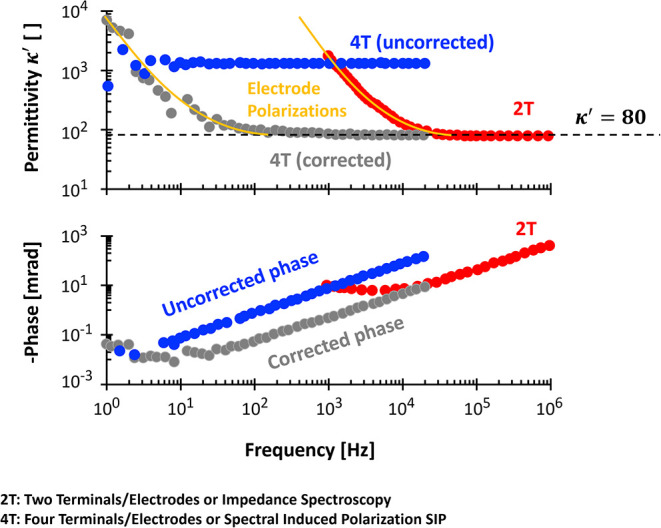
Spectra from two- and four-terminal methods. Data source of 0.01 S/m NaCl: ref 115 and personal communications with Dr. Chen Wang.

### 4.4 Spatial Polarization

#### Nomenclature

Space charging polarization occurs at frequencies higher than those dominated by conduction or electrode effects and lower than dipole polarization (Figure 12). It arises from the accumulation or distribution of charges within the material, often termed ″spatial polarizations″. Other names for spatial polarization include space charge, membrane, Stern layer, diffuse double layer, localized ion hopping, Maxwell–Wagner, interfacial, and bound-water polarizations, and sometimes α- and β- relaxations.

**Figure 12:**
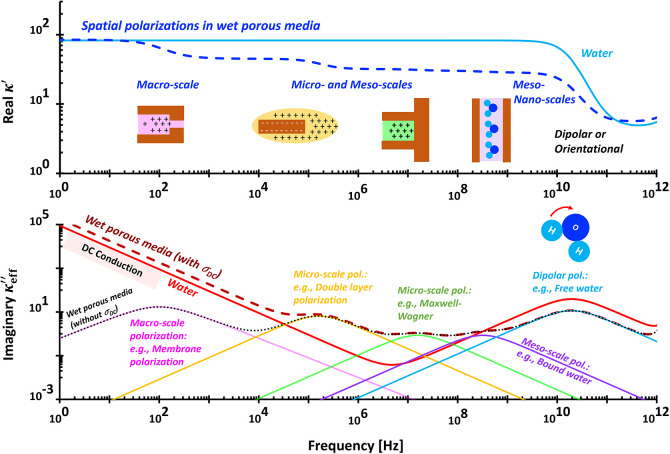
Idealized spatial polarizations. Parameters used in each polarization: 10^–5^ S/m (conductivity), *κ*_U_ = 40, *κ*_L_ = 0, τ = 1.59 × 10^–2^ s, δ = 0.9 (macro-scale, e.g., membrane polarization), *κ*_U_ = 15, *κ*_L_ = 2, τ = 10^–6^ s, δ = 1 (microscale, e.g., double layer polarization), *κ*_U_ = 1.7, *κ*_L_ = 0, τ = 10^–8^ s, δ = 1 (microscale, e.g., Maxwell–Wagner polarization), *κ*_U_ = 1.7, *κ*_L_ = 0, τ = 5 × 10^–10^ s, δ = 1 (mesoscale, e.g., bound water polarization), and *κ*_U_ = 24, *κ*_L_ = 0.9, τ = 8.2 × 10^–2^ s, δ = 1 (orientational polarization). See a comparison with ref 28.

The term “*interfacial polarization”*, as used here, refers to polarization arising from material heterogeneities such as between distinct phases, grain boundaries, or different constituents within a composite medium; and should not be confused with *“electrode polarization”*, which occurs at the interface between the material and the measuring electrode probes. The term interfacial polarization can be used interchangeably with Maxwell–Wagner polarization.

In geosciences, α- and β-polarizations often refer to polarized counterions in diffuse double layer (*f* < 10 kHz) and Maxwell–Wagner or interfacial polarizations (10 kHz<*f* < 10 MHz).^118^ In physical chemistry, α- and β-polarizations describe full structural^122^ and local rearrangements^123^ relaxations of entire molecules or molecular segments in supercooled liquids, with β also known as Johari–Goldstein relaxation. In both fields, α-polarization occurs at lower frequencies than β.

Bound-water polarization can be classified into two categories either **spatial** or **orientational** polarization depending on the aspect under consideration. The migration of bound water along mineral surfaces contributes to **spatial polarization**, while the **reorientation of bound-water dipoles** under an external electric field constitutes **orientational polarization**, which will be discussed in detail later.

#### Spatial Polarization in Liquids

Low-viscosity liquids like water at room temperature do not exhibit spatial polarization of either their transient proton-transfer dynamics (as in the Grotthuss mechanism) or their permanent molecular dipoles. Rapid molecular motion and high diffusivity leads to behavior resembling DC conduction, which prevents the buildup of long-range spatial polarization.

In contrast, high-viscosity liquids, such as polymers,^124^ supercooled liquids,^125^ metallic glasses,^126,127^ and glassy materials^128,129^ , resist spatial motion, enabling spatially localized processes like segmental reorientation, atomic rearrangement, or molecular reorientation. These are often described as motion within a “cage” of neighboring molecules.^129^

#### Spatial Polarization in Porous Media

Figure 12’s inset illustrates spatial polarization mechanisms in porous media, driven by free ions and counterions. The characteristic length associated with spatial polarization mechanisms is inversely proportional to frequency. At low frequencies (Hz–kHz), macro-scale processes such as membrane polarization are predominant. Intermediate frequencies (kHz–MHz) correspond to microscale mechanisms, including Stern layer polarization, diffuse double layer relaxation, ion hopping or Grotthuss-like proton transfer, and Maxwell–Wagner polarization. At higher frequencies (MHz to sub-GHz), mesoscale processes such as bound water orientational polarization become increasingly significant.

Membrane polarization is a specific form of Maxwell–Wagner polarization that arises in porous media with constricted pores, which act as ion-selective barriers^130^ (Figure 13a-i). In such cases, free ions accumulate at pore throat, leading to enhanced polarization and reduced effective conductivity. These characteristics are often associated with nearly closed pores and ineffective porosity. As pore sizes increase, ion mobility improves, and the associated polarization process shifts toward lower frequencies in the permittivity spectrum.

**Figure 13:**
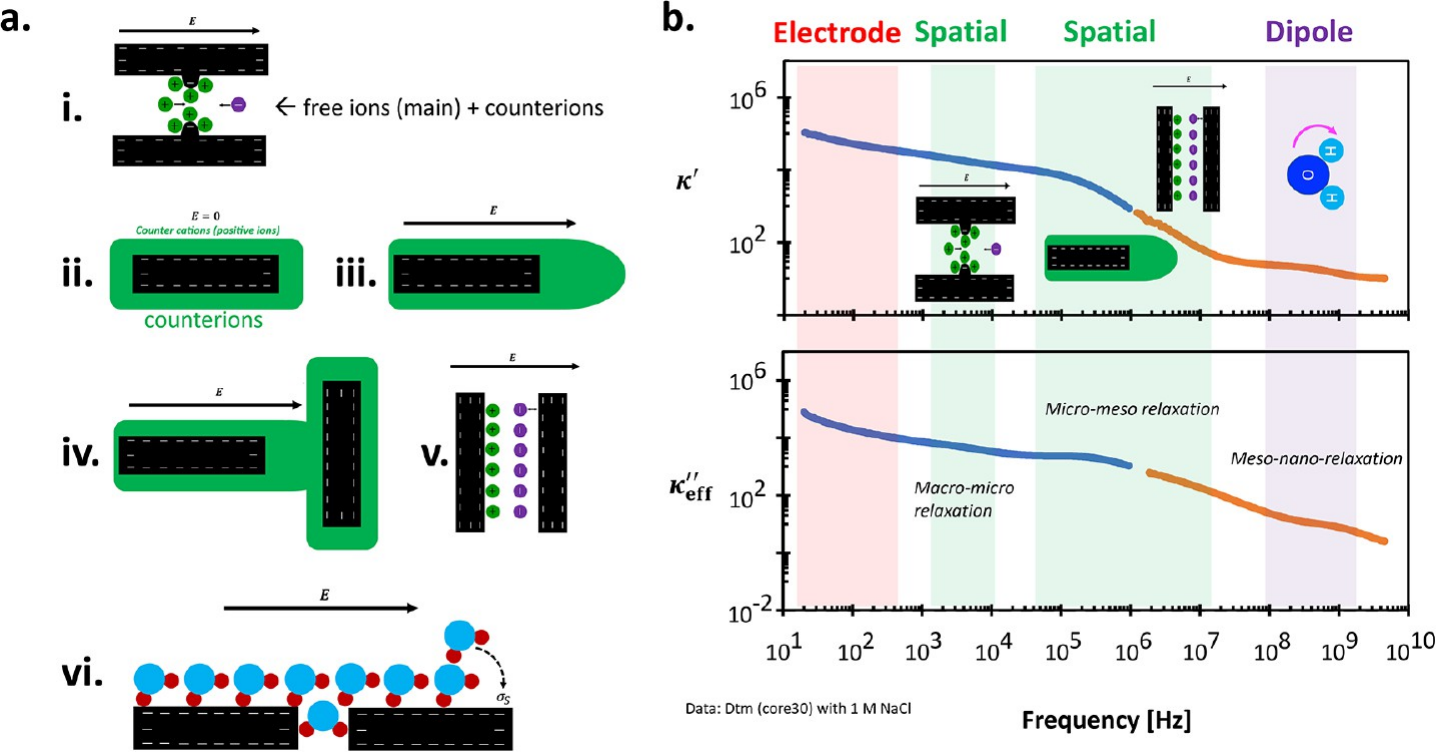
Spatial polarizations. (a) Physical observations of spatial polarizations at the macro-to-nano scale; (b) Complex permittivity spectra of typical porous media with multiple spatial polarizations. Blue data is measured with LCR meter (20 Hz to 1 MHz) and orange data is measured with vector network analyzer (1 MHz to 4.5 GHz).

When there is no applied electric field, wet grain surfaces are evenly surrounded by counterions within the electrical double layer (Figure 13a-ii). Under thermal activation, localized ion hopping between fixed surface sites such as on a lattice or mineral surface can contribute to both ionic conduction and interfacial polarization. When this process involves proton transfer through hydrogen-bonded networks, it is referred to as Grotthuss-like proton transfer.^98–100^

Counterions can contribute to polarization when located near grain edges (Figure 13a-iii), at mineral–mineral interfaces (Figure 13a-iv), or at surfaces oriented perpendicular to the applied electric field (Figure 13a-v), where charge displacement is spatially constrained. Maxwell–Wagner polarization at misaligned surfaces can arise from the combined effects of counterions in the electrical double layer and trapped free ions within the pore structure (Figure 13a-v).

However, pronounced surface conduction, enabled by mobile ions along the mineral surfaces, can facilitate charge transport that diminishes the buildup of interfacial polarization (Figure 13a-vi). Thus, surface conduction and polarization often act in competition within the spatial polarization regime. A comparison between systems with negligible and significant conduction reveals that elevated conduction can mask or diminish the expression of macro-scale polarization mechanisms (Figure 12).

The extremely high permittivity values κ′, observed within the 20 Hz–1 MHz range as shown in Figure 13b, are often attributed to α-polarization mechanisms such as charge accumulation in the Stern or diffuse layers of soil grains,^118,131^ or even microbial membranes.^132^ However, permittivity values exceeding that of free water by up to 6 orders of magnitude raise skepticism. In such contexts, it is unlikely that bound or confined water could contribute significantly, as its polarizability is restricted by mineral surfaces and pore confinement. Consequently, several researchers argue that these extreme κ′ values likely result from **electrode polarization artifacts**, even when using four-electrode configurations.^107,113,114^

#### Spatial Polarization in Insulators

Polarization in insulators or dielectric materials primarily arises from electronic polarization, in which the electron cloud surrounding atoms or molecules shifts slightly under an applied electric field (Figure 1c). This mechanism involves no net movement of charges and occurs in materials with bound charges. We will explore this in more detail in the *Electronic Polarization* section. Because this behavior is typical of dielectric materials, it is also commonly referred to as dielectric polarization.

In heterogeneous insulators, however, additional polarization effects can occur due to structural inhomogeneities. For instance, the presence of trapped nanosized polar impurities and compositional interfaces can lead to interfacial or Maxwell–Wagner polarization.^128^ In such systems, phase boundaries act as charge accumulation sites, resulting in spatial charge separation across microscopic regions.^133–136^ Therefore, in certain cases, dielectric polarization may also include a spatial component arising from these interfacial effects.

### 4.5 Orientational Polarization

#### Nomenclature

The term *orientational* refers to the alignment of dipolar molecules or charged colloidal particles within a fluid in response to an external electric field. Orientational polarization, also called *dipolar polarization*, arises when these permanent dipoles rotate to align with the field direction. For instance, in a water molecule, the oxygen atom carries a partial negative charge, while the two hydrogen atoms contribute to a partial positive charge, resulting in a net charge *q* and dipole moment **μ** *_E_* (Figure 14a). When an electric field ***E*** is applied, it exerts torques on these dipoles, causing them to rotate. The torque experienced by a dipole is given by **τ** = **μ** *_E_* × ***E***, where **μ**_*E*_ = *q* ***d*** is the dipole moment. This torque is responsible for the reorientation of dipoles in the field and is governed both by the molecular dipole strength and the external field magnitude.

**Figure 14:**
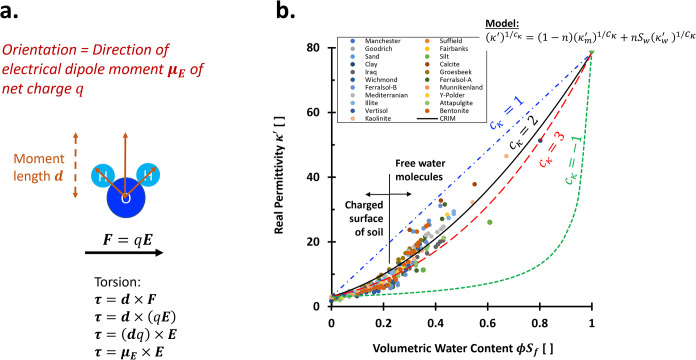
Orientational polarizations. (a) Definition of electric moment **μ**_**E**_ which controls the torsion **τ** related to oriental polarizations; (b) Selected real relative permittivity at 1–1.5 GHz for various volumetric water content. Data sources: refs 355–360 . In geotechnical engineering, porosity is written as *n*; while in geophysics and petroleum engineering, porosity is expressed as ϕ.

#### Orientational Polarization in Liquids

The permittivity of bulk water (κ^′^ ≈ 80 at room temperature) reflects orientational polarization constrained by hydrogen bonding. This polarization dominates between 0.01–30 GHz, peaking near 18 GHz, and is described by the Cole–Cole model (Figure 4). At low frequencies, electrode polarization limits measurements.

Many water–organic mixtures and low-molecular weight glass-forming liquids exhibit asymmetric permittivity dispersion. This is typically manifested as a high-frequency relaxation tail known as the ***excess wing***, which extends beyond the characteristic frequency *f*_*c*_ ^137,138^ and is often attributed to a submerged β-relaxation process.^139^ Studies on ethanol,^140^ propylene carbonate and glycerol,^141^ and broader reviews^142^ document this behavior in detail. This phenomenon is commonly attributed to relaxation within hydrogen-bonded polar molecular networks, e.g., internal motions or side-chain dynamics,^37,143,144^ which generate a secondary process overlapping the primary Debye peak at the *f*_*c*_. The resulting asymmetric broadening can be effectively modeled using Cole-Davidson or Havriliak–Negami models.^145^

Orientational polarization is characterized by a critical frequency *f*_*c*_, whose inverse defines the dielectric relaxation time τ = (2πf_*c*_)^−1^, as described in the Cole–Cole model.^28^ This relaxation time represents how quickly a dipole returns to equilibrium after polarization. In saline solutions, water’s orientational response is hindered by dissolved ions, leading to lower permittivity and shorter τ. As salt concentration increases, these relaxation times decrease due to stronger ion-dipole interactions. The Debye–Stokes model relates the relaxation time τ to fluid viscosity η, effective molecular radius *r*_0_ and temperature *T* such that τ = 4πη*r*_*o*_^3^/(*k*_*B*_ *T*).^70,146^

#### Orientational Polarization in Porous Media

In geoscience, this primary dipolar process or orientational polarization is termed γ-polarization.^118^ Inside porous media, the polarizability of water molecules is hindered due to the localized fields exerted by both surface charges and free ions (Figure 6). The orientational permittivity κ^′^ of free water in saturated porous media simply corresponds to the volumetric water content θ_*w*_ within confined porous media with a porosity ϕ and is often approached by κ^′^ ≈ θ_*w*_κ_*w*_^′^ ^26^ (simplified mixing model). Hence, the water permittivity around 1 GHz is beneficial for predicting the degree of saturation *S*_*w*_ or porosity ϕ, such that θ_*w*_ = ϕ*S*_*w*_ (Figure 14b). A confined nanoscale space can hinder water polarizability significantly down to its electronic permittivity κ^′^∼2.^147^

In low water content systems, bound-water polarization becomes dominant as free bulk water is absent. This polarization, associated with water adsorbed on mineral surfaces, typically occurs in the MHz range, lower than bulk water’s GHz-range orientational polarization but higher than spatial polarizations.^148,149^

In materials with both micro- and nanopores, such as clays or cement, both bound and bulk-like orientational polarizations may coexist. However, bound-water effects are difficult to distinguish in large-pore or high-salinity systems due to overlapping signals from spatial and electrode polarizations.^150^ While fundamentally dipolar, bound-water polarization often exhibits spatial characteristics due to its confinement near solid surfaces.

### 4.6 Vibrational Ionic Polarizations

#### Classical vs Vibrational Ionic Polarizations

Classical ionic polarization refers to the displacement of positive and negative ions in a crystal lattice of ionic solids under the influence of an external electric field. This displacement generates an electric dipole moment within the material, contributing to its overall polarization. This effect is usually **slow** (kHz to MHz) because ions are heavy and move sluggishly.^151^ At higher frequencies, ions cannot physically move fast enough to keep up with the field oscillations, so classical ionic polarization diminishes.^152^

Vibrational ionic polarizations occur within THz frequencies (∼0.1–10 THz or 10^11^ to 10^13^ Hz) and correspond to very fast oscillations, in the range of picoseconds. At these frequencies, bulk ionic displacement (classical ionic polarization) is generally too slow to respond. However, what can be observed at THz frequencies: (i) Phonon-related collective lattice vibrations in ionic crystals,^153^ (ii) Localized vibrational modes of ions in complex molecules or solids,^154^ and (iii) Hydration shell dynamics in solutions.^152^

In ionic crystals like NaCl, optical phonons involving ionic vibrations show absorption peaks in the THz/far-IR range.^155^ In liquids, ionic motions are overdamped and do not produce sharp resonances, but the coupling of ions and solvent can cause broad THz absorption features.^152,156^ These fast ionic vibrational modes contribute to the complex permittivity response at THz frequencies, often modeled as part of vibrational ionic polarization.^152^ We will expand the vibrational ionic polarization to be intermolecular and intramolecular polarizations in the following section.

#### Type of Motions

Intermolecular polarizations focus on the oscillating intermolecular forces (e.g., H-bonds) among water molecules when they are exposed to an external electrical field at an infrared bandwidth of approximately 300 GHz-430 THz.^152,157^ These include the bending, stretching, and libration motions (Figure 15a-i).

**Figure 15:**
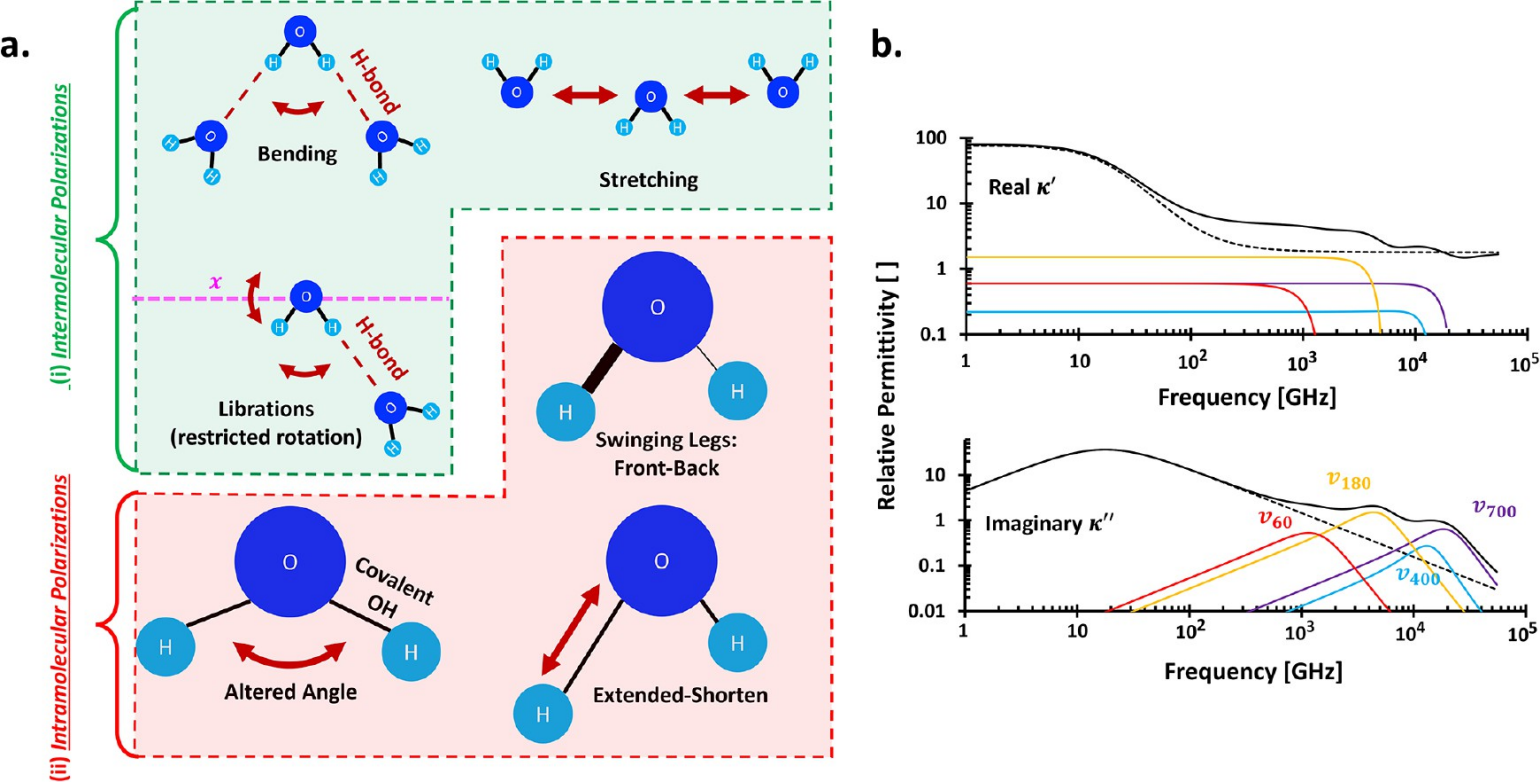
Vibrational ionic polarizations. (a) Physical observations of intermolecular and intramolecular polarizations; (b) Complex permittivity spectra at frequencies higher than 1 GHz. Plot is developed with the extended Cole–Cole equation, parameters: κ_∞_ = (4/3)^2^, σ_DC_ = 10^–4^ S/m, Δκ = 75.3, τ_def_ = 12.4 ps, τ_osc_ = 35 ps, δ = 0.93, A_60_ = 0.6, ω_60_ = 9 THz, Γ_L,60_ = 1.13 × 10^13^, A_180_ = 1.5, ω_180_ = 32 THz, Γ_L,180_ = 3.39292 × 10^13^, A_400_ = 0.22, ω_400_ = 90 THz, Γ_L,400_ = 7.54 x10^13^, A_700_ = 0.6, ω_700_ = 132 THz, Γ_L,700_ = 1.32 × 10^14^. Note that the summation of κ_∞_, Δκ, A_60_, A_180_, A_400_, and A_700_ is 79.9978. Dashed line is the Debye’s trend: only involves the orientational polarization with parameters: κ_∞_ = (4/3)^2^, σ_DC_ = 10^–4^ S/m, Δκ = 75.3, τ_def_ = 12.4 ps, τ_osc_ = 35 ps, δ=0.93.

Libration is a type of restricted or hindered rotational motion that occurs when a molecule (or part of it) attempts to rotate but is constrained by its environment, for example, due to hydrogen bonding or interactions with neighboring molecules.

Meanwhile, intramolecular polarizations signify the polarized constituting ions or atoms within the internal molecule bodies and are observed at much higher frequencies than intermolecular polarizations.^157–159^

Intramolecular polarizations in water molecules are marked by covalent OH bond alterations (Figure 15a-ii), such as (i) front-and-back swinging legs, (ii) altered angles acting like a scissor, and (iii) extending shortening. In larger organic molecules, ionic polarization helps identify the possible existing bonds, which further assists in liquid characterizations, e.g., the use of Fourier transform infrared.^160^

#### Permittivity for Vibrational Spectra

We can compute the complex relative permittivity κ* spectrum from frequency-dependent values of the optical refractive index *n*, absorption coefficient α, and the wave extinction coefficient η, derived from Fourier transform infrared, Raman, or THz spectra:^161,162^

$$\{\begin{array}{c} n=\frac{c\Phi }{\omega d_{s}}+1\\ \alpha =\frac{2}{d_{s}}\ln\left[\frac{4n}{Â{\left(1+n\right)}^{2}}\right]\\ \eta =\frac{c\alpha }{2\omega }\\ \kappa^{*}=\kappa^{\prime }-j\kappa^{\prime \prime }=\left(n^{2}-\eta^{2}\right)-j2n\eta \end{array}$$ 15

where the Φ is the phase difference [rad], *Â* is the amplitude ratio [ ], *d*_*s*_ is the sample thickness [m], and *c* is the speed of light.

Equation 15 provides experimental vibrational complex permittivity that can be fitted by the extended Cole–Cole equation:^152^

$$\kappa_{\mathrm{eff}}^{*}=-j\frac{\sigma_{DC}}{\omega \varepsilon_{0}}+\kappa_{\infty }+\frac{\Delta \kappa }{1+{\left[\frac{1}{j\omega \tau_{\mathrm{def}}}+\frac{1}{{\left(j\omega \tau_{\mathrm{osc}}\right)}^{\delta }}\right]}^{-1}}+\overset{N}{\underset{i=n}{\sum }}\left(\frac{{Ȧ}_{i}\omega_{i}^{2}}{\omega_{i}^{2}-\omega^{2}+j\omega \Gamma_{L_{i}}}\right)$$ 16

The subscript *i* denotes unique polarizations observed within a molecule at certain wave numbers $\overset{∼}{v}$ , polarization frequency ω_*i*_ = 2π*c* ${\overset{∼}{v}}_{i}$ , resonance amplitude Ȧ , and Lorentzian damping constant Γ_L_. Water molecules show intermolecular polarizations with the following wave numbers $\overset{∼}{v}$ [cm^–1^]: 60 for bending motions between neighboring water molecules in acute angles, 180 for stretching motions between neighboring water molecules in a planar mode, 400 and 700 for both libration motions observed within different rotating axes.^152^

Figure 15b shows the intermolecular polarizations plotted using eq 16. Let us compare eq 16 with the original Cole–Cole model (eq 2 with the β = 1). We equate the following variables: ultrahigh frequency permittivity κ_∞_ = κ_*L*_, Δκ = κ_*U*_ – κ_*L*_ = κ_*s*_ – κ_∞_, and a general relaxation time τ term extends to the defect τ_def_ and oscillation time τ_*osc*_ in this relationship $\frac{1}{{\left(j\omega \tau \right)}^{\delta }}=\frac{1}{j\omega \tau_{\mathrm{def}}}+\frac{1}{{\left(j\omega \tau_{\mathrm{osc}}\right)}^{\delta }}$ . These effects associate with the intermolecular polarizations. The latest term in eq 16 that includes Lorentzian damping constant Γ_L_ designates the intramolecular polarizations.

### 4.7 Electronic Polarization

#### Low-Frequency Electronic Polarization

Electronic polarization commonly referred to as dielectric polarization in dielectric materials does not involve the migration of charges or the occupation of space by free carriers. Instead, it arises from subtle displacements in electron density, resulting in induced dipole moments. This phenomenon typically manifests across a broad frequency spectrum, ranging from hertz to terahertz (Figure 1c, read ref 73).

Such behavior is characteristic of dry, electrically nonconductive solids, generally classified as dielectrics or insulators.^163^ A perfect dielectric has negligible conductivity and supports only slight shifts in bound electrons, allowing energy storage akin to an ideal capacitor.^164^ Nonporous dielectrics do not generally pose orientational (Figure 14) or ionic polarizations (Figure 15), as seen in materials like hexagonal boron nitride.^165^

In contrast, certain dry porous media such as minerals, soils, rocks and woods may exhibit additional dispersive permittivity behavior due to interfacial or structural heterogeneities, even in the absence of free fluids. Other materials like rubber and glass primarily display intrinsic dielectric responses dominated by electronic polarization.

This response is largely constant across frequencies, defining an upper limit denoted as ε_*U*_*EP*__^′^, also referred to as the dielectric constant (Figure 16a). This value closely corresponds to the static permittivity κ_*s*_, often used interchangeably in practice, e.g., 78 for water, 24 for ethanol, and 1 for air.

**Figure 16:**
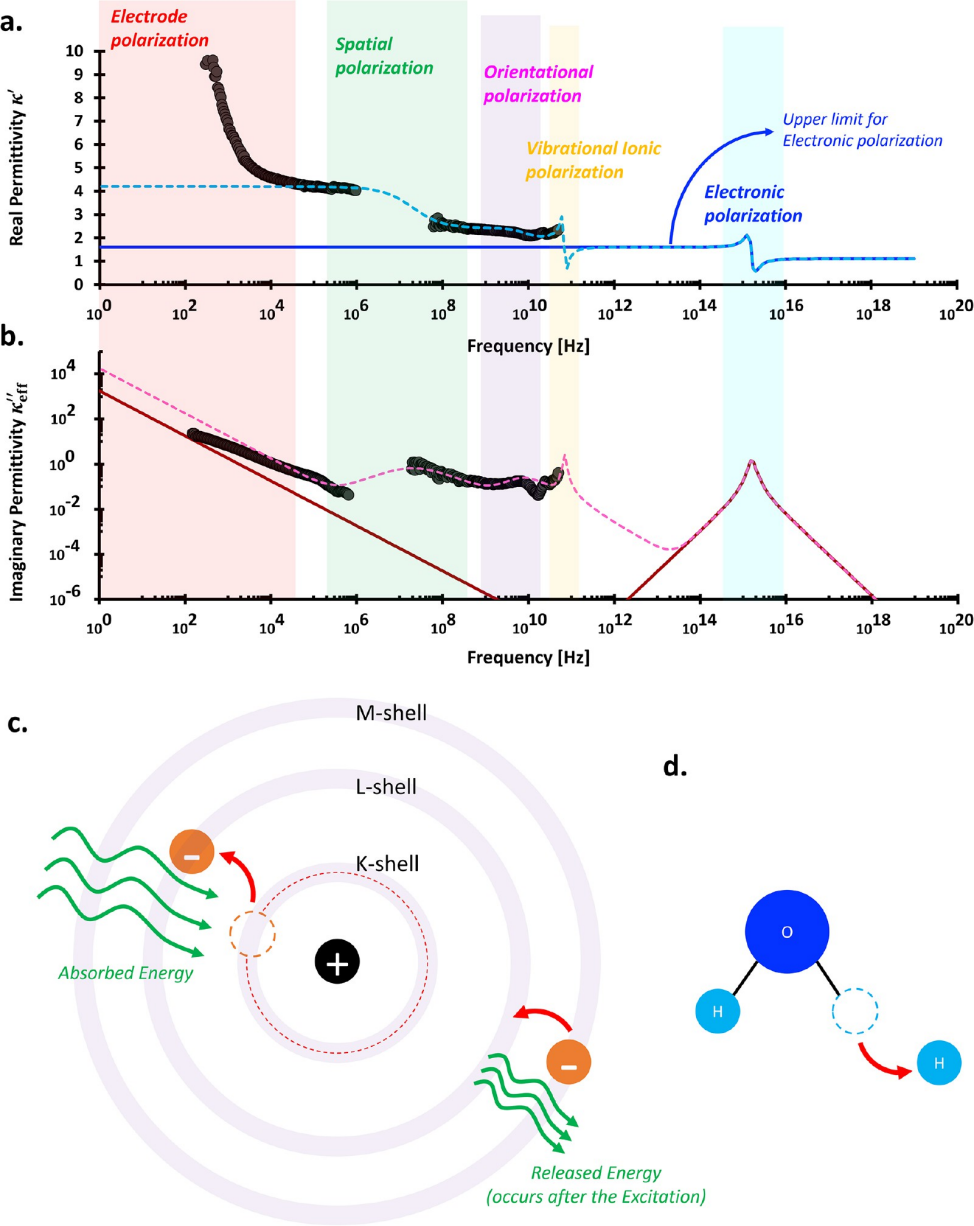
Electronic polarizations and excitations. (a) Real permittivity and (b) imaginary permittivity for dry rocks with only electronic polarizations (κ′: dark blue; κ_eff_^″^: maroon lines) vs with a minute moisture content from atmosphere (κ′: light blue dash; κ_eff_^″^: pink dash lines); (c) Excitation of an electron to a higher energy state (outer shell); (d) expulsion of hydrogen from a water molecule. Energy absorptions and release do not occur at the same time. Plots in (a, b) are the Cole–Cole model with following parameters for rocks with only electronic polarization: conduction: σ_DC_ = 1 × 10^–7^ S/m; electronic polarization: κ_U_^′^ = 1.6, κ_L_^′^ = 1.1, τ = 1 × 10^–16^ s, δ = 1.8; for rocks with a minute moisture content: conduction: σ_DC_ = 1 × 10^–6^ S/m, spatial polarization: κ_H_^′^ = 1.8, τ = 7.96 × 10^–16^ s, δ = 0.8, orientational polarization: κ_H_^′^ = 0.4, τ = 2 × 10^–11^ s, δ = 1.1, vibrational ionic polarization: κ_H_^′^= 0.5, κ_L_^′^ = 0.1, τ = 2.3 × 10^–12^ s, δ = 1.9, and electronic polarization: κ_H_^′^= 1.5, κ_L_^′^= 1, τ = 1 × 10^–16^ s, δ = 1.8.

#### High-Frequency Electronic Polarization

At high frequencies (above ∼ 10^14^ Hz), electronic polarization dominates, involving the slight displacement of bound electrons relative to atomic nuclei under an external field. As frequencies approach the ultraviolet regime (750 THz–30 PHz), photons may excite electrons to higher energy levels, and at sufficiently high energies, photoionization can occur. These processes go beyond dielectric polarization and involve **electronic excitation** and bond disruption.

The Bohr model may offer a conceptual reference for discrete energy transitions in hydrogen^166^ (Figure 16c and d), though it is not suitable for describing polarization in bulk materials. Once only electronic polarization remains, the real part of permittivity κ^′^ approaches its lower bound (e.g., κ^′^∼1.3–1.8 for highly confined water molecules^147,167^ ). In practice, dry porous materials often absorb atmospheric moisture (Figure 16a and b), enabling various polarization mechanisms including orientational, ionic, and interfacial alongside minimal conduction.^168^

## 5 Measurements

Electrical property measurement techniques are generally categorized into two main groups: **electrode-based methods** and **electromagnetic wave propagation-based methods**. Electrode-based approaches are applicable when the physical length of the sample *L* is significantly smaller than the corresponding wavelength λ, such that, *L*/λ ≪ 1. Under this condition, lumped-element circuit models are appropriate for interpreting the measurements, and instruments such as LCR meters are typically employed.

Conversely, when the sample dimension approaches or exceeds the electromagnetic wavelength (*L*/λ ≫ 1), wave propagation effects dominate, necessitating the use of distributed parameter models. For samples in the millimeter-to-centimeter range, high-frequency techniques operating between approximately 1 MHz and 10 GHz are required. These are often implemented using instruments such as vector network analyzers^26^ or time domain reflectometry (TDR).^169^ In contrast, for field-scale applications involving meter- to kilometer-scale samples, low-frequency measurements (typically in the range of 1 mHz to 100 kHz) are more appropriate (Figure 2). Recent studies have demonstrated the use of a unified probe configuration capable of operating across this broad frequency range through the integration of LCR meters and TDR instruments.^28^

With increasing frequency, the electromagnetic wave characteristics, namely the wavelength λ and skin depth *S*_*d*_, tend to decrease, while the phase velocity *v*_Φ_ typically increases (Figure 17). These behaviors are strongly influenced by the material’s **effective complex permittivity** κ_eff_^*^, which in turn affects wave propagation. The relevant formulations are as follows:^70^

{λ=cfIm[−κ′+ĵκeff″]vΦ=λf=cIm[−κ′+jκeff″]Sd=cωRe[−κ′+jκeff″] 17

**Figure 17:**
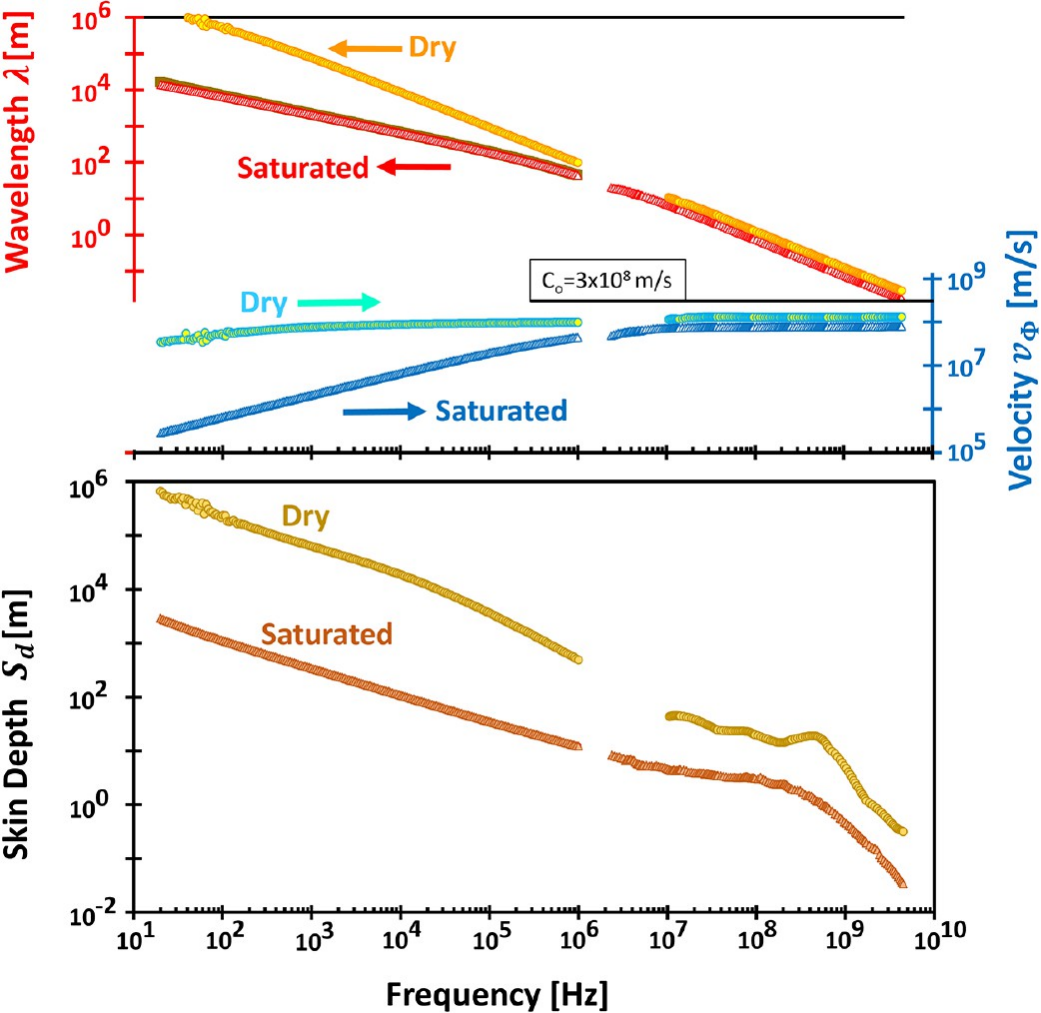
Wave characteristics spectra of a dry and saturated porous media: wavelength λ, phase velocity v_Φ_, and skin depth *S_d_*. Sample: carbonate rock.

Note that the skin depth *S*_*d*_ is the depth at which the wave amplitude decays by a factor of 1/*e*≈ 0.368.

### 5.1 Electrode-Based Measurements

#### Lab-Scale

Electrical properties at the nanometer to submicron scale are measured using AFM in electrical modes, where the cantilever tip acts as one electrode of a tiny capacitor scanning a grounded surface:^147,170^ *C*_*AFM*_ = ε_*s*_ *A*_*AFM*_/*d*_*AFM*_. The measured forces depend on material permittivity ε_*s*_, tip–surface contact area *A*_*AFM*_, and distance between the tip and surfaces *d*_*AFM*_ which reflects surface roughness.

For millimeter to meter-scale samples, two-, three-, or four-electrode methods are used (Figure 10), with geoscientists favoring two- and four-electrode setups. The two-electrode method uses simple electrode pairs, while the four-electrode method separates voltage and current electrodes for more precise measurements (Table 2).

**Table 2:**
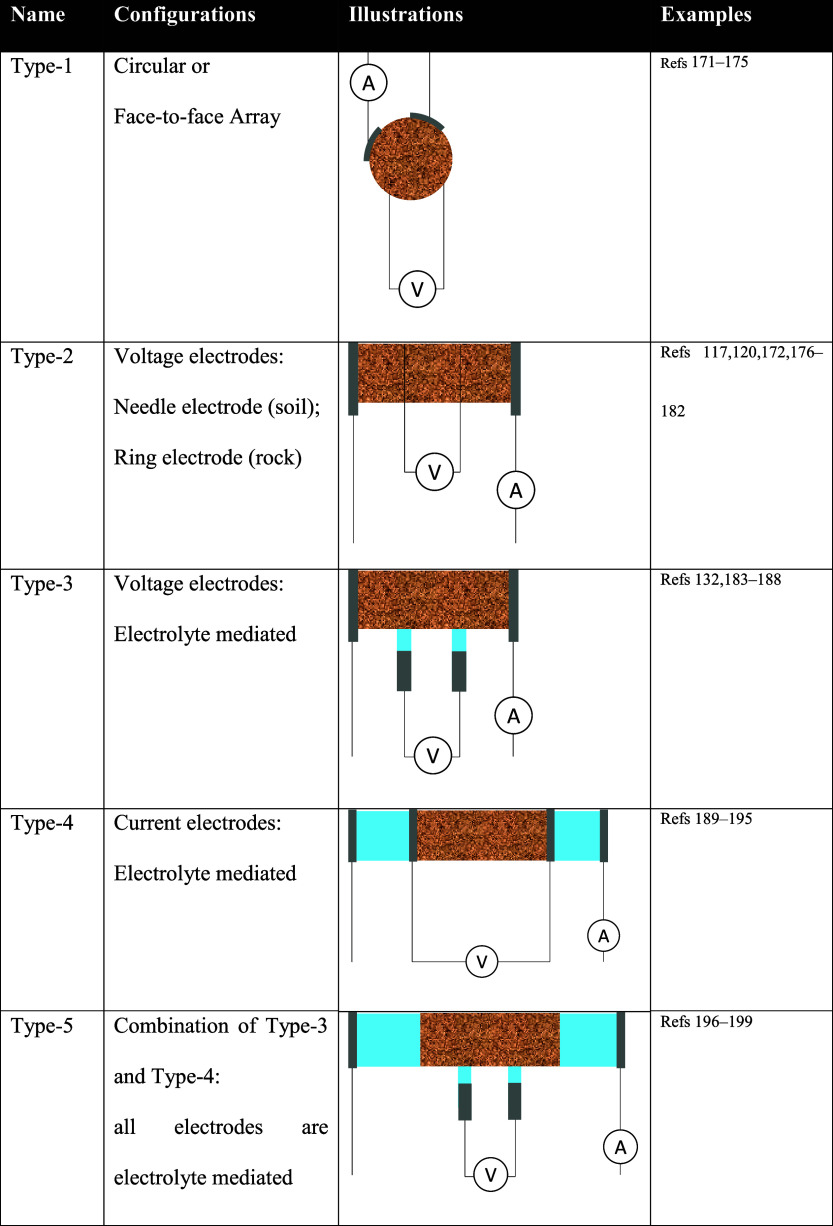
Electrode Configurations Commonly Used in Laboratory Measurement: The Four-Electrodes Method^171,172,173,174,175,176,177,178,179,180,181,182,183,184,185,186,187,188,189,190,191,192,193,194,195,196,197,198,199,200^

In electrochemistry, the three-electrode method is commonly used, where the positive voltmeter and ammeter share electrodes (Figure 10). The electrodes are termed as follows: working electrode (positive ammeter), working-sense electrode (positive voltmeter), reference electrode (negative voltmeter), and counter or auxiliary electrode (negative ammeter). Since the working electrode functions as the anode and is prone to corrosion, inert metals are preferred.

The effectiveness of the four-electrode method over the two-electrode setup for measuring wet porous media remains debated, both still exhibit significant electrode polarizations.^113,114^ Known as spectral induced polarization (SIP), the four-terminal method faces high-frequency challenges, such as inductive or parasitic capacitive coupling,^115^ which manifest as artificially high real permittivity κ^′^ due to phase errors (Figure 18). These are often attributed to cabling and instrument geometry, not just the sample.^115^

**Figure 18:**
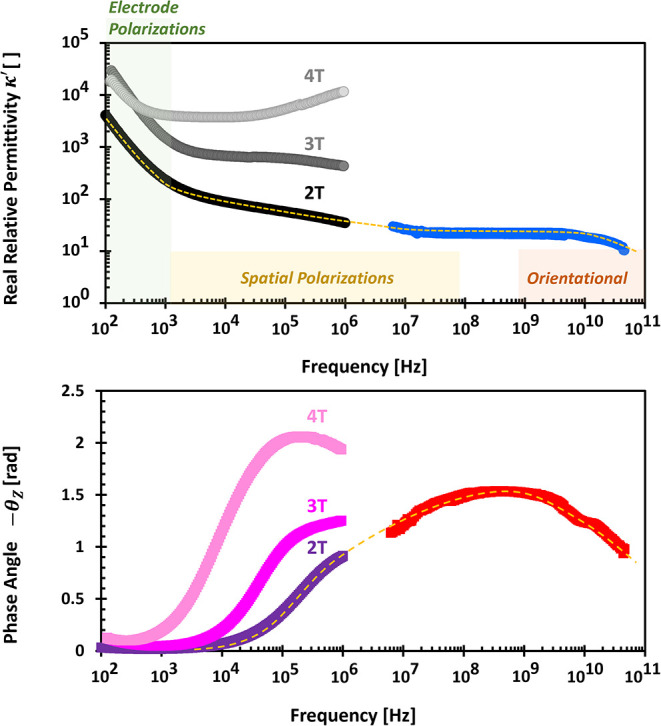
Permittivity and phase angle for various terminal configurations. Terminal is equivalent to electrode: 2T (two-terminal/electrode), 3T (three-terminal/electrode), and 4T (four-terminal/electrode) methods. High-frequency data (>1 MHz) measured by network analyzers. Sample: deionized water saturated carbonate rock (W). Dashed yellow lines are only to guide the eyes.

Terms *inductive coupling*, *electromagnetic coupling*, or *inductive electromagnetic coupling* are used interchangeably in geosciences. It is often addressed by adding inductors to correct the phase and recover true material properties.^200,201^ Electromagnetic coupling becomes more significant in low-conductivity samples during spectral induced polarization measurements, particularly at higher frequencies, where less current flow increases sensitivity to external electromagnetic interference.^37,120,202^ The simpler two-electrode method avoids these high-frequency couplings, though it is more prone to electrode polarization.^192^

To determine true complex conductivity or permittivity, an equivalent circuit model is often used, representing the material as a parallel resistor–capacitor system (*R*∥*C*)^203^ in series with an electrode polarization circuit. This polarization appears as a low-frequency tail in Nyquist plots (*Z*′ vs – *Z* ^″^, Figure 19). Some studies add nonpolarizing layers (e.g., Teflon) to reduce polarization, though this introduces additional capacitive elements and complicates interpretation.^107,204^

**Figure 19:**
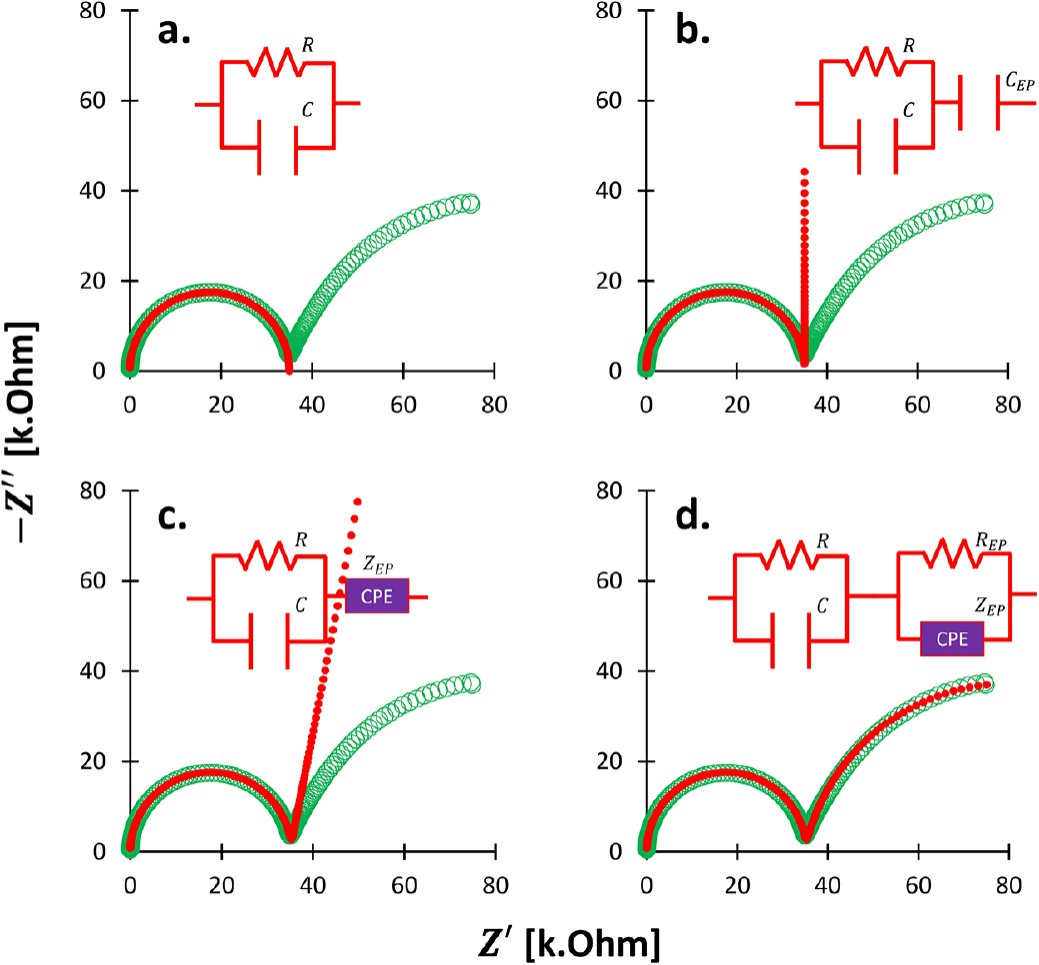
Nyquist plots. Green circles represent data of isopropanol measured by LCR meter (20 Hz to 2 MHz). The red dots are the models detailed by the corresponding circuits as insets.

The form of electrode polarization circuit can be (*R*∥*C*) or a parallel resistor–constant phase element (*R*∥*CPE*). The CPE impedance $Z_{CPE}^{*}=\frac{1}{{\left(j\omega \right)}^{q}\overset{∼}{A}}$ emerges between resistive (*q* = 0) and capacitive (*q* = 1) trait. For detailed modeling, refer to prior works.^205–207^

#### Field-Scale

At the field scale, the term resistivity ρ_eff_^*^ is more commonly reported than conductivity σ_eff_^*^ and is typically achievable from multielectrode measurements. Although modern systems automate the switching of electrode roles, the measurement principle is still based on the **four-electrode method.**

Table 3 lists typical electrode configurations used in field surveys, such as: **Two-Electrode, Pole–Pole, Pole-Dipole, Dipole–Dipole, Schlumberger**, and **Wenner,** each associated with a distinct electrode array and geometric factor β. A simplified form of β is approximately 2π*r*_*i*_ where *r*_*i*_ is the interelectrode spacing. This approximation assumes a hemispherical current flow (*A*≈ 1/2× 4π*r*_*i*_^2^) and penetrating depth *L* ≈ *r*_*i*_, leading to $V=\frac{I\rho_{\mathrm{eff}}^{*}}{\beta }\to V=\frac{I\rho_{\mathrm{eff}}^{*}}{2\pi r_{i}}$ .^208^ The general expression relating voltages, current, geometric factor, and electrode distances *d* is given by^209,210^

$$V_{MN}=V_{M}-V_{N}=\left(V_{M}^{+}-V_{M}^{-}\right)-\left(V_{N}^{+}-V_{N}^{-}\right)=\left(V_{AM}-V_{BM}\right)-\left(V_{\mathrm{AN}}-V_{BN}\right)=\frac{I{\rho^{*}}_{eff}}{2\pi }\left[\left(\frac{1}{d_{AM}}-\frac{1}{d_{BM}}\right)-\left(\frac{1}{d_{\mathrm{AN}}}-\frac{1}{d_{BN}}\right)\right]=\frac{{I\rho^{*}}_{eff}}{\beta }$$ 18

**Table 3:**
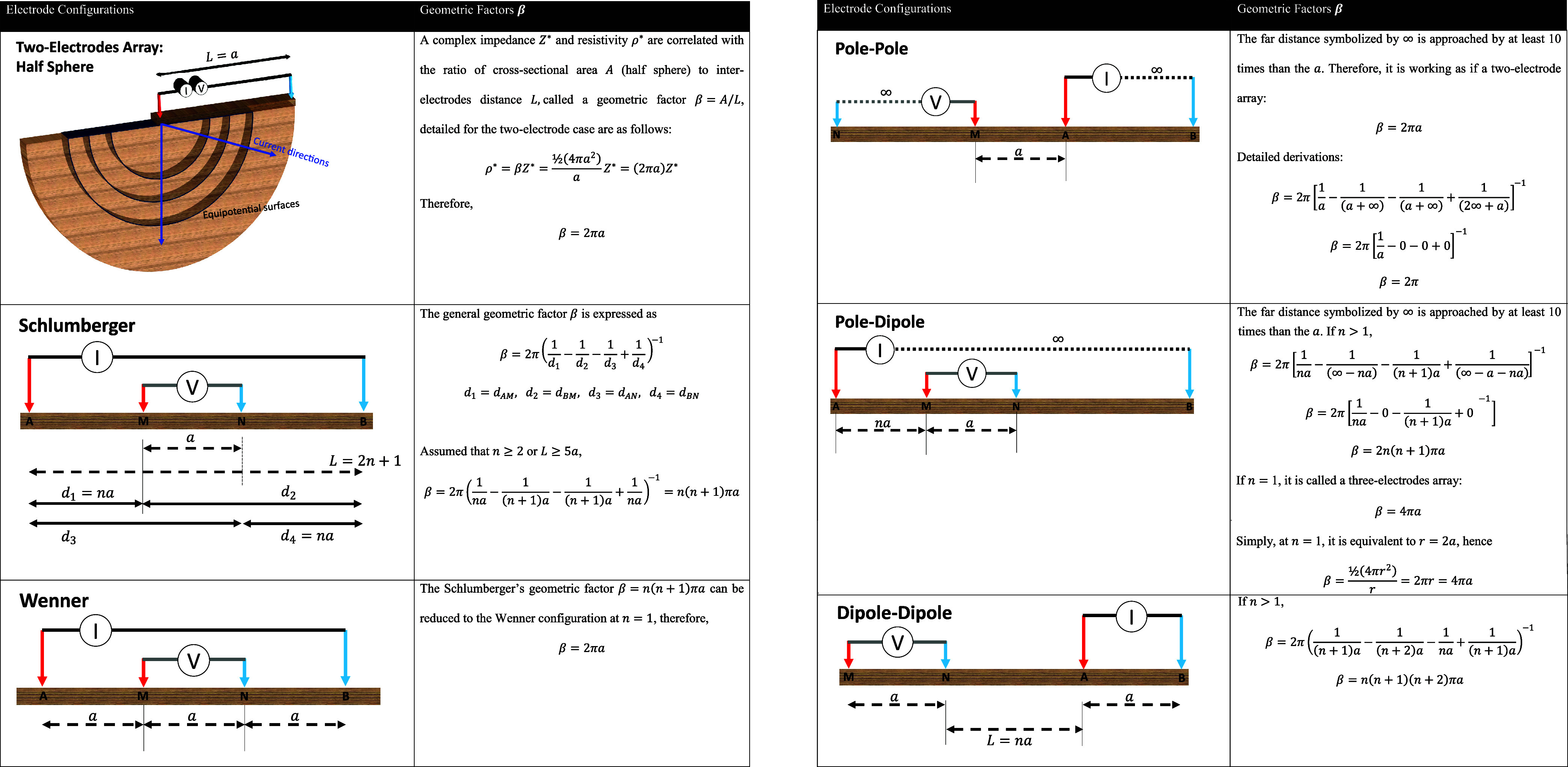
Electrode Configurations Commonly Used in Field Measurements^a^

a The term configuration is equivalent to array. The amperemeter + and – electrodes refer to the A and B electrodes. The voltmeter + and – electrodes refer to the M and N electrodes.

This formulation (eq 18) is widely applied to calculate both **DC resistivity** and **complex resistivity** ρ_eff_^*^, particularly in **spectral induced polarization** studies. Here, **M** and **N** denote the potential (voltage) electrodes, and **A** and **B** the current electrodes.

The dipole–dipole array provides superior lateral resolution, making it particularly effective for detecting vertically oriented subsurface features such as faults, fractures, dykes, and buried walls.^211^ In contrast, the Schlumberger array offers the best vertical resolution and greater depth penetration, rendering it suitable for imaging deep, horizontally stratified geological layers.^211^ The Wenner array, characterized by balanced sensitivity, exhibits good vertical resolution and is well-suited for delineating shallow, layered horizons near the surface.^211^ The choice of array should therefore be aligned with the geological target and the objectives of the investigation.

Field-scale electrical measurements extend beyond point or line profiles, such as those in well logging, to include 2D and 3D resistivity contrasts obtained through methods like resistivity tomography,^212^ cross-hole tomography,^213^ time-domain induced polarization (TDIP),^214^ and spectral induced polarization (SIP).^215^ These are active techniques requiring current injection and potential recording. In contrast, spontaneous potential (SP) is a passive method that records naturally occurring potentials.^210,216,217^

TDIP applies a periodic on–off current and measures voltage decay after current termination (Figure 20). The area under the decay curve *V*_*S*_(*t*) over time reflects the material’s chargeability, which characterizes its capacity to store electrical charge. Three common chargeability measures include: dimensionless total chargeability *M* [mV/V], partial chargeability *M*_12_ [ms], and integral chargeability *M̂* [ms]:^6,31^

$$\{\begin{array}{c} M=\frac{V_{i}}{V_{0}}=\frac{V_{0}-V_{\infty }}{V_{0}}=\frac{\rho_{0}-\rho_{\infty }}{\rho_{0}}=\frac{\sigma_{\infty }-\sigma_{0}}{\sigma_{\infty }}\\ M_{12}=\frac{1}{V_{0}}\int_{t_{1}}^{t_{2}}V_{S}\left(t\right)dt\\ \mathrm{M̂}=\frac{1}{V_{0}}\int_{t_{0}}^{t_{\infty }}V_{S}\left(t\right)dt \end{array}$$ 19

**Figure 20:**
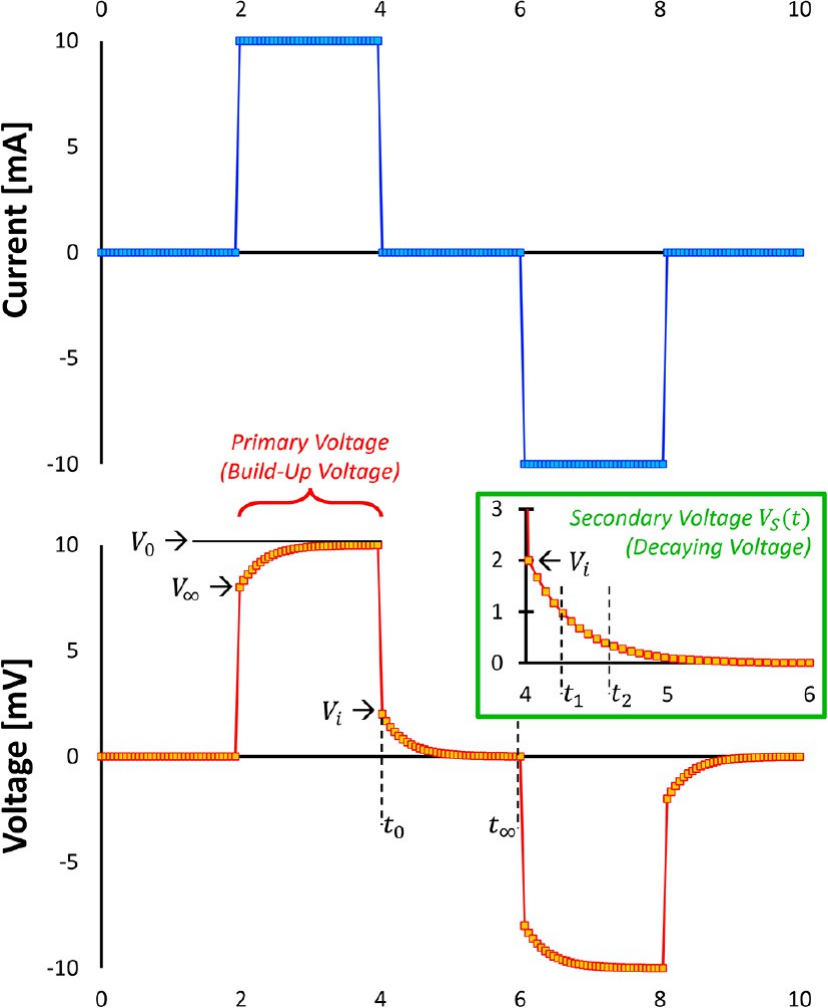
Applied current and voltage responses in time domain induced polarization (TDIP).

According to Ohm’s law, total chargeability *M* can be expressed in terms of resistivity, conductivity, or permittivity. For instance, *V*_∞_ = *IZ*_∞_ = *I*ρ_∞_β^–1^ = *I*σ_∞_^–1^β^–1^. Chargeability *M* can also be incorporated into the Cole–Cole model in conductivity form:

$$\sigma_{CC}^{*}=\overset{N}{\underset{i}{\sum }}\left[\sigma_{\infty_{i}}+\frac{{\sigma_{0}}_{i}-\sigma_{\infty_{i}}}{1+{\left(j\omega \tau_{i}\right)}^{\delta_{i}}}\right]=\overset{N}{\underset{i}{\sum }}\sigma_{\infty_{i}}\left[1-\frac{M_{i}}{1+{\left(j\omega \tau_{i}\right)}^{\delta_{i}}}\right]$$ 20

The resistivity version of this model is analogous yet not equal to the Pelton model, ρ_P_^*^ ≠ 1/σ_CC_^*^.^119,218^

Typical integral chargeability values (in ms) are 0 for groundwater, 3–12 for sandstones, 10–20 for limestones, 50–100 for shales, and 100–500 for siltstones (measured with 3 s charge times and 0.02–1.0 s decay windows^219^). Thus, TDIP is widely applied to assess clay content due to strong correlations between *M̂* and clay properties.

SIP operates across a broad frequency range (mHz to kHz) and produces frequency-selective tomographic images (Figure 2). It outperforms ground-penetrating radar (GPR) in conductive media such as clay-rich soils.^220^

### 5.2 Wave Propagation

#### Lab-Scale

Time Domain Reflectometry (TDR) is a wave propagation-based technique that enables the measurement of electrical conductivity and permittivity of materials. Its operating principle is grounded in transmission line theory, which describes the relationships among voltages at the steady state *V*_∞_, at the source *V*_*s*_, the resistances of the sample *R*, the inner line *R*_*i*_ and the cable *R*_*C*_:^221,222^

$$V_{\infty }=\frac{R+R_{C}}{R+\left(R_{i}+R_{C}\right)}V_{s}$$ 21

The TDR reflection coefficient ρ_*TDR*_^′^ is defined as the ratio between the difference in measured voltage (*V* – *V*_*o*_) and sampling voltage at the output interface *V*_*o*_:

$$\rho_{TDR}^{\prime }=\frac{V-V_{o}}{V_{o}}=\frac{V}{V_{0}}-1$$ 22

In the case of perfect transmission with no reflections, the output voltage is half the source voltage, i.e., *V*_*o*_ = *V*_*s*_/2. Substituting this into eq 22 gives $\rho_{TDR}^{\prime }=\frac{2V}{V_{s}}-1\to \frac{V}{V_{s}}=\frac{1+\rho_{TDR}^{\prime }}{2}$ . This leads to the expressions: $\frac{V_{\infty }}{V_{s}}=\frac{1+\rho_{TDR,\infty }^{\prime }}{2}$ and $\frac{V_{\infty ,SC}}{V_{s}}=\frac{1+\rho_{TDR,\infty ,SC}^{\prime }}{2}$ .

By substituting eq 21 into the resistance-conductivity relationship R = (βσ_*DC*_)^−1^, one can derive an expression for the DC electrical conductivity:^221,223^

$$\{\begin{array}{c} \sigma_{DC}=\frac{1}{\beta R_{i}}\left[\frac{\left(\frac{1}{V_{\infty }/V_{s}}-1\right)}{1-\frac{R_{C}}{R_{i}}\left(\frac{1}{V_{\infty }/V_{s}}-1\right)}\right]=\frac{1}{\mathrm{\beta ̂}}\left[\frac{\left(\frac{1-{\rho_{TDR,}^{\prime }}_{\infty }}{1+{\rho_{TDR,}^{\prime }}_{\infty }}\right)}{1-\frac{R_{C}}{R_{i}}\left(\frac{1-{\rho_{TDR,}^{\prime }}_{\infty }}{1+{\rho_{TDR,}^{\prime }}_{\infty }}\right)}\right]\\ \frac{R_{C}}{R_{i}}=\frac{1}{\left(\frac{1}{V_{\infty ,SC}/V_{s}}-1\right)}=\frac{1+{\rho_{TDR,}^{\prime }}_{\infty ,SC}}{1-{\rho_{TDR,}^{\prime }}_{\infty ,SC}} \end{array}$$ 23

where the cable-to-inner resistances ratio *R*_*C*_/*R*_*i*_ is determined at the short-circuited probe (R = 0) and steady-state short-circuited voltage *V*_∞, *SC*_. Once a known conductivity is provided, a calibration constant β*^* = β*R*_*i*_ can be determined.

Permittivity, particularly prior to orientational permittivity, is valuable for assessing water content in wet porous media, which its constant value ranging between 0.01 and 1.5 GHz (Figure 14b). Instruments based on electromagnetic wave propagation, such as the vector network analyzer^37,113^ and TDR,^222^ are capable of probing these frequencies. In practical applications, an apparent permittivity κ_*app*_ is considered, governing the wave velocity *v*_*m*_ through the medium. This can be calculated using the TDR-measured travel time Δ*t* as follows:^222,224,225^

$$v_{m}=\frac{2L_{p}}{\Delta t}\to \frac{c}{\sqrt{\kappa_{app}}}=\frac{2L_{p}}{\Delta t}\to \kappa_{app}={\left(\frac{c\Delta t}{2L_{p}}\right)}^{2}$$ 24

Here, *c* is the speed of light in vacuum, and *L*_*p*_ is the length of the probe; the electromagnetic wave must travel a round trip distance 2*L*_*p*_ (Figure 21).

**Figure 21:**
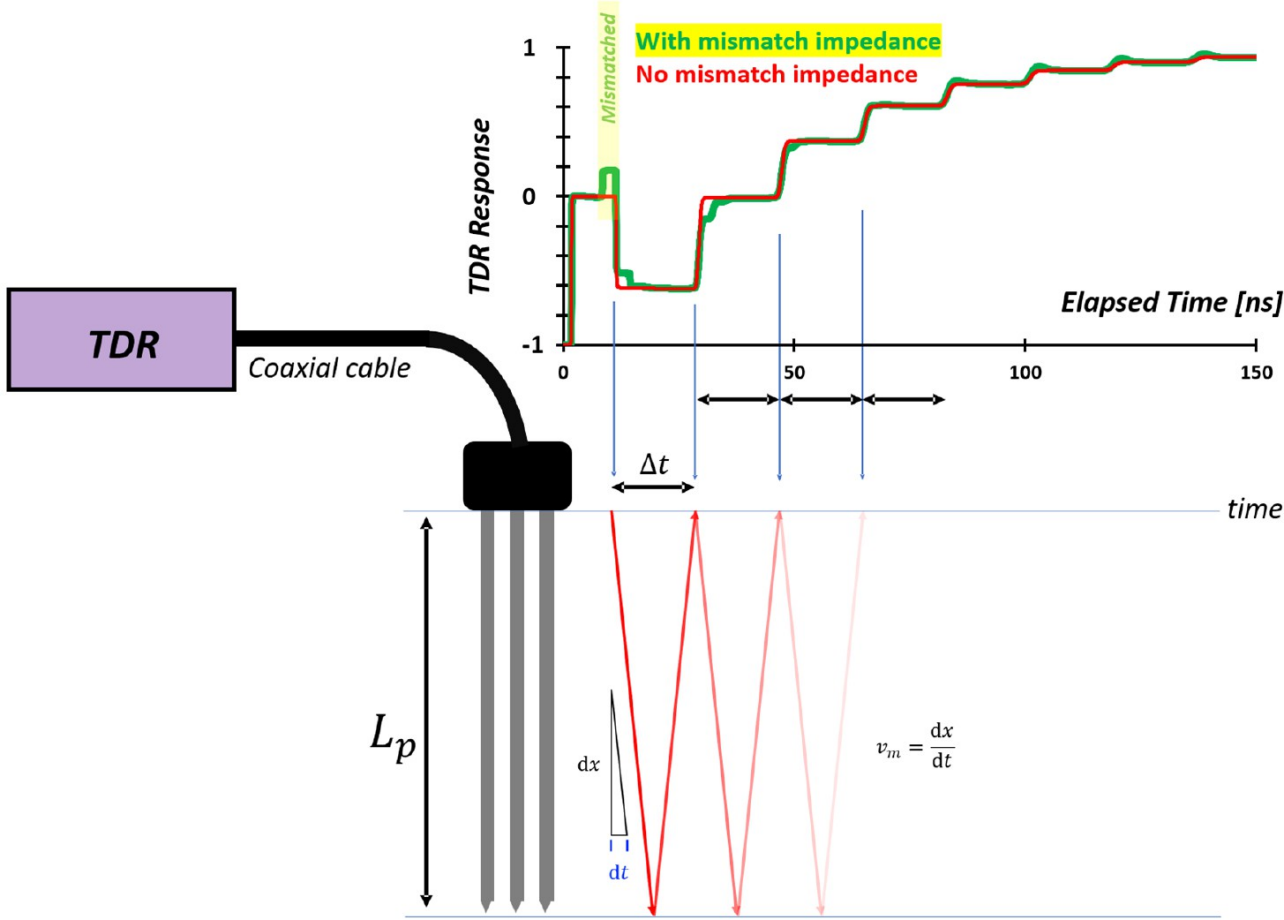
Time domain reflectometry (TDR). Typical set up, response in the time domain and its associated ray path (no mismatch path). The TDR response can be in voltage or normalized voltage, i.e., reflection coefficient ρ_TDR_^′^.

The effective frequency associated with κ_*app*_ is generally at the higher end of the TDR bandwidth (*f* > 100 MHz).^226^ Frequency-dependent permittivity, accounting for both real and imaginary components, can be obtained in the frequency domain using the phase difference ΔΦ = 2πfΔ*t* such that^227^

$$\kappa_{app}\left(f\right)={\left(\frac{c}{2L_{p}}\frac{\Delta \Phi }{2\pi f}\right)}^{2}$$ 25

At higher frequencies, the imaginary component κ^″^ becomes negligible, and the real part κ^′^ ≈ κ_*app*_(*f*) dominates. Correlations between water content and κ^′^ at such frequencies are generally more reliable than those based on time-domain κ_*app*_.

To enhance the spectral resolution of permittivity and conductivity retrieval, advanced signal processing techniques have been developed to extract complex permittivity values (both real and imaginary components) as functions of frequency. These include full-waveform analysis for determining Cole–Cole parameters,^228^ full-waveform analysis,^222^ multireflection analysis,^229^ dual-reflection analysis,^230^ and reflection-decoupled ratio.^169^ The short-open-load (SOL) TDR calibration technique^231^ mirrors that of vector network analyzers, which operate in the frequency domain using the bilinear form of the scattering parameter *S*_11_(ω).^232^ These methods has been applied in both low-loss^233^ and high-loss media.^234^

#### Field-Scale

TDR is a wave propagation-based technique commonly used for measuring electrical conductivity and permittivity. While suitable for field applications, it necessitates direct contact with the material under investigation, as the TDR probe functions as an electrode or dipole antenna that must penetrate the medium. In contrast, several field-scale electromagnetic techniques enable contactless measurement of electrical properties. These include ground penetrating radar (GPR),^235,236^ electromagnetic sounding,^237^ airborne electromagnetic,^238^ and the slingram method.^239^ A comprehensive summary of time- and frequency-domain EM methods used at field scale is available in a previous work.^218^

The operational frequency ranges of these methods are illustrated in Figure 2. GPR typically functions within the hundreds of MHz to a few GHz, whereas EM sounding, airborne EM, and slingram techniques operate at significantly lower frequencies, thereby achieving greater penetration depths at the cost of spatial resolution. GPR-derived phase velocity *v*_Φ_ and attenuation coefficient β* can be used to compute the complex relative permittivity κ* through eq 17. These values typically correspond to frequencies below the threshold of orientational polarizations.^240,241^ The attenuation coefficient is related to the electromagnetic skin depth as *S*_*d*_ = 1/β*.

Electromagnetic (EM) sounding,^242^ airborne electromagnetic,^243^ and slingram^239^ rely on transmitting EM fields using loop antennas. In accordance with Ampère’s Law, the transmitter coil generates a primary magnetic field that induces eddy currents in the subsurface based on Lenz’s Law. The magnitude of these eddy currents is a function of the electrical conductivity (e.g., influenced by metal content, salinity, or clay) and magnetic permeability μ of the ground. Magnetic susceptibility is defined as χ_*m*_ = μ/μ_0_– 1. These eddy currents, in turn, produce secondary magnetic fields that are detected by the receiver coil, consistent with Faraday’s Law. A notable passive EM method is magnetotellurics, which relies solely on natural EM fields and requires only receiver deployment.^244^

The selection of a suitable geophysical technique depends on aligning spatial resolution, target depth, wavelength λ and skin depth *S*_*d*_, as discussed in Figure 2 and Figure 17. The propagation and attenuation of EM waves are governed by the medium’s complex conductivity and permittivity.

The electric field component ***E*** obeys the wave equation derived from Maxwell’s equations:

$$\nabla^{2}E=\mu \sigma \frac{\partial E}{\partial t}+\mu \varepsilon \frac{\partial^{2}E}{\partial t^{2}}$$ 26

In one-dimensional cases, the ∇^2^ ***E*** reduces to ∂^2^ ***E***/∂*x* ^2^. In highly conductive media (σ → ∞), the wave equation simplifies to a diffusion-like form: $\frac{\partial^{2}E}{\partial x^{2}}=\mu \sigma \frac{\partial E}{\partial t}$ . Under such conditions, the electromagnetic wave becomes heavily attenuated, reducing its amplitude and skin depth. This limits the ability to retrieve permittivity from behind highly conductive layers (e.g., metallic casings), potentially rendering such measurements infeasible.

## 6 Engineering Implications

This section discusses the use of conductivity and permittivity to infer hydraulic properties and their constituents. We emphasize the hydraulic properties and constituents that have a direct relationship or causality with electrical properties, as derived from earlier hydrodynamic and transport studies.^27^ These properties include porosity, water content, specific surface area, cation exchange capacity, tortuosity, salt concentrations, clay types, clay contents, and metal inclusions. We also address the use of conductivity contrast in subsurface imaging.

Particle and fluid transport in the subsurface is dominated by advection, diffusion, dispersion, and capillarity. Although organic polymeric materials may exhibit mobility within the subsurface, their transport is more accurately described by hydrodynamic transport models, particularly under unsaturated flow condition^27,245^ , than by mechanisms driven by electric fields (electrophoretic and dielectrophoretic transport mechanisms).

### 6.1 Porosity, Water Content, and Specific Surface Area

Dry rock-forming minerals exhibit low electrical conductivity σ_*m*_ (10^–8^ to 10^–4^ S/m), while saline pore fluids σ_*f*_ range from 10^–3^ to >1 S/m. In clay-rich or high specific surface area *S*_*S*_, surface conductivity quantified as Γ*S*_*S*_, can significantly influence total conductivity, especially under low-salinity conditions (e.g., freshwater). In such cases, model in eq 11 is applicable. However, when fluid conductivity exceeds 1 S/m, surface effects become negligible, and total conductivity is primarily controlled by porosity (Figure 22 and Figure 23).

**Figure 22:**
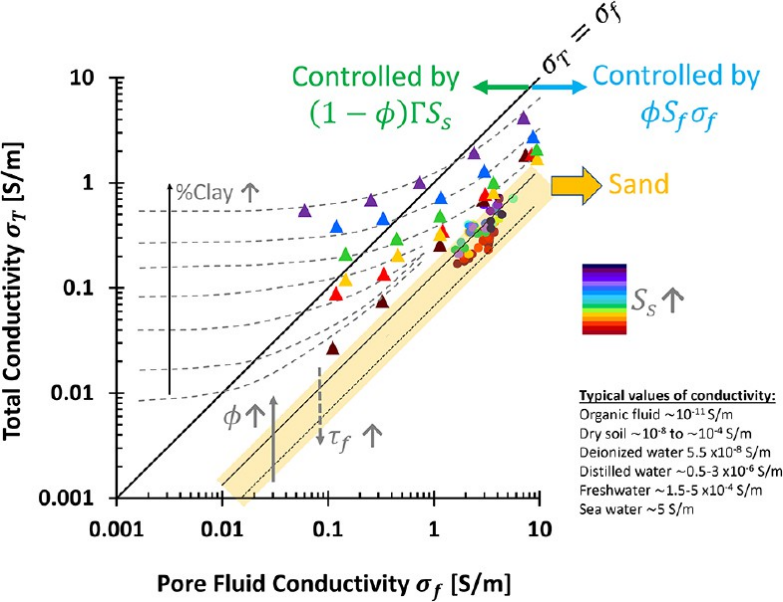
Total vs pore fluid conductivity. Data sources: (circle solid) ref 261 and (triangle up solid) ref 361.

**Figure 23:**
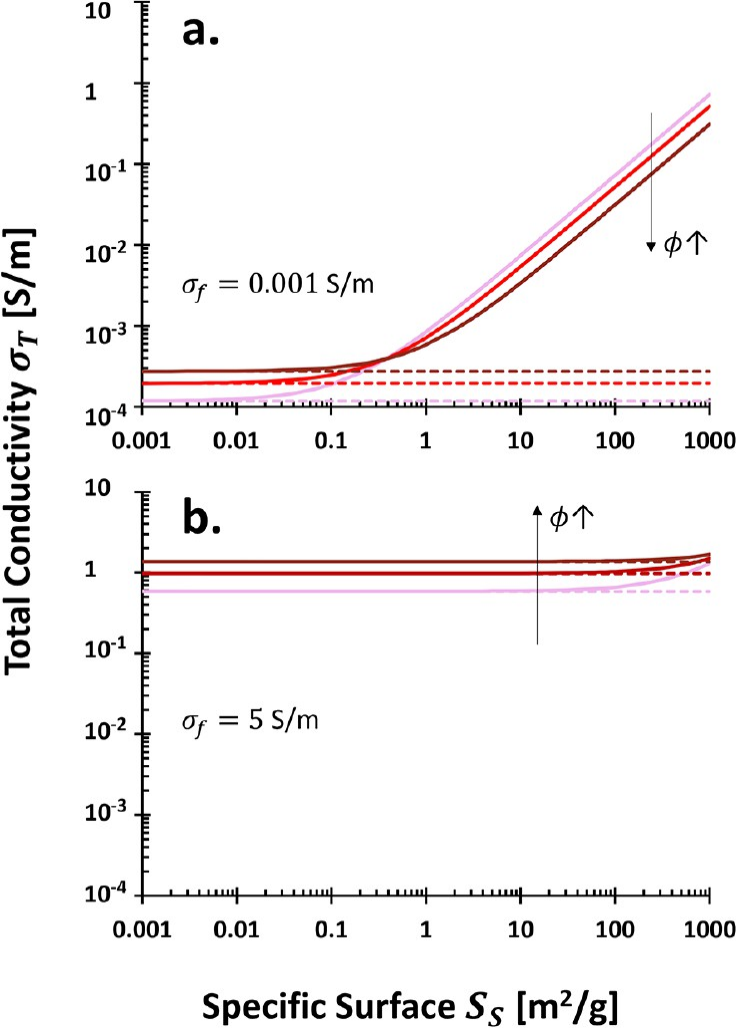
Specific surface area effects on the total conductivity. Model refers to eq 9 with σ̂_S_λ_D_ = 10^–9^ Siemens. (a) Pore fluid is at 10^–3^ S/m. (b) Pore fluid is at 5 S/m. The porosity ranges are 0.3, 0.5, and 0.7, which correspond to pink, red, and brown solid lines. The pore fluid conductivities are indicated by dashed lines.

In fully saturated porous media (*S*_*w*_ = 1), the volumetric water content θ_*w*_ = ϕ*S*_*w*_ equals the porosity ϕ. For clean, saturated conditions, porosity can be approximated from either total conductivity (σ_*T*_ = σ_*w*_ϕ) or bulk permittivity (κ^′^ = κ_*w*_^′^ϕ) as a first-order estimate. However, subsurface saturation is typically unknown, introducing uncertainty. The cementation factor *m* in Archie’s equation: $F=\frac{a}{\phi^{m}}$ , helps fit data even without precise saturation values (Figure 24). For unsaturated materials, total conductivity decreases, leading to a higher formation factor *F* ∝ σ_*T*_^–1^.

**Figure 24:**
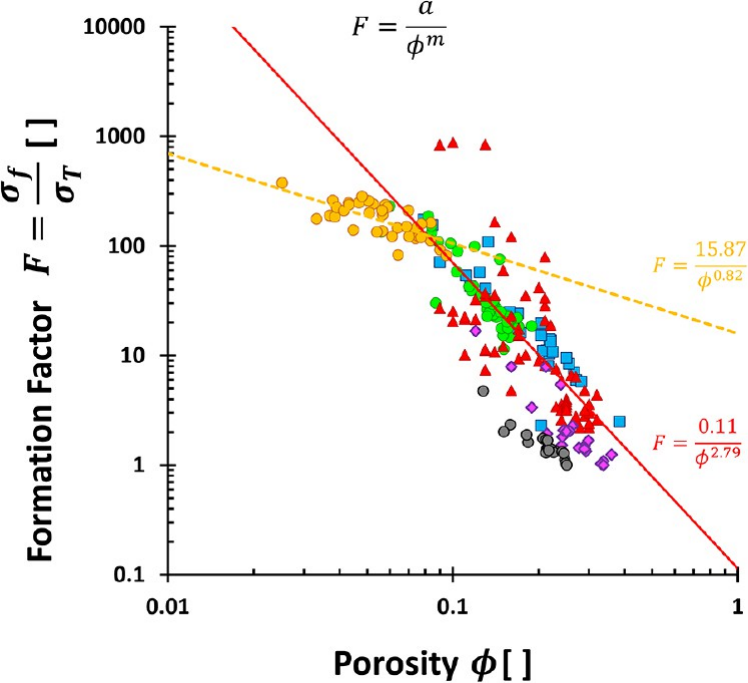
Archie’s equation in the formation factor form: $F=\frac{a}{\phi^{m}}$ . Note that the formation factor is proportional to the inverse bulk conductivity: *F* = σ_T_^–1^σ_f_ or proportional to bulk resistivity: *F* = ρ_B_ρ_f_^–1^. Some formation factor conversions use the assumed pore fluid conductivity σ_f_ is the smallest measured conductivity within the data set. Data sources: refs 27,220 .

The cementation factor, also known as the porosity exponent *m*, varies according to the type and structure of the porous medium. Typical values reported in the literature include: 1.5–3.0 for carbonates,^246^ 1.6–1.9 for clean sands,^247^ ∼ 1 for fractured rocks, ∼ 2 for mudstones, 1.8–5.4 for moldic lime,^248^ and 1.55–2.11 for sand-kaolinite mixtures.^249^ More compilation of Archie’s exponent *m* refer to a previous work.^250^

For the purpose of model fitting, eq 11 is reformulated by introducing empirical constants {α, *n*}, and by expressing the porosity as the reciprocal of formation factor, ϕ = 1/*F*, with assumed *a* = 1, *m* = 1. The modified model is given by

$$\sigma_{T}=\frac{\sigma_{f}^{\alpha }S_{f}^{n}}{F}+\Gamma S_{S}\left(1-\frac{1}{F}\right)$$ 27

This formulation facilitates comparison with a range of empirical and semiempirical models commonly employed in petrophysical analyses. Table 4 provides a summary of widely used conductivity models in the context of well logging, where the measured quantity is typically expressed as resistivity (see the compilations in refs 251,252 ).

**Table 4:** List of Typical Resistivity Equations Used in Well Logging and Rock Physics^a^

Name of equations [references]	Water saturation or Resistivity models (typical nomenclature: function of resistivity)	Rearranged equation in conductivity expression Note: conductivity **σ*T*** =1 ***/ρT*** inverse of resistivity
Archie^101^	$S_{w}=\sqrt[{n}]{\frac{a}{\phi^{m}}\frac{\rho_{w}}{\rho_{T}}}\to \frac{1}{\rho_{T}}=\frac{1}{F\rho_{w}}S_{w}^{n}$	$\sigma_{T}=\frac{1}{a}\phi^{m}S_{w}^{n}\sigma_{w}\to \sigma_{T}=\frac{1}{F}S_{w}^{n}\sigma_{w}$
		Comments: σ_*T*_ = *f*(σ_*w*_)
Simandoux^251,253^	$S_{w}=\sqrt{\left(\frac{1}{\rho_{T}}-\frac{xV_{sh}}{\rho_{sh}}\right)F\rho_{w}}\to \frac{1}{\rho_{T}}=\frac{1}{F\rho_{w}}S_{w}^{2}+\frac{xV_{sh}}{\rho_{sh}}$	$\sigma_{T}=\frac{1}{F}S_{w}^{2}\sigma_{w}+xV_{sh}\sigma_{sh}$
		Comments: σ_*T*_ = *f*(σ_*w*_) + *f*(σ_*S*_)
Modified Simandoux by Bardon and Pied^254^	$\frac{1}{\rho_{T}}=\frac{S_{w}^{2}}{F\rho_{w}}+\frac{V_{sh}S_{w}}{\rho_{sh}}$	$\sigma_{T}=\frac{1}{F}S_{w}^{2}\sigma_{w}+V_{sh}S_{w}\sigma_{sh}$
		Comments: σ_*T*_ = *f*(σ_*w*_) + *f*(σ_*S*_)
Modified Simandoux by Poupon and Leveaux (Indonesian Equation)^255^	$\frac{1}{\rho_{T}}={\left(\sqrt{\frac{1}{F\rho_{w}}}+\sqrt{\frac{V_{sh}^{x}}{\rho_{sh}}}\right)}^{2}S_{w}^{n}$	$\sigma_{T}={\left(\sqrt{\frac{1}{F}\sigma_{w}}+\sqrt{V_{sh}^{x}\sigma_{sh}}\right)}^{2}S_{w}^{n}$
		Comments: σ_*T*_ = *f*(σ_*w*_) + *f*(σ_*S*_)
Modified Simandoux by Schlumberger^256^	$\frac{1}{\rho_{T}}=\frac{S_{w}^{2}}{F\left(1-V_{sh}\right)\rho_{w}}+\frac{V_{sh}S_{w}}{\rho_{sh}}$	$\sigma_{T}=\frac{1}{F\left(1-V_{sh}\right)}S_{w}^{2}\sigma_{w}+V_{sh}S_{w}\sigma_{sh}$
		Comments: σ_*T*_ = *f*(σ_*w*_) + *f*(σ_*S*_)
Poupon et al.^257^	$\frac{1}{\rho_{T}}=\frac{\left(1-V_{sh}\right)S_{w}^{n}}{F\rho_{w}}+\frac{V_{sh}}{\rho_{sh}}$	$\sigma_{T}=\frac{\left(1-V_{sh}\right)}{F}S_{w}^{n}\sigma_{w}+V_{sh}\sigma_{sh}$
		Comments: σ_*T*_ = *f*(σ_*w*_) + *f*(σ_*S*_)
Waxman–Smits^258^	$\frac{1}{\rho_{T}}=\frac{S_{w}^{2}}{F\rho_{w}}+\frac{BQ_{V}S_{w}}{F}$	$\sigma_{T}=\frac{S_{w}^{2}}{F}\sigma_{w}+\frac{BQ_{V}S_{w}}{F}$
		Comments: σ_*T*_ = *f*(σ_*w*_) + *f*(σ_*S*_)
Dual-water model^259^	$\frac{1}{\rho_{T}}=\frac{S_{w}^{n}}{F}\left[\frac{1}{\rho_{w}}+\frac{V_{Q}Q_{V}}{S_{w}}\left(\frac{1}{\rho_{w,c}}-\frac{1}{\rho_{w}}\right)\right]$	$\sigma_{T}=\frac{S_{w}^{n}}{F}\left[\sigma_{w}+\frac{V_{Q}Q_{V}}{S_{w}}\left(\sigma_{w,c}-\sigma_{w}\right)\right]$
		Comments: σ_*T*_ = *f*(σ_*w*_) + *f*(σ_*S*_)
Klein-Santamarina^84^	Follows the conductivity equation: $\frac{1}{\rho_{T}}=\sigma_{T}$	σ_*T*_ = σ_*f*_ϕ + (σ_*m*_ + σ_*S*_ *S*_*s*_ρ_*m*_)(1 – ϕ)
		Unit of σ_*S*_ is [S]
		Comments: σ_*T*_ = *f*(σ_*w*_) + *f*(σ_*m*_) + *f*(σ_*S*_)
Choo-Burns^260^	Follows the conductivity equation: $\frac{1}{\rho_{T}}=\sigma_{T}$	$\sigma_{T}=\sigma_{f}\phi \frac{1}{\tau^{2}}+\left(\sigma_{m}\frac{L^{2}}{L_{m}^{2}}+\sigma_{S}S_{S}\rho_{m}\frac{1}{\tau^{2}}\right)\left(1-\phi \right)$
		Unit of σ_*S*_ is [S]
		Comments: σ_*T*_ = *f*(σ_*w*_) + *f*(σ_*m*_) + *f*(σ_*S*_)
Hakiki et al.^27^	Follows the conductivity equation: $\frac{1}{\rho_{T}}=\sigma_{T}$	Full equation: $\sigma_{T}=\sigma_{f}S_{f}\phi \frac{1}{\tau_{f}^{2}}+\sigma_{m}\left(1-\phi \right)\frac{1}{\tau_{m}^{2}}+{\mathrm{\sigma ̂}}_{S}\lambda_{D}S_{S}\rho_{m}\left(1-\phi \right)\frac{1}{\tau_{S}^{2}}$
		Units: σ*^*_*S*_ [S/m], λ_*D*_ [m], *S*_*S*_[m^2^/g], and ρ_*m*_[g/m^3^].
		Simplified: σ_*T*_ = σ_*f*_ *S*_*f*_ϕ + (σ_*m*_ + Γ*S*_*S*_)(1 – ϕ)
		Unit of the surface conduction factor Γ is [S·g·m^–3^], thus, the product Γ*S*_*S*_ is in [S/m], called surface conductivity.
		Comments: σ_*T*_ = *f*(σ_*w*_) + *f*(σ_*m*_) + *f*(σ_*S*_)

a The total electrical resistivity ρ_T_ unit is in Ohm·m and the conductivity σ_T_ unit is S/m. Frequently used subscripts: *T*: total or bulk, *w*: water, *f*: fluids, *sh*: shales or clay, *m*: solid minerals. Inverse of formation factor $\frac{1}{F}=\frac{1}{a}\phi^{m}\to \frac{1}{F}\propto \phi$ . The “comments″ part is used to summarize the contribution of the individual components: pore fluid-controlled *f*(σ_*w*_), mineral influences *f*(σ_*m*_), and clay-induced surface conduction *f*(σ_*S*_). Hakiki et al. model^27^ signifies the arbitrary degree of saturation *S*_*f*_, while Klein–Santamarina^84^ and Choo–Burns models^260^ are used for saturated media (*S*_*f*_ = 1).

Figure 25 shows complex conductivity σ* tomography of the 4 m subsurface. The conductivity magnitude |σ*| and in-phase component σ^′^ provide limited insight compared to the phase angle θ_*Z*_ and quadrature conductivity σ^″^, which better correlate with water content θ_*w*_. Validation was performed using 1 m subsurface samples from smectite-rich clay. A frequency of 1 Hz was selected for SIP tomography, where σ^″^ exhibits a local maximum and is marking the onset of low-frequency polarization observed in the real permittivity κ′ spectrum, likely due to Stern layer polarization in clays,^220,261^ though electrode polarization may also contribute.

**Figure 25:**
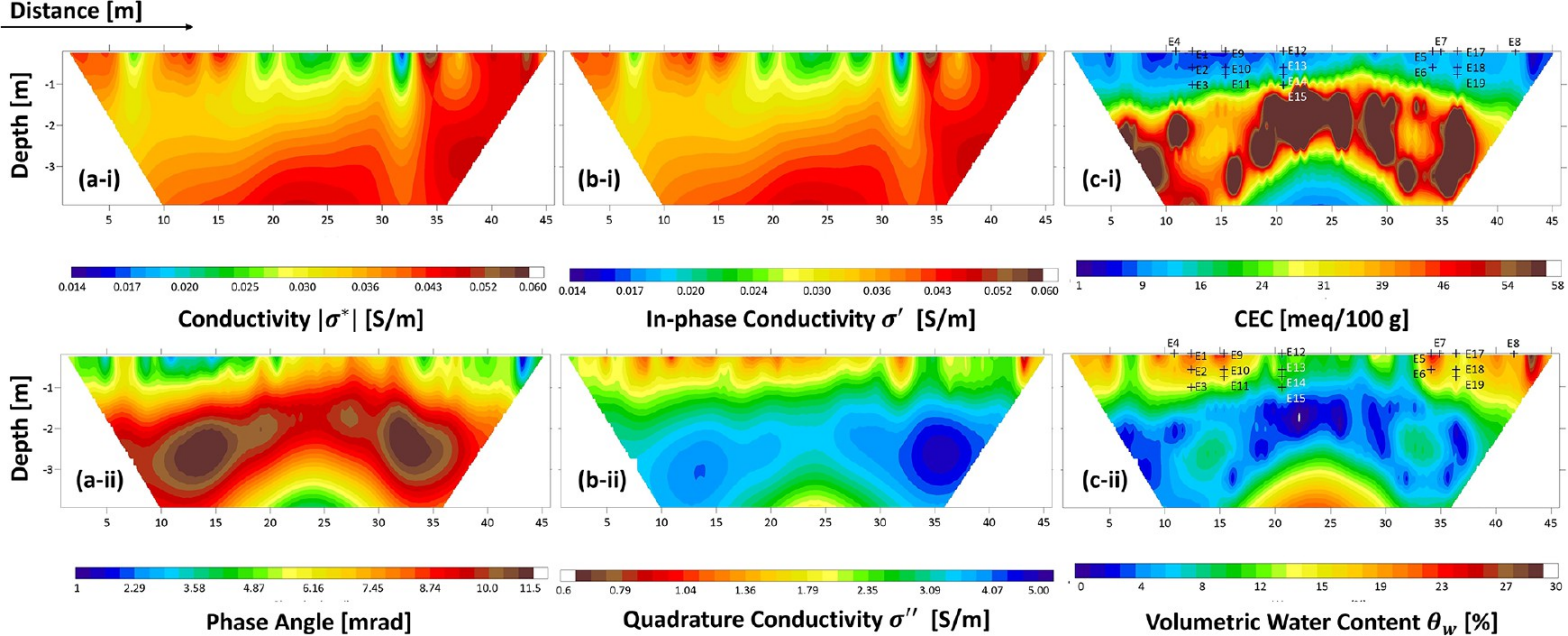
Spectral induced polarization tomography and inferred physical properties. The selected frequency is at 1 Hz. Reproduced with permission from ref 220. Copyright [2021] [Elsevier]. Personal permission to reuse has also been authorized by Prof. Andre Revil.

Bulk conductivity can be numerically estimated using discrete element-finite difference,^262^ fractal theory or percolation model,^263^ or effective medium theories,^264^ typically applied to CT or optical microscopy images of saturated porous media.^265^

Meanwhile, bulk permittivity κ^′^, apparent permittivity κ_*app*_, or complex permittivity κ*­(*f*) depends on the volumetric fractions of constituents and is commonly modeled using a generalized mixing model:

$$\{\begin{array}{c} {\left(\kappa \right)}^{1/c_{\kappa }}=\phi S_{f}{\left(\kappa_{f}^{\prime }\right)}^{1/c_{\kappa }}+\phi S_{a}{\left(\kappa_{a}^{\prime }\right)}^{1/c_{\kappa }}+f_{aw}{\left(\kappa_{aw}^{\prime }\right)}^{1/c_{\kappa }}+\left(1-\phi \right){\left(\kappa_{m}^{\prime }\right)}^{1/c_{\kappa }}\\ S_{f}+S_{a}+S_{af}=1 \end{array}$$ 28

where subscripts denote fluid (*f*), air (*a*), adsorbed liquid or water (*aw*), and mineral (*m*). The adsorbed fluid fraction,*f*_*aw*_ is derived from the volumetric ratio: *f*_*aw*_ = *V*_*aw*_/*V*_*T*_ = *t*_*w*_ *S*_*S*_ *m*_*m*_/*V*_*T*_ = *t*_*w*_ *S*_*S*_ρ_*m*_ *V*_*m*_/*V*_*T*_ = *t*_*w*_ *S*_*S*_ρ_*m*_(1 – ϕ), where *t*_*w*_ is the adsorbed water film thickness (1–20 nm^266^), κ_*m*_^′^∼3–6,^26,37^ and κ_*aw*_^′^∼2–20.^147,267^ The model: $\kappa_{aw}^{\prime }=\frac{t_{w}}{1nm}+1$ .^147^

We can substitute κ^′^ in eq 28 by κ_*app*_ or κ*­(*f*). The fitting constant *c*_κ_ adjusts model flexibility, e.g., 1 for parallel capacitors), 2 for CRIM: complex refractive index model, 3 for cubic, and −1 for series capacitors model. At water contents below 0.2, mineral surface charge suppresses permittivity. Above this threshold, free pore water dominates, and permittivity exceeds CRIM predictions (Figure 14b).

### 6.2 Cation Exchange Capacity

Surface conduction in porous media arises from excess electrical charge near mineral surfaces, primarily due to cation adsorption within the electrical double layer (Figure 6). This charge storage is quantified by the cation exchange capacity (CEC), which indicates the number of exchangeable cations a mineral surface can hold.^252^ CEC is inherently linked to specific surface area *S*_*S*_ and surface conductivity Γ*S*_*S*_, particularly in fine-grained materials like clays with high surface charge density.

A previous study showed that CEC correlates with each quadrature σ^″^ and surface conductivity: CEC ∝(σ^″^)*^n_q_^* and CEC ∝(Γ*S*_*S*_)^*n*_Γ_^ where *n*_*q*_, *n*_Γ_> 1.^220^ The dimensionless chargeability *M* also correlates with quadrature σ^″^ and surface conductivity Γ*S*_*S*_.^220,268^ Therefore, CEC links to chargeability: $M=\frac{\rho_{m}\mu^{+}\sigma_{\infty }\mathrm{CEC}}{F\phi }$ .^220,268^ These relationships enable estimation of CEC from conductivity data, highlighting why phase angle (Figure 25a-ii) and quadrature conductivity σ^″^ (Figure 25b-ii) are key indicators of CEC.

Empirical data further show a power-law relationship between CEC and specific surface area: CEC ∝*S*_*S*_^*n*_*S*_^ where *n*_*S*_ > 0.^268,269^ Under low ionic strength conditions, this becomes linear: CEC ∝*S*_*S*_.^70^ Geomaterials with either high CEC or specific surface area exhibit high permittivity κ^′^ that indicate polarizations in low-frequencies, attributed to overlapping diffuse double layer, interfacial, and electrode polarizations.

### 6.3 Tortuosity

Tortuosity τ_*f*_ is geometrically defined as the ratio of the actual fluid path length to the straight-line distance between inlet and outlet, τ_*f*_ = *L*_*f*_/*L*_*s*_ and the values must be greater than one.^270^ It can be defined based on geometric, hydraulic, electrical, and diffusional properties.^270^ According to eq 14, the formation factor $F=\frac{a}{\phi^{m}}=\frac{\tau_{f}^{2}}{\phi^{m}}$ indicating that tortuosity factor *a* = τ_*f*_^2^. Data in Figure 24 reveal τ_*f*_ < 1, which is physically invalid. This suggests that using Archie’s equation to determine tortuosity may be unreliable when saturation is uncertain. The red triangles in Figure 24 likely represent unsaturated samples. The values of *a* can span even 4 orders of magnitude:^24,271^ 0.004–17.7 in sandstones and 0.33–78.0 in carbonates.

The formation factor is formally defined as $F=\frac{\sigma_{f}}{\sigma_{T,1}}$ (eq 14), but due to unknown saturation, it is computed as $F=\frac{\sigma_{f}}{\sigma_{T}}$ . Values of *a* and τ_*f*_ below one likely result from both low pore fluid conductivity σ_*f*_ or and significant surface conduction Γ*S*_*S*_. Hence, predicting tortuosity in clay-rich geomaterials is unreliable.

Various methods exist to quantify tortuosity, including electrical tortuosity derived from resistivity,^101,272–275^ capillary pressure or *J*-function,^276^ permeability, porosity, specific surface area, and pore size distribution relationships,^277–281^ and digital image analyses.^282–284^ Various forms of physical tortuosity are often used interchangeably, despite distinct differences in their definitions and evaluation methodologies.^285^ Tortuosity validation remains ambiguous due to complex pore networks with merging and branching flow paths.^27^

Previous studies link the cementation factor *m* to tortuosity,^246,265^ with *m* < 1.5 indicating less tortuous paths (e.g., an open fracture) and *m* > 2 suggesting more tortuous ones. However, we interpret *m* as a fitting parameter that compensates for surface conduction and unknown saturation levels in subsurface samples.

### 6.4 Concentrations

#### Ionic Concentration

Pore fluid conductivity generally increases with salt concentration, particularly within the low-viscosity range. However, certain brines exhibit decreased conductivity at higher concentrations, indicating that conductivity depends on both ion concentration and mobility.^84^ At high salinity, ion mobility decreases despite increased ionic strength. As shown in Figure 22, higher pore fluid conductivity enhances bulk conductivity in wet, low-clay media. For clay-rich materials, pore fluid conductivity must exceed 1 S/m to dominate the total conductivity.

#### Clay Type and Content

Clay-rich geomaterials dominate bulk conductivity in porous media when the clay exhibits a specific surface area *S*_*S*_ > 0.4 g/m^2^ (Figure 23a) and pore fluid conductivity is low, σ_*f*_ < 1 S/m (Figure 22). Notably, in the specific surface-controlled regime, higher porosity corresponds to lower conductivity (Figure 23a).

In samples composed of a single clay type (constant *S*_*S*_), bulk conductivity still varies with clay concentration. Since *S*_*S*_ is an intrinsic (volume-independent) property, this variation is explained by the surface conduction factor Γ = *f*(σ_*S*_, λ_*D*_), an extrinsic (volume-dependent) property. Thus, at low ionic strength, bulk conductivity reflects both clay type and concentration, with Γ serving as a proxy for clay content.

Real permittivity κ^′^ at the suborientational polarization frequencies (10^4^–10^9^ Hz) is sensitive to water content (Figure 26a), but shows limited sensitivity to clay type or concentration (Figure 26b-c). In contrast, electrode polarization regimes reveal clear distinctions, as increased clay content or mineral surface area enhances the release of previously adsorbed ions. Consequently, samples with higher specific surface area clays (Figure 26b) or greater clay content (Figure 26c) exhibit stronger electrode polarization, reflected in elevated κ^′^ values.

**Figure 26:**
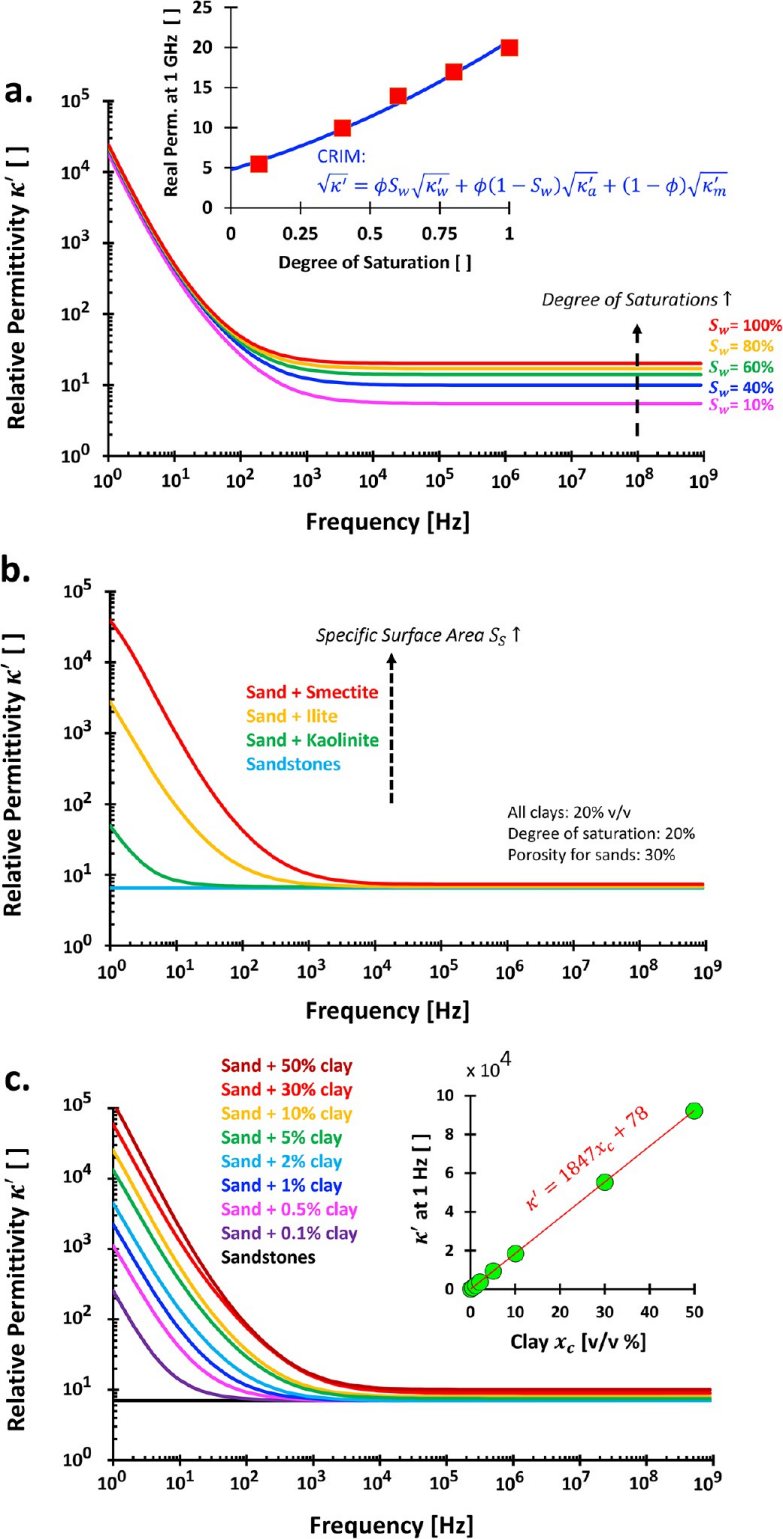
Effects of water and clay content on real permittivity spectra. (a) Effects of water content in term of degree of saturation in sands. Inset inside (a): Permittivity mixing model with CRIM (complex refractive index model); (b) Effects of clay types (specific surface area S_S_); (c) Effects of clay concentrations. Inset within (b): Real permittivity at 1 Hz versus clay concentrations. Plots are generated with the Cole–Cole equation, which approximates the measured data in ref 362.

#### Metal Content

Mineral conductivity σ_*m*_ is typically negligible but becomes significant in the presence of metallic precipitates (e.g., iron, iron oxides, hematite), such as in mine tailings.^286,287^ Granular zerovalent iron is widely used for removal of contaminants^288,289^ and viruses^290^ from groundwater. Hematite-coated kaolinite increases bulk electrical conductivity under low-salinity conditions (Figure 27a), but the coating has no effects in samples saturated by a very conductive fluid (Figure 27b).

**Figure 27:**
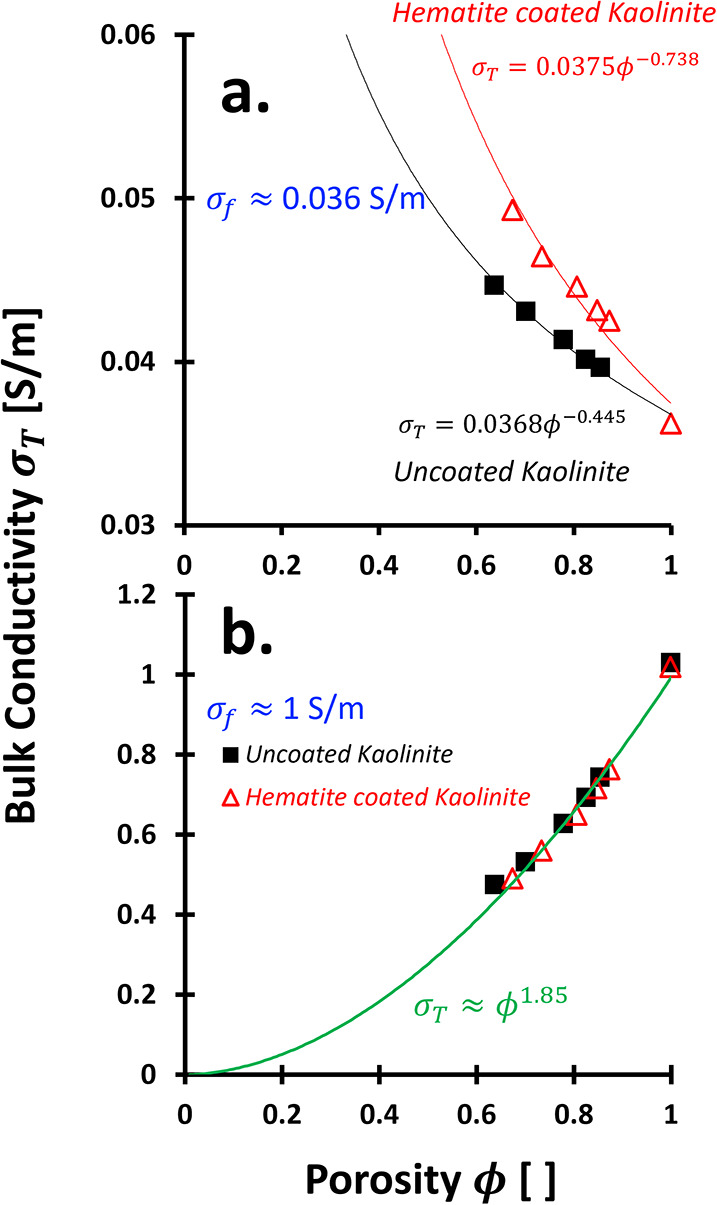
Hematite coating effects on bulk conductivity at (a) low and (b) high conductivity of pore fluids σ_f_. Data source: ref 260.

## 7 Electrical Properties and Beyond

This section explores the use of electrical conductivity and permittivity measurements to infer hydraulic properties that lack direct analytical relationships or causal links. The hydraulic properties of interest include transport-related parameters such as pore size distribution (inferred from capillary saturation curves, the J-function, or soil water characteristic curves), absolute permeability (measured in the liquid phase or corrected for Klinkenberg effects), and wettability (which influences relative permeability). It is essential to clearly differentiate between variables that directly influence these properties and those that do not.

### 7.1 Pore Size Distributions

As previously discussed, spatial polarization refers to the displacement of free ions and counterion clouds along wet mineral surfaces and pore walls, often linked to ion diffusion in confined geometries (Figure 13). This process implies that the dielectric relaxation time τ corresponds to diffusion time, with the pore size *r* representing the characteristic diffusion length, expressed as^26,37^

$$r=\sqrt{D\tau }$$ 29

where *D* is the diffusion coefficient. Spatial polarization may involve Stern layer effects observed in grains, pores, or planar surfaces, where the double-layer thickness also follows $\lambda_{D}=\sqrt{D\tau }$ .^291,292^ Relaxation time τ is identified from the peak in quadrature conductivity σ^″^ at frequencies below 10 Hz, with σ^″^ = ωε_0_κ^′^, linking it to real permittivity.

Empirical corrections introduce a scaling factor α_*D*_, yielding an effective pore size $r=\sqrt{\alpha_{D}D\tau }$ and effective diffusion coefficient *D*_eff_ = α_*D*_ *D*. The adjustment factor α_*D*_ may follow a power law with respect to peak pore size (α_*D*_ ∝ *r*_*peak*_^*n*_*r*_^)^293,294^ or depend on tortuosity (α_*D*_ ∝ 1/τ_*f*_^2^).^295^ However, this method captures only one dominant pore size, limiting its utility for full pore size distribution estimation in bimodal or multimodal systems.

A clearer method that links the pore size distribution to electrical conductivity may refer to the pore geometry and structure (PGS) rock typing: $\sqrt{k/\phi }$ vs *k*/ϕ^3^.^27^ Figure 28 reveals that lower rock type numbers *N* indicate larger pore throats $r\propto \sqrt{k/\phi }$ and higher bulk electrical conductivity σ_*B*_ ∝ exp (− η_*PGS*_ *N*), where the η_*PGS*_ is a positive fitting constant.

**Figure 28:**
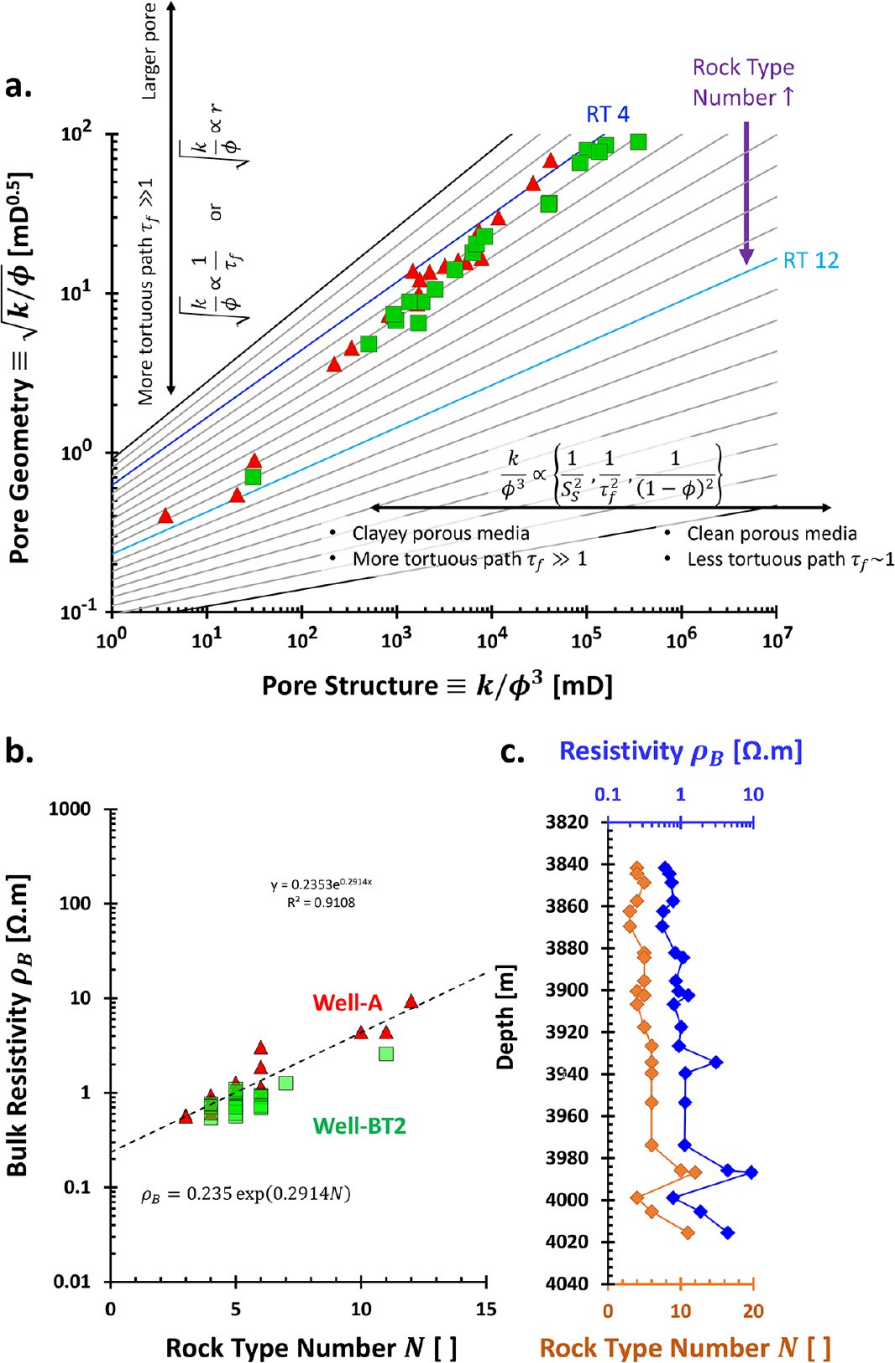
Pore geometry–pore structure rock typing and resistivity. (a) Rock typing yields in rock type number from the plot between $\sqrt{k/\phi }$ vs k/ϕ^3^. (b) Cross-plot between the bulk resistivity ρ_B_ vs rock type number N. (c) Resistivity and rock type number profiles within subsurface depth. RT stands for Rock Type. Data source: ref 27.

### 7.2 Permeability

The electrical conductivity σ_*T*_ of porous media is fundamentally governed by the intrinsic conductivities of its constituents: the fluid (σ_*f*_) and mineral matrix (σ_*m*_) along with the surface conductivity (Γ*S*_*S*_), porosity (ϕ), fluid saturation (*S*_*f*_), and pore structure characteristics such as tortuosity (τ_*m*_, τ_*f*_) and specific surface area (*S*_*S*_). In contrast, permeability *k* in [mD] or hydraulic conductivity *k*_*h*_ in [m/s] does not construct the electrical conductivity formulation. Although hydraulic properties such as porosity ϕ, pore radius *r*, tortuosity τ_*f*_, and specific surface area *S*_*S*_ are common to both phenomena, electrical and hydraulic conductivities arise from different physical mechanisms and must be treated independently.

Permeability can be expressed through classical models derived from the Hagen–Poiseuille equation, capillary bundle theory, and Darcy’s law, yielding:^27^

$$k=\frac{1}{c_{0}}\frac{\phi r^{2}}{8\tau_{f}^{2}}$$ 30

Alternatively, the Kozeny–Carman equation provides:^27^

$$k=\frac{c_{1}}{c_{0}}\frac{\phi^{3}}{32{\left(1-\phi \right)}^{2}}\frac{\alpha^{2}}{\tau_{f}^{2}\rho_{m}^{2}S_{S}^{2}}$$ 31

where variables [unit]: *r* [m], *k* [mD], ϕ [ ], τ_*f*_ [ ], ρ_*m*_ [g/cm^3^], *S*_*S*_ [m^2^/g]. The dimensionless geometric factor α = 2 for parallel sheet pores (e.g., kaolinite) and α = 4 for cylindrical pores. Conversion factors include *c*_1_= 10^–12^ m^6^/cm^6^ and *c*_0_= 9.869233 × 10^–16^ m^2^/mD. Notably, these expressions reinforce that electrical conductivity can be measured in fully saturated but hydraulically isolated systems (i.e., zero permeability), emphasizing the lack of a direct causal link between σ_*T*_ and *k*.

Equation (30) may be reformulated using the formation factor $F=\frac{\tau_{f}^{2}}{\phi^{m}}=\frac{\sigma_{f}}{\sigma_{T}}$ and *m* = 1, resulting in^27^

$$k=\frac{1}{c_{0}}\frac{r^{2}}{8F}=\frac{1}{c_{0}}\frac{r^{2}}{8}\frac{\sigma_{T}}{\sigma_{f}}\to k\propto \sigma_{T}$$ 32

Equation 32 implies a linear dependence of permeability on electrical conductivity, under the assumption of a uniform pore radius. However, empirical data sets (Figure 29) often show deviations from linearity, exhibiting instead a power-law behavior: *k* ∝ σ_*T*_^*n*_*s*_^ where *n*_*s*_ > 0 (see refs 27,101,296,297 ).

**Figure 29:**
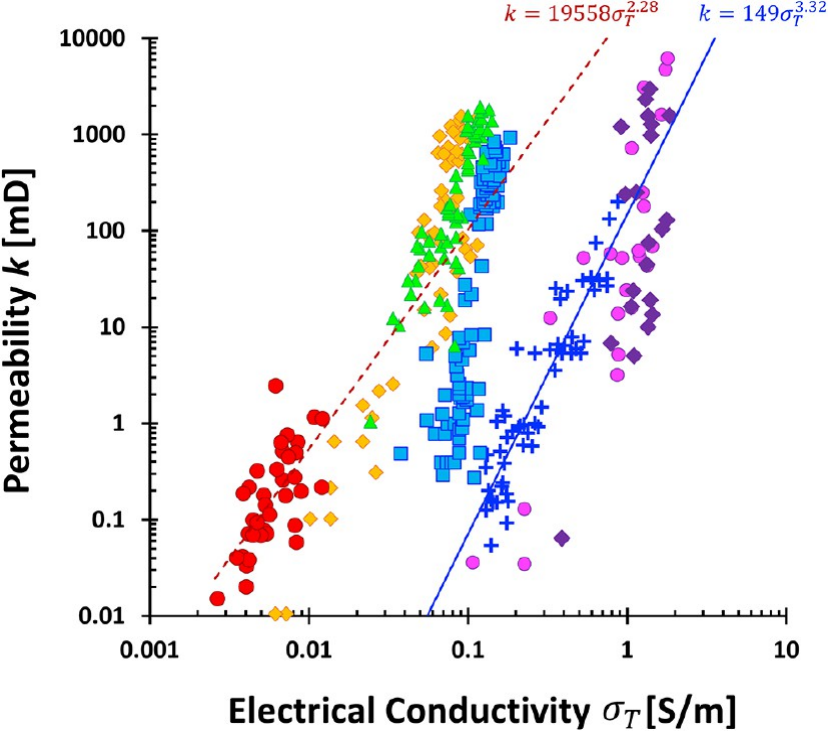
Relationship between electrical conductivity and permeability. Data sources: refs 27,101,296,297 . Conversions from formation factor *F* = σ_f_/σ_T_ use an assumed pore fluid conductivity σ_f_ of 1 S/m, unless the total conductivity σ_T_ is declared. Colors and shapes represent data set from the same formation.

If the pore radius is expressed as a function of the dielectric relaxation time τ using $r=\sqrt{D_{\mathrm{eff}}\tau }$ , eq 32 becomes

$$k=\frac{1}{c_{0}}\frac{D_{\mathrm{eff}}\tau }{8F}=\frac{1}{c_{0}}\frac{D_{\mathrm{eff}}\tau }{8}\frac{\sigma_{T}}{\sigma_{f}}\to k\propto \tau \sigma_{T}$$ 33

Here, τ corresponds to the inverse of the low-frequency critical frequency *f*_*c*, *LF*_, associated with spatial polarizations τ = 1/(2π*f*_*c*, *LF*_). Under the condition of constant σ_*T*_, a linear relation between *k* and τ is expected (*k* ∝ τ). This relation has theoretical backing in various derivations, such as *k* = *D*_eff_τ/9*F* ^3^ for *F* ≫ 1 ^298^ and *k* = *D*_eff_τ/4*F*.^299^ Nonetheless, numerous experimental studies reveal a more generalized power-law dependence: *k* ∝ τ^*n*_t_^ where 0.5 ≤ *n*_*t*_ ≤ 3.9 (see detailed summary in refs 294,300 ).

Although the relationship between permeability and dielectric relaxation time is fundamentally empirical and data set-dependent, a consistent trend can be identified: longer relaxation times (τ > 0.1 s) tend to correspond to clay-rich low-permeability media; while shorter relaxation times (τ < 0.06 s) are indicative of coarse and more permeable materials such as sands and gravels.^202^

### 7.3 Wettability

Wettability refers to the tendency of a porous medium’s surface to be preferentially wetted by a particular fluid. It is quantitatively characterized by the contact angle formed between a small liquid droplet and the solid surface.^51,301,302^ Wettability can be assessed using semiquantitative methods such as the residual saturation-based Amott index,^303,304^ the capillary pressure-based USBM index,^305,306^ and interpretations of relative permeability curves.^245,307^ Soils and rocks may exhibit water-wet (hydrophilic), oil-wet (hydrophobic), or intermediate (mixed-wet) behavior depending on their surface chemistry and fluid interactions.

#### Wettability vs Conductivity

Previous studies have explored the relationship between wettability and electrical conductivity.^305,308–310^ While a direct causal link has not been established, several key interactions should be considered: (i) Wettability affects saturation hysteresis during drainage and imbibition, influencing fluid distribution and flow. (ii) Electrical conductivity depends on phase connectivity and pore structure, and is often measured independently of flow. The relationship is thus indirect, governed by how wettability alters fluid configurations and interfacial interactions.

In wet porous media, **fluid content** primarily governs conductivity. However, **wettability** influences conductivity through **solid–fluid interactions**, particularly via **surface conduction**. For example, water-wet media filled with saline water exhibit higher conductivity due to synergistic bulk and surface ion transport. Conversely, in oil-wet media, water may be expelled from mineral surfaces at low saturations (e.g., *S*_*w*_ < 0.5), suppressing surface conduction and reducing overall conductivity. However, at high saturation greater than the threshold (*S*_*w*_ > *S*_*th*_ ), electrical conductivity of oil-wet media can be high due to a good pore-fluid connectivity (Figure 30).

**Figure 30:**
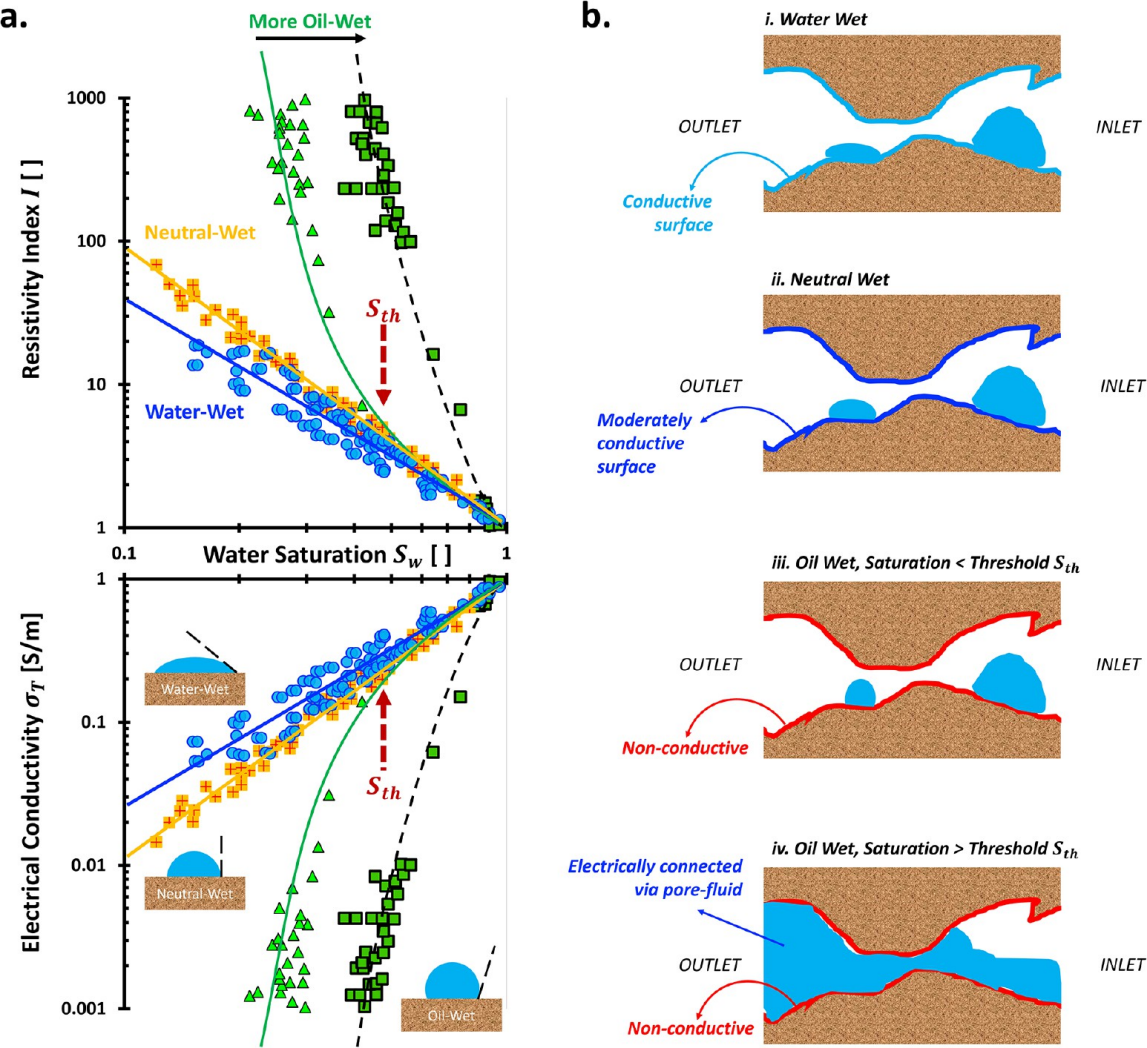
Wettability and electrical properties relationship. (a) Electrical properties evolution on degree of saturation based on wettability type. Conversions from resistivity index *I* = σ_T,1_/σ_T_ use assumed saturated-sample conductivity σ_T,1_ of 1 S/m. Data source: ref 308. Insets: illustrated contact angles formed by brine droplets on top of rock with different types of wettability. Angle measured is the inner part (brine-rock). The *S_th_* means a threshold saturation for oil-wet media. (b) Water droplets, adsorbed waters, and pore fluid connectivity inside pores based on the wettability type.

Conductivity in porous media arises from bulk fluid conduction (σ_*f*_) and surface conductivity (Γ*S*_*S*_) via the electrical double layer. When pores are disconnected, only solid matrix conduction (σ_*m*_) occurs, resulting in minimal total conductivity. Thus, meaningful conductivity–wettability relationships require connected pore networks, typically achieved above a threshold saturation *S*_*th*_ (Figure 30).

Notably, conductivity curves for water-wet and oil-wet samples may converge once pore connectivity with the wetting phase is established. This demonstrates that single-point conductivity data are insufficient to assess wettability; a saturation-dependent analysis (e.g., via drainage/imbibition tests or hysteresis curve) is essential.

We hypothesize that matched wettability and fluid type (e.g., water-wet rocks filled with brine) enhances ion transport and surface conduction. This effect is more pronounced in porous media with high specific surface area, whereas in low surface area materials (e.g., clean sands), conductivity is dominated by the pore fluid, with minimal impact from wettability.

#### Wettability vs Permittivity

There have been previous studies that investigated the influence of wettability on broadband permittivity spectra,^311–314^ but the interpretations remain complex and inconclusive. Multiple factors such as porosity, pore size distribution, geometry, saturation, fluid type, and specific surface area also affect interfacial and spatial polarization. Additionally, electrode polarization in the kHz–MHz range complicates the detection of spatial polarization, making it challenging to isolate the effect of wettability on permittivity responses (Figure 13b and Figure 18).

#### Wettability vs Zeta Potential

Conductivity and permittivity are bulk properties reflecting contributions from the solid matrix, pore fluid, and their interactions, whereas wettability specifically describes mineral–fluid interactions. For this reason, zeta potential ζ is a more suitable electrical indicator of wettability.

Zeta potential represents the electrostatic potential at the shear plane near the mineral surface—close to, but not at, the Stern layer (Figure 6b, read refs 42,70 ). Its polarity and magnitude correlate with surface–fluid affinity: for instance, a more negative ζ repels negatively charged oil species (e.g., RCOO^–^ ^51^), suggesting water-wet behavior. Oil and crude oils are negatively charged at pH values between 6 and 9, as they typically exhibit isoelectric points in the pH range of 3 to 6.^315^ However, ζ decreases with fluid saturation and approaches zero near irreducible water saturation.^316^

The **Helmholtz–Smoluchowski equation** used to estimate ζ requires corrections in saline and confined systems due to hindered water polarizability^267,317^ and the resulting reduced static permittivity.^147^ Surface conduction effects and low ionic strength also affect the accuracy of ζ.^65,68^ Competitions between surface and pore fluid conduction effects into total conductivity refer to Figure 22.

Additionally, ζ is pH-dependent and related to the isoelectric point (IEP), where ζ = 0, and the point of zero charge (PZC), where the surface charge is zero^318^ (Figure 7c). These may differ if specific ion adsorption occurs.^319^ Finally, diffuse double layer thickness λ_*D*_, which expands at lower ionic strengths, influences wettability by stabilizing water films on mineral surfaces and reducing oil affinity.^51,320^ Thus, wettability characterization is best approached using ζ and λ_*D*_ together.

## 8 Developments and Challenges

This section highlights recent advances in the measurement and analysis of the electrical properties of porous media. Innovations in sensor technology, IT, and mobile communication have transformed traditional methods—shifting from conduction (electrode-based^321,322^ ) to induction (noncontact^323^), from bulky instruments^324^ to portable devices,^325^ from simple dual-wire^216^ or four-electrode setups^321^ to multichannel automated systems,^326^ and time domain based (e.g., time domain reflectometry,^327,328^ time domain induced polarization^329^) and DC mode^321^ to broadband spectral measurements.^202,330^ These developments enable characterization of relaxation times and physical phenomena across various scales (e.g., in liquids^331^ and wet porous media^332^).

Electrical properties are directly influenced by hydraulic parameters such as porosity, saturation, tortuosity, salinity, and clay content. While indirect relationships (e.g., pore size distribution, permeability, and wettability) can be empirically modeled, they require physical validation to ensure realistic trends and values. The growing data set now supports the development of multidimensional electrical-hydraulic property databases across materials like membranes,^333^ catalysts,^334^ soils,^335,336^ and rocks.^27,37,337^

Due to the complexity and often nonreproducible nature of geomaterials, technologies like photolithography and nanofabrication have enabled the design of controlled porous systems (e.g., 3D-printed structures^338^ and microfluidic chips^264,339–342^ for reactive processes^343^ and flow^344,345^ ). These allow real-time visualization and reduce uncertainty in interpretation.

Advanced imaging methods (e.g., optical, electron microscopy, MRI, X-ray CT) further aid in monitoring processes, though they often generate large data sets requiring significant computational resources.^346^ AI tools including machine learning, deep learning, and neural networks accelerate data processing, inversion, and interpretation, especially for spatially proximate or similar materials.

Geoscience is well-positioned to address urgent global challenges, such as energy security, climate change, and sustainability. Opportunities include:

- Leveraging eco-friendly porous materials (e.g., cement,^347^ catalyst,^348^ geopolymers^349–352^ ) for environmental applications.
- Using electrical properties alongside multiphysics measurements (e.g., optical, magnetic, acoustic) for more robust in situ characterization.
- Updating testing protocols and simulation tools to reflect complex multiphysics coupled processes (hydro-chemo-biothermo-mechanical phenomena), high variability, long-term, repetitive loads, and extreme environmental conditions (read further ref^353^).
- Enhancing multiphase and dynamic characterizations due to changes from fluid displacements,^245^ phase transformations,^352^ or bulk deformations.^354^

## 9 Concluding Remarks

This paper summarizes the origin and measurements of electrical properties in geo-porous media, mainly conductivity and its associated equivalencies: resistivity and permittivity under isothermal conditions. The goal of sensing electrical properties is to infer the state and hydraulic properties of the porous media and its constituent. Our observations emphasize the following highlights:

- Pore fluid governs the conductive and polarizable traits of porous media because a dry porous medium is inherently nonconductive and nonpolarizable.
- Measurement methods on geo-porous media can be in multiple frequencies and depend on the investigated scales. Each frequency regime provides unique physical phenomena in which the lower frequency methods typically: (i) deploy a lumped circuit theory and require electrodes for the lab scale, and (ii) reach wider coverage or deeper skin depth for the electromagnetic wave method in the field settings. Meanwhile, the high-frequency measurements use a wave propagation method, can be electrodeless, and achieve a smaller scale yet high spatial resolution.
- Measurements from low-to-high frequency capture following electrical polarizations: Conduction, electrode, spatial, orientational, vibrational ionic, and electronic polarizations.
- The engineering implications and possible inferred state/hydraulic properties for each electrical polarization are as follows:
  Conduction: Porosity, specific surface area, water content, salinity, turbidity, clay type/content, metal content, pore size, permeability, and wettability
  Electrode: Fluid type, clay type/content, and salinity
  Spatial: Pore size, pore structure, permeability
  Orientational: Porosity, water content, salinity, turbidity, fluid type
  Vibrational Ionic and Electronic: Fluid-bonding type and characterization
- These relationships may not be solely physically related or in a direct causality but rather data driven:
  Dielectric relaxation time and transport properties (pore size distribution, permeability, wettability)
  DC electrical conductivity and permeability
  DC electrical conductivity and wettability.
- Electrical conductivity is about the phase and pore connectivity so that ion transport is possible; and is often measured regardless of the flow, whereas permeability is a measure of the porous media to allow certain phases to flow. Porosity, pore size, specific surface area, and tortuosity independently contribute to electrical conductivity and permeability. However, no direct physical relationship exists in which permeability influences electrical conductivity.
- Wettability type is an interface phenomenon between solid mineral—pore fluid interactions. Therefore, zeta potential is the more suitable property that characterize a wettability, instead of bulk electrical properties: conductivity and permittivity.
- In the low-frequency regime, the polarization indicated by high real permittivity and measured by the four-electrode method may be debatable whether it is due to electrode, diffuse double layer, and/or Stern layer polarizations. Our summary is prone to consider this due to electrode polarizations with clear notices: the four-electrode method produces one-to-3 orders of magnitude smaller in the real permittivity and one-to-2 orders of magnitude less in the electrode polarization limiting frequency; compared to the results obtained by the two-electrode method. Both electrode and spatial polarizations can coexist in a low-frequency regime.
- Research on proper and prudent interpretations for each polarization type can carefully be evaluated on conditioned fabricated porous media, e.g., microfluidic chips, to obtain controlled and precisely quantified parameters such as porosity, surface roughness, pore size distribution, permeability, wettability, and saturation.

## Supplementary Material



## Notes

Data for figures (.xlsx) and high-resolution figures are available in the NYCU repository: https://dataverse.lib.nycu.edu.tw/dataverse/hakiki and https://doi.org/10.57770/P2UX1Z (PDF)
