# Supplementary material for: Electrical Conductivity and Permittivity of Porous Media: Origin, Measurements, and Implications
Source: ACS Meas Sci Au. 2025 Sep 1;5(5):595–635. doi: 10.1021/acsmeasuresciau.5c00070 (PMC12532063; doi:10.1021/acsmeasuresciau.5c00070)
Supplement: Supplementary file 1 [file tg5c00070_si_001.pdf]

SUPPORTING INFORMATION

Electrical Conductivity and Permittivity of Porous Media: Origin, Measurements, and Implications

Authors:

Farizal Hakiki [a,b]*, Chih-Ping Lin [a,b]

Affiliations:

[a] National Yang Ming Chiao Tung University (NYCU), Disaster Prevention & Water Environment Research Center (DPWE), Hsinchu 300, Taiwan

[b] National Yang Ming Chiao Tung University (NYCU), Civil Engineering Department, Hsinchu 300, Taiwan

*Corresponding author: F. Hakiki (hakiki@nycu.edu.tw; alhakiki@live.co.uk),

Legal name: Farizal Hakiki Soemarsono

Data availability:

Data for main figures (.xlsx) and high-resolution figures are available in the NYCU repository:

https://dataverse.lib.nycu.edu.tw/dataverse/hakiki and

https://doi.org/10.57770/P2UX1Z

Data developed from models are not all provided but we describe the parameters used

in the models that are available in the corresponding figures' captions.
